# Experimental and computational study of alkane dehydrogenation catalyzed by a carbazolide-based rhodium PNP pincer complex†
†Electronic supplementary information (ESI) available: General experimental details, procedures, and data; images of NMR spectra for new compounds; computational details; structures, energies, and .mol files for computational species. See DOI: 10.1039/c5sc04794c


**DOI:** 10.1039/c5sc04794c

**Published:** 2016-01-20

**Authors:** David Bézier, Changjian Guan, Karsten Krogh-Jespersen, Alan S. Goldman, Maurice Brookhart

**Affiliations:** a Department of Chemistry , The University of North Carolina at Chapel Hill , Chapel Hill , North Carolina 27599 , USA; b Department of Chemistry and Chemical Biology , Rutgers , The State University of New Jersey , New Brunswick , New Jersey 08903 , USA

## Abstract

A rhodium complex based on the bis-phosphine carbazolide pincer ligand was investigated in the context of alkane dehydrogenation and in comparison with its iridium analogue. (carb-PNP)RhH_2_ was found to catalyze cyclooctane/*t*-butylethylene (COA/TBE) transfer dehydrogenation with a turnover frequency up to 10 min^–1^ and turnover numbers up to 340, in marked contrast with the inactive Ir analogue. TONs were limited by catalyst decomposition. Through a combination of mechanistic, experimental and computational (DFT) studies the difference between the Rh and Ir analogues was found to be attributable to the much greater accessibility of the 14-electron (carb-PNP)M(i) fragment in the case of Rh. In contrast, Ir is more strongly biased toward the M(iii) oxidation state. Thus (carb-PNP)RhH_2_ but not (carb-PNP)IrH_2_ can be dehydrogenated by sacrificial hydrogen acceptors, particularly TBE. The rate-limiting segment of the (carb-PNP)Rh-catalyzed COA/TBE transfer dehydrogenation cycle is found to be the dehydrogenation of COA. Within this segment, the rate-determining step is calculated to be (carb-PNP)Rh(cyclooctyl)(H) undergoing formation of a β-H agostic intermediate, while the reverse step (loss of a β-H agostic interaction) is rate-limiting for hydrogenation of the acceptors TBE and ethylene. Such a step has not previously been proposed as rate-limiting in the context of alkane dehydrogenation, nor, to our knowledge, has the reverse step been proposed as rate-limiting for olefin hydrogenation.

## Abstract 2

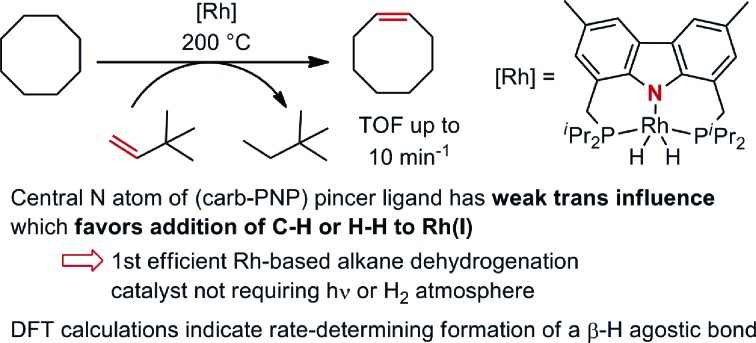
A rhodium complex based on the bis-phosphine carbazolide pincer ligand was investigated in the context of alkane dehydrogenation and in comparison with its iridium analogue.

## Introduction

Olefins are highly versatile intermediates which can be converted to a wide array of products such as detergents, pharmaceutical intermediates, lubricants, fuels and polymers.^1^ Accordingly, there has been growing interest in the homogeneous dehydrogenation of alkanes as a potential highly atom-economic route to olefins.

The first catalytic transfer alkane dehydrogenations were reported independently by Felkin and Crabtree using phosphine-based rhenium, ruthenium and iridium catalysts for a reaction that has become standard for screening transfer dehydrogenations, the use of *t*-butylethylene (TBE) as a hydrogen acceptor to dehydrogenate cyclooctane (COA).^2^ Turnover numbers (TONs) in these systems were limited (<100 TO) by low catalyst stability. Following these reports, rhodium-based systems were developed independently by the groups of Saito,^3^ Tanaka^4^ and Goldman^5^ which exhibited high TONs for alkane dehydrogenation; however, formation of the active species, Rh(Cl)(PR_3_)_2_, could only be achieved photochemically^6^ or under H_2_ atmosphere, limiting the utility of these systems.

**Fig. 1:**
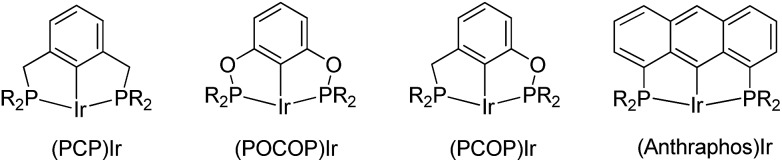
Examples of active PCP iridium pincer complexes for alkane dehydrogenation.

The development of the iridium pincer complex (^*t*Bu_4_^PCP)IrH_2_ by Kaska and Jensen was a breakthrough for the achievement of high TONs in the benchmark COA/TBE system.^7^ More active and stable iridium complexes were next developed through modification of the pincer ligand. Catalysts based on the PCP,^8^ POCOP,^9^ PCOP,^10^ anthraphos10a,^11^ (Fig. 1) and other^12^ frameworks have since been used and studied extensively.

**Scheme 1:**
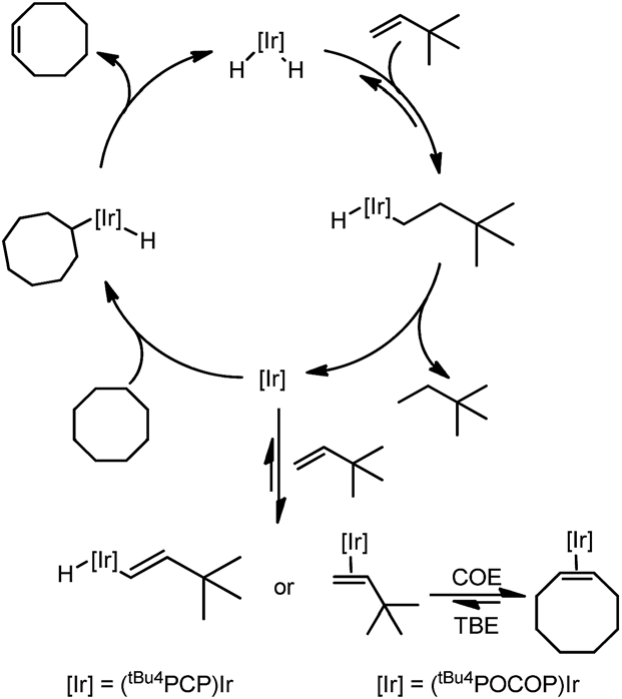
Mechanism of transfer dehydrogenation of COA with TBE using PCP- and POCOP–iridium complexes.

The mechanism of the transfer dehydrogenation of COA with TBE using PCP^13^ and POCOP8a,b iridium pincer complexes has been thoroughly investigated. The overall catalytic cycle is shown in Scheme 1.

The mechanisms for these two systems are similar. Beginning with the 16-electron iridium dihydride complex, insertion of TBE yields the alkyl hydride complex which undergoes reductive elimination to form the Ir(i) 14-electron species. This complex activates the C–H bond of cyclooctane, followed by β-hydride elimination to yield cyclooctene and regenerate the iridium dihydride. At low concentration of TBE, hydrogenation is turnover-limiting for the (PCP)Ir system, and the resting state is (PCP)IrH_2_, while at high [TBE], COA dehydrogenation is turnover-limiting and the resting state is the vinyl hydride complex. For the (POCOP)Ir system, dehydrogenation is turnover-limiting and alkene (TBE and COE) complexes are the resting states.

DFT calculations have been conducted^14^ which indicate that more weakly σ-donating groups at the central position of the pincer ligand favor the thermodynamics of C–H (and H–H) addition to the 14e pincer-Ir fragments. Intrigued by the possible implications for alkane dehydrogenation, we recently synthesized **1-C_2_H_4_**, an Ir complex of the bis-phosphine carbazolide pincer, carb-PNP, in which the central coordinating group is an sp^2^ nitrogen which is much less σ-donating than the sp^2^ carbon of PCP pincer ligands. In a previous study, however, **1-C_2_H_4_** was found to be ineffective as a catalyst for alkane transfer dehydrogenation (eqn (1)).^15^1
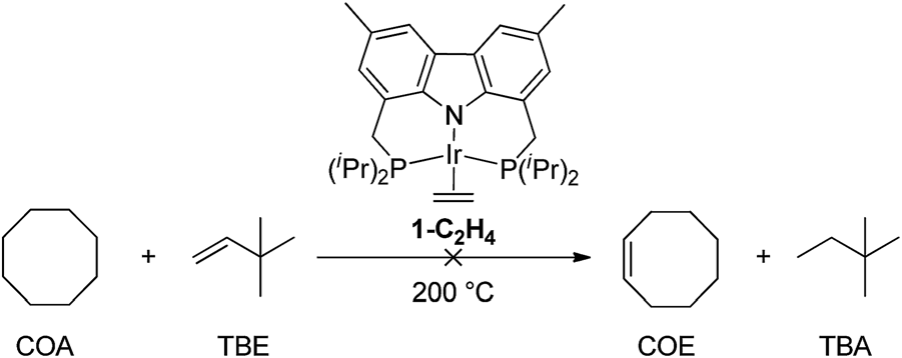



Experimental and computational studies indicated that hydrogenation of TBE was the rate-limiting step for **1**-catalyzed COA/TBE transfer dehydrogenation. TBE did insert into an Ir–H bond of **1-H_2_**, but reductive elimination of alkane from the resulting Ir(iii) alkyl hydride, **1-H(C_2_H_4_^*t*^Bu)**, was thermodynamically very unfavorable (eqn (2)). Thus, compared with PCP ligands, the carb-PNP ligand was indeed found to strongly favor the Ir(iii) alkyl hydride, as well as the Ir(iii) dihydride, relative to the 14-electron Ir(i) fragment. But while C–H addition and alkane dehydrogenation by the 14-electron Ir species were favoured by the carb-PNP ligand, the hydrogenation segment of the cycle was disfavored so strongly that catalytic transfer-dehydrogenation was precluded.2
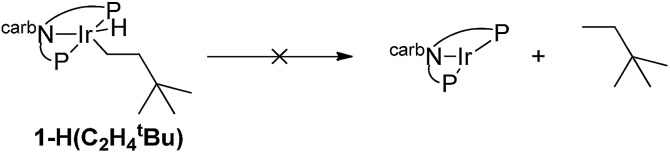



It was previously shown8*a* that in the case of PCP-type pincer ligands, the Rh(iii) state was not sufficiently accessible to allow an effective catalytic cycle based on the Rh(i)/Rh(iii) couple. Based on the conclusions reached in the studies with Ir(i), we considered that for carb-PNP complexes of rhodium, the Rh(iii) state should be relatively more favorable and thus the system might be active for alkane dehydrogenation.

Here we report that we have synthesized the rhodium complexes (carb-PNP)Rh(ethylene), **2-C_2_H_4_**, and (carb-PNP)Rh(H)_2_, **2-H_2_**, and studied, experimentally and computationally, their hydrogenation of ethylene and TBE, in analogy with the previous study of the (carb-PNP)Ir complexes.^15^ These complexes were also investigated for catalytic alkane transfer dehydrogenation. In contrast to the (carb-PNP)Ir analogues, and in accord with the hypothesis proposed above, we find them to be quite active as catalysts for COA/TBE transfer-dehydrogenation.

## Results and discussion

### Synthesis of (carb-PNP)Rh(ethylene) **2-C_2_H_4_**

The bis-phosphine carbazole ligand was synthesized following our previously reported procedure.^15^ After deprotonation of the ligand with LiN(TMS)_2_ and addition of [(C_2_H_4_)_2_RhCl]_2_, the solution turned dark brown. Filtration, followed by evaporation of the solvent gave a brown solid which was washed multiple times with cold *n*-octane and dried under vacuum to yield (carb-PNP)Rh(ethylene), **2-C_2_H_4_**, as a yellow solid (^31^P{^1^H} NMR: *δ* = 44.12 (d, *J*_P–Rh_ = 130 Hz)) (eqn (3)).^16^3
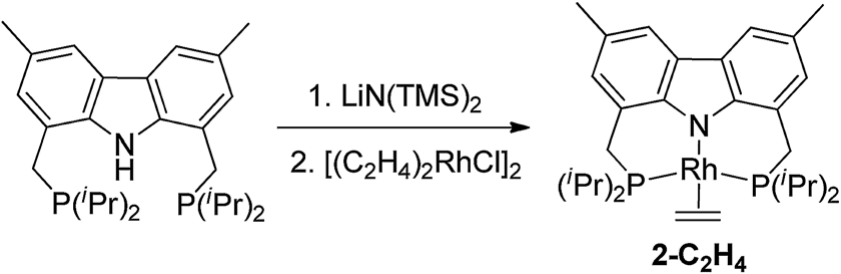



Addition of CO to **2-C_2_H_4_** at rt resulted in quantitative formation of the monocarbonyl complex (carb-PNP)Rh(CO), **2-CO** (^31^P{^1^H} NMR: *δ* = 54.68 (d, *J*_P–Rh_ = 125 Hz); IR *ν*(CO) = 1954 cm^–1^) (eqn (4)).4
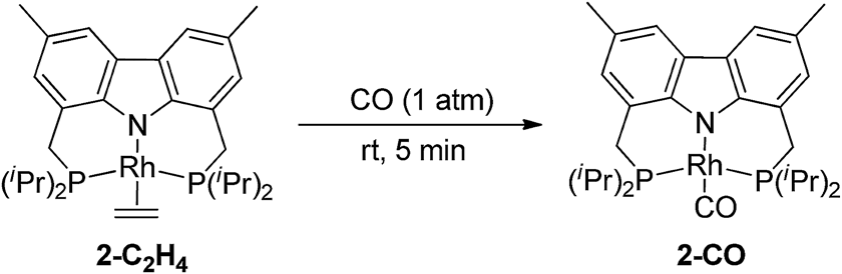



### Hydrogenation of ethylene and TBE using complexes **2-C_2_H_4_** and **2-H_2_**

Purging complex **2-C_2_H_4_** with H_2_ at rt for 10 min resulted in the complete conversion to (carb-PNP)Rh(H)_2_, **2-H_2_** (^31^P{^1^H} NMR: *δ* = 65.70 (d, *J*_P–Rh_ = 121 Hz); ^1^H NMR: –19.69 (q, *J*_P–Rh_ = 16 Hz, 2H)) (eqn (5)).5
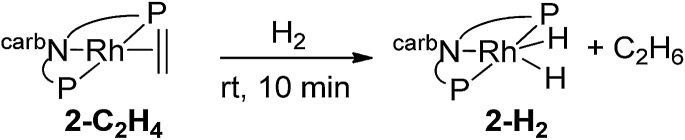



Under 1 atm of ethylene, **2-H_2_** was rapidly converted to **2-C_2_H_4_** at rt (eqn (6)). This behaviour is in marked contrast to the iridium analogue **1-H_2_** which requires a temperature of 70 °C with a half-life of 45 min for the analogous reaction (eqn (7)).^15^6
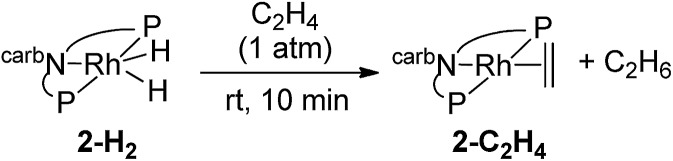

7
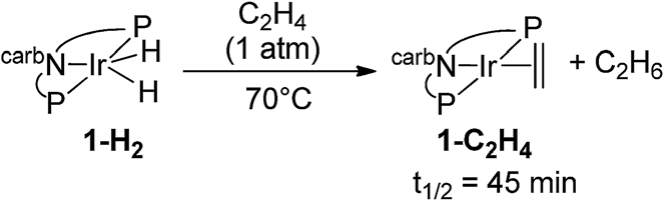



We next investigated the hydrogenation of TBE by **2-H_2_** (eqn (8)). No reaction was detected after 3 h at rt. However, after heating at 80 °C for 3 h, **2-H_2_** was converted to (carb-PNP)Rh(TBE), **2-TBE**, (^31^P{^1^H} NMR: *δ* = 26.21 (dd, *J*_P–P_ = 346 Hz, J_P–Rh_ = 140 Hz), *δ* = 13.26 (dd, *J*_PP_ = 347 Hz, J_P–Rh_ = 130 Hz)).^17^ Thus hydrogenation of TBE, like ethylene, by **2-H_2_** is much more facile than by iridium dihydride **1-H_2_**, the latter showing no reactivity after 10 h at 100 °C under the same conditions (eqn (9)).^15^8
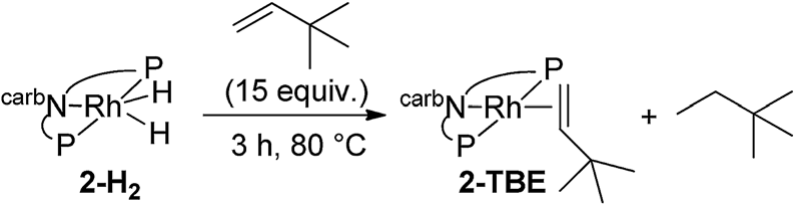

9
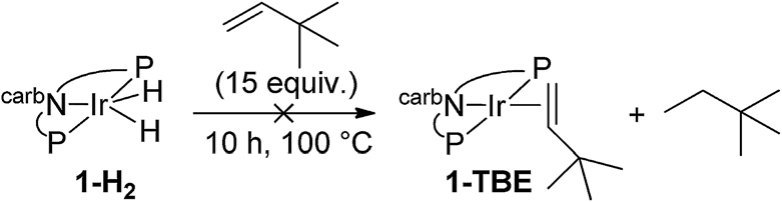



Under an atmosphere of dihydrogen, a solution of **2-H_2_** and TBE showed no reaction after 3 h at room temperature (eqn (10)). This behaviour contrasts with the iridium dihydride **1-H_2_** which, despite of the lack of reaction in the absence of H_2_, rapidly catalyzes the hydrogenation of TBE to TBA at room temperature. This hydrogenation was demonstrated to proceed *via* an Ir(iii)/Ir(v) catalytic cycle (eqn (11))^15^ with H_2_ playing a critical role of promoting reductive elimination of alkane from the iridium center through formation of an Ir(v) trihydride intermediate. An analogous mechanism is apparently not operative for the rhodium system.10
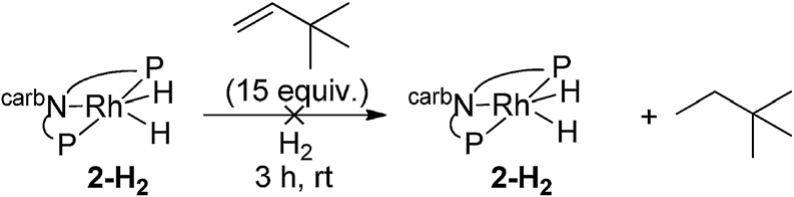

11
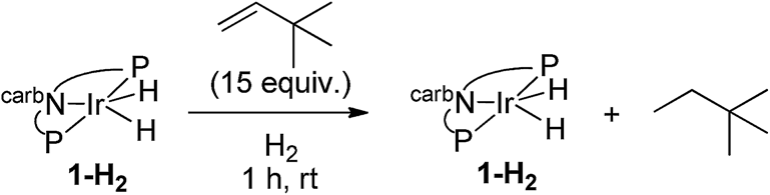



The mechanism of the hydrogenation of ethylene by **2-H_2_** (eqn (6)) was investigated. Addition of excess ethylene to rhodium dihydride **2-H_2_** at –88 °C gave Rh(iii) ethylene *cis*-(dihydride) complex **2-H_2_(C_2_H_4_)** (^31^P{^1^H} NMR: *δ* = 84.23 (bs); ^1^H NMR: –11.07 (bs, 1H), –20.22 (bs, 1H)) (eqn (12)). Binding of ethylene to **2-H_2_** to give **2-H_2_(C_2_H_4_)** is reversible and thermodynamically favored at low temperature (–88 °C to –70 °C). The free energy barrier for exchange of free ethylene with **2-H_2_(C_2_H_4_)** was estimated to be ≈9 kcal mol^–1^ based on NMR line broadening of the Ir–H signals at –80 °C. The dissociation of ethylene is easier from the rhodium ethylene dihydride complex than from the iridium analogue for which the barrier to dissociation was found to be ≈14 kcal mol^–1^ at 0 °C.^15^12
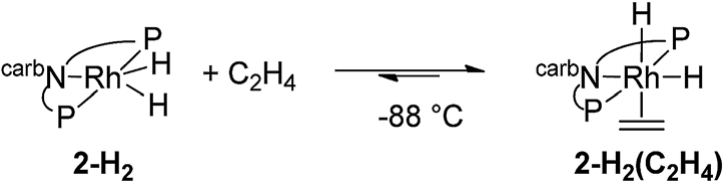



The rate of the stoichiometric hydrogenation of C_2_H_4_ by **2-H_2_** was measured at different concentrations of C_2_H_4_ at –30 °C (eqn (13)). The rate was found to be first-order in **2-H_2_(C_2_H_4_)**, but otherwise independent of the concentration of C_2_H_4_ in the range 0.05–0.5 M. A first-order rate constant, *k*, of 2.4 × 10^–4^ s^–1^ was obtained, corresponding to Δ*G*^‡^ = 18 kcal mol^–1^ at –30 °C.13
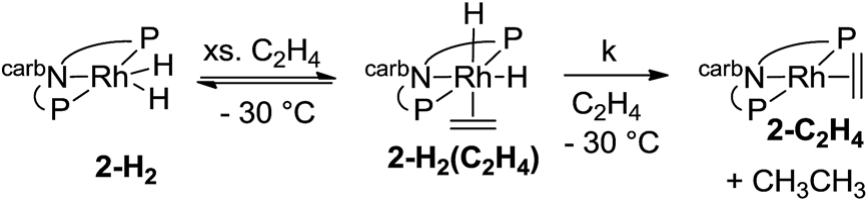



In the case of the hydrogenation of ethylene by the iridium complex **1-H_2_(C_2_H_4_)**, Δ*G*^‡^ was determined to be *ca.* 26 kcal mol^–1^ at a temperature of 75 °C. In contrast with the reaction of **2-H_2_(C_2_H_4_)**, the rate of reaction of **1-H_2_(C_2_H_4_)** was found to be dependent on the concentration of C_2_H_4_; this implied that another molecule of ethylene was involved in the reductive elimination of ethane from **1-(H)(Et)**, analogous to the promotion of reductive elimination by H_2_ from the same species.^15^

Deuterium labelling experiments were conducted to determine whether the rate-limiting step during the hydrogenation of ethylene (eqn (14)) was the migratory insertion, converting **2-H_2_(C_2_H_4_)** to **2-H(Et)**, or the reductive elimination (**2-H(Et)** to **2-C_2_H_4_**). To a solution of deuterium-labeled **2-D_2_(C_2_H_4_)**, an excess of ethylene was added at –80 °C and the reaction was gradually warmed up to –30 °C while monitored by NMR spectroscopy (eqn (15)). No evidence of H exchange into the Rh-D positions of **2-D_2_(C_2_H_4_)** was detected prior to formation of **2-C_2_H_4_** plus ethane, suggesting that the migratory insertion is irreversible and is the rate-limiting step with Δ*G*^‡^ = 18 kcal mol^–1^.^18^14
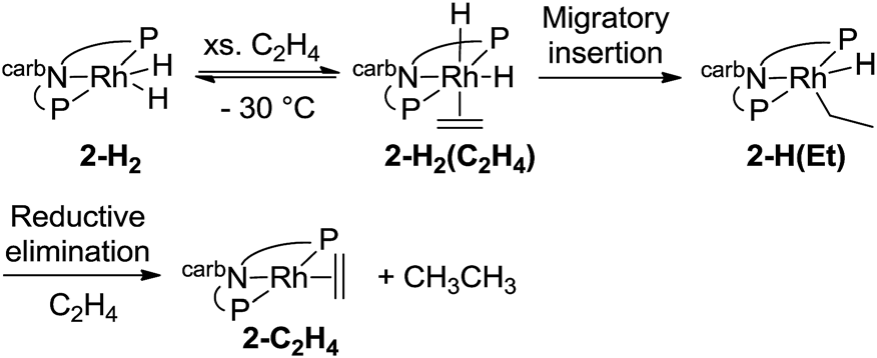

15
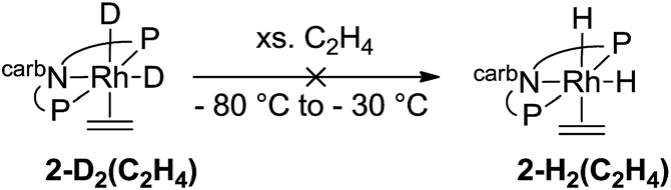



Since the Rh(iii) intermediate **2-H(Et)** was not detected, the barrier to reductive elimination of ethane from **2-H(Et)** must be less than 18 kcal mol^–1^. In fact this energy barrier was calculated by DFT to be quite low (Δ*G*‡calc = 6 kcal mol^–1^). As anticipated, the reductive elimination of ethane from the rhodium complex is more facile than for the iridium analogue which has a high kinetic barrier (Δ*G*^‡^ = 20–25 kcal mol^–1^).^15^

### Alkane dehydrogenation using rhodium dihydride complex **2-H_2_**

The reaction of (carb-PNP)RhH_2_ with TBE contrasted with the behaviour of the Ir analogue which showed no reaction with TBE in the absence of a H_2_ atmosphere. This led us to test complex **2-H_2_** as a catalyst for transfer dehydrogenation using the benchmark COA/TBE system (Table 1).

**Table 1:** TONs for the transfer dehydrogenation of COA and TBE catalyzed by **2-H_2_**^*a*^

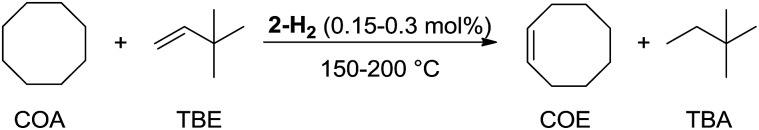				
Entry	Catalyst loading (mol%)	*T* (°C)	*t* [min]	TON
1	0.3	200	5	47
2	0.3	200	10	80
3	0.3	200	30	149
4	0.3	200	60	213
5	0.3	200	120	245
6	0.3	200	180	260
7	0.15	200	120	285
8	0.15	200	240	340
9	0.3	150	60	12
10	0.3	150	7200	220

^*a*^TONs were calculated based on conversion of TBE determined by GC analysis. COA (2.33 mmol), TBE (2.33 mmol), **2-H_2_** (3.42–6.83 μmol).

In contrast with the inactive iridium analogue, **2-H_2_** showed high activity for COA/TBE transfer-dehydrogenation at 200 °C. With 0.3 mol% catalyst loading of **2-H_2_**, 47 TONs were obtained after 5 min (TOF ≈ 10 min^–1^, entry 1) and the resting states detected by ^31^P NMR at 200 °C were (carb-PNP)Rh(TBE) **2-TBE** and (carb-PNP)Rh(H_2_) (**2-H_2_**) with a **2-TBE**/**2-H_2_** ratio of 3 : 1. The catalytic activity decreased over time with TONs of 149 after 30 min (entry 3) and 260 after 3 h (entry 6) corresponding to 44% and 76% conversion respectively. Longer reaction times do not afford increased TONs which suggests that the catalyst had decomposed. Accordingly, no ^31^P NMR signals were detected after 3 h. With a catalyst loading of 0.15 mol%, slightly higher TONs were obtained, up to 340 after 4 h at 200 °C (entry 8). The rate of the reaction dropped dramatically when the temperature was decreased to 150 °C, with TONs of 12 and 220 obtained after 1 h (entry 9) and 120 h (entry 10), respectively. For all these reactions the same rates and TONs obtained using **2-H_2_** were also obtained with the use of **2-C_2_H_4_** as a catalyst precursor.

**2-H_2_** was significantly less effective for the transfer dehydrogenation of *n*-octane (Table 2) than for COA. A 1 : 1 *n*-octane : TBE solution of **2-H_2_** (0.3 mol%) gave only 4 TOs after 5 min (TOF ≈ 1 min^–1^) at 200 °C (entry 1). The resting states detected by ^31^P NMR after 5 min were (carb-PNP)Rh(TBE) **2-TBE** and (carb-PNP)RhH_2_ (**2-H_2_**) with a ratio **2-TBE**/**2-H_2_** of 3 : 1, the same as observed in the COA/TBE system. The complex (carb-PNP)Rh(1-octene) was not detected, arguing against product inhibition as the explanation underlying the slow rate with *n*-octane.

**Table 2:** TONs for the transfer dehydrogenation of *n*-octane and TBE catalyzed by **2-H_2_**^*a*^

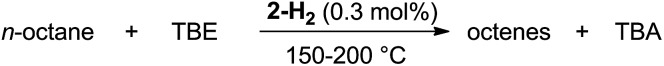			
Entry	*T* (°C)	*t* [min]	TON
1	200	5	4
2	200	30	13
3	200	60	14
4	150	60	<1
5	150	120	1
6	150	180	3

^*a*^TONs were calculated based on conversion of TBE determined by GC analysis. *n*-Octane (2.33 mmol), TBE (2.33 mmol), **2-H_2_** (6.83 μmol).

Decomposition of the catalyst limited the TON to 14 after 1 h (entry 3). The 1-octene isomer represented 16% of all octenes after 1 h. Calculations indicate a very slight kinetic preference for formation of 1-octene (0.7 kcal mol^–1^ at 200 °C) so this suggests that olefin isomerization is competitive with alkane dehydrogenation. Lowering the temperature to 150 °C decreased the reaction rate to ≈0.5 TON per h (entry 5).

### Computational results

DFT calculations were conducted on the reactions discussed above using the M06-L density functional and valence basis sets of triple-zeta plus polarization quality (see ESI†). We used a model ligand in which the two i-Pr groups on each P atom were replaced with a *t*-Bu and a methyl group to give a *C*_2_ symmetric diastereomer. Since metal-bound P^i^Pr_2_ groups typically adopt a conformation in which one of the two methine C–H bonds points toward the metal center while the other points away, the P^*t*^BuMe group mimics the steric effect of the P^i^Pr_2_ group. The P^*t*^BuMe group, however, offers the advantage of avoiding the many local (non-global) conformational minima which we have encountered in calculations of pincers with P^i^Pr_2_ groups (see ESI† for a computational assessment of this model). In addition, our model does not include the two methyl groups at the positions *para* to the carbazolide N atom. We refer to this ligand as carb-PNP′ and the model compounds as derivatives of **2′** to distinguish them from the experimental complexes of **2**.

**Fig. 2:**
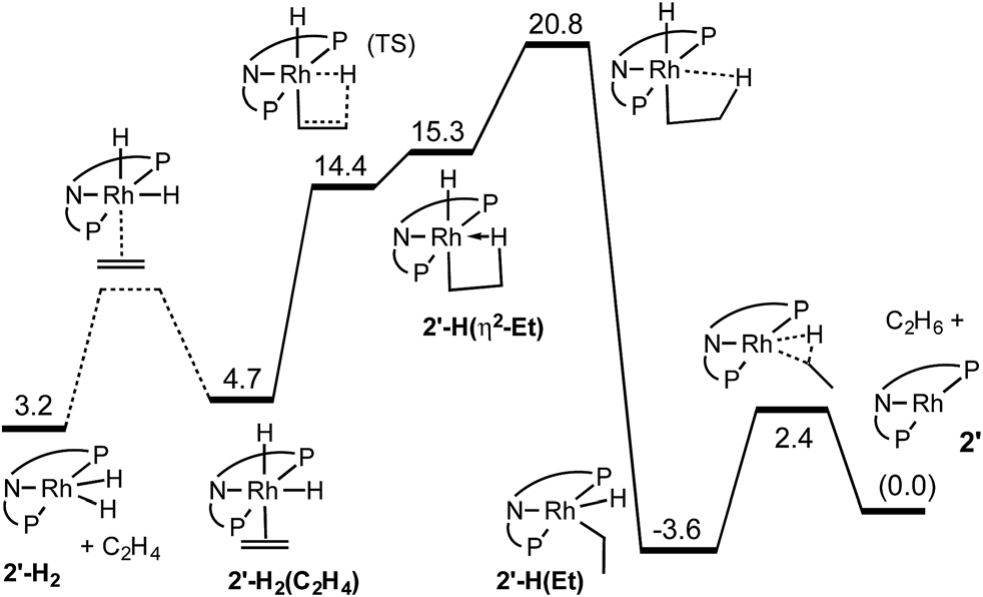
Calculated free energies (kcal mol^–1^) for reaction of **2′-H_2_** with ethylene at –30 °C.

The results of the calculations proved quite valuable in attempting to interpret the experimental results. A free energy diagram for the reaction of dihydride **2′-H_2_** with ethylene at –30 °C is shown in Fig. 2. Note that at –80 °C, the calculations indicate that ethylene binds to **2′-H_2_**, to give **2′-H_2_(C_2_H_4_)**, exoergically (Δ*G* = –0.7 kcal mol^–1^), in agreement with the observation illustrated in eqn (12). At –30 °C, the observed equilibrium suggests that Δ*G* is slightly positive and indeed, the calculated free energy of binding is Δ*G* = 1.5 kcal mol^–1^. At –30 °C, the barrier to the reaction of ethylene dihydride complex **2′-H_2_(C_2_H_4_)** to give the three-coordinate (carb-PNP′)Rh (**2′**) and ethane is calculated to be 16.1 kcal mol^–1^, in good agreement with the experimental value (for **2-H_2_(C_2_H_4_)**) of Δ*G*^‡^ = 18 kcal mol^–1^.

**Fig. 3:**
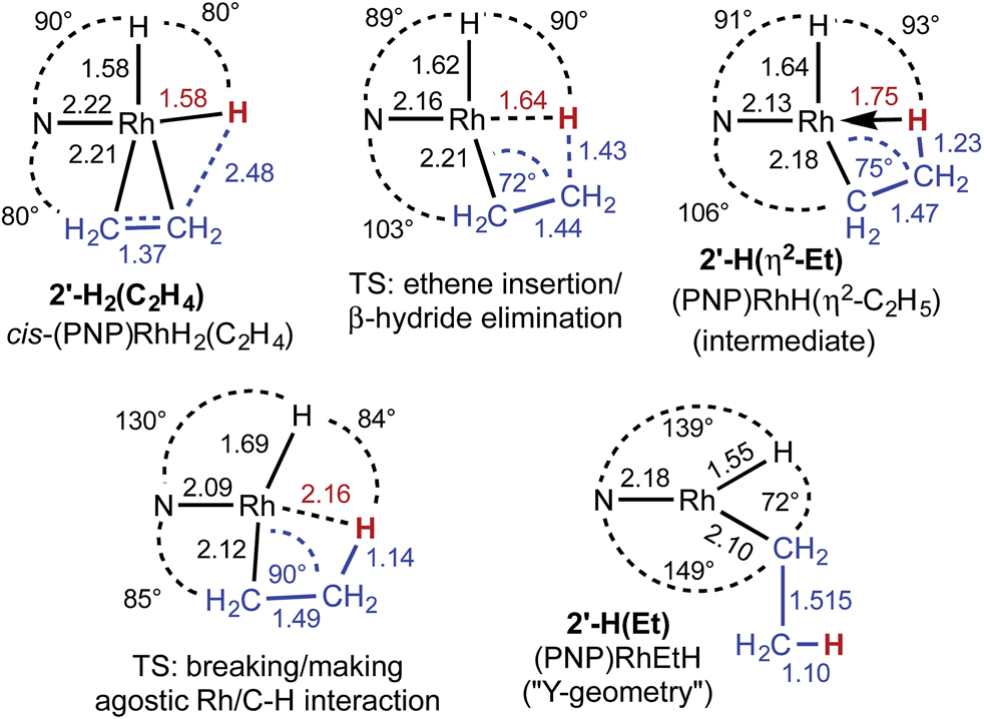
Structural parameters, in the plane bisecting the P–Rh–P axis, along the pathway for the insertion of ethylene into a Rh–H bond of **2′-H_2_(C_2_H_4_)**.

**Fig. 4:**
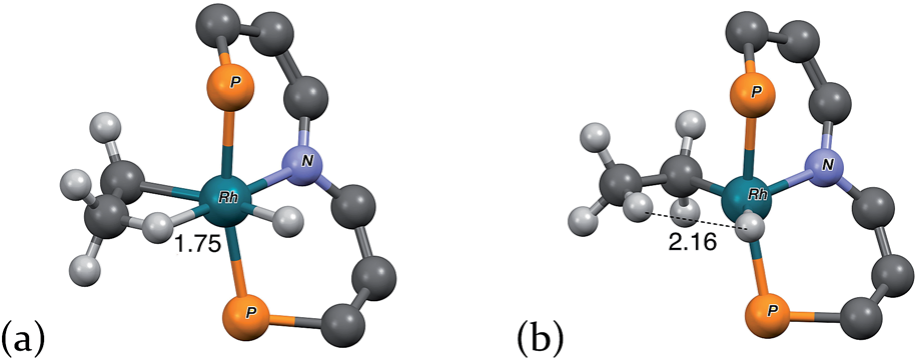
“3-D” models of (a) agostic intermediate **2′-H(η^2^-Et)** and (b) ring-opening transition state **TS(2′-H(η^2^-Et)/2′-H(Et))**. Peripheral atoms omitted for clarity. Rh–H distances in Å.

As indicated in eqn (15), H/D exchange of **2′-D_2_(C_2_H_4_)** with C_2_H_4_ is not observed in the course of the hydrogenation reaction. This would typically be interpreted to suggest that migratory insertion of ethylene is irreversible and is the rate-limiting step of the reaction, followed by fast elimination of ethane. The calculations, however, yield a somewhat different explanation. Insertion of C_2_H_4_ into a Rh–H bond of **2′-H_2_** leads to a β-agostic ethyl complex (PNP)RhH(η^2^-Et), **2′-H(η^2^-Et)**, with a nearly fully formed C–H bond (*d* = 1.23 Å; see Fig. 3 and 4).^19^ The TS for this insertion process at –30 °C has a free energy 9.7 kcal mol^–1^ above that of **2′-H_2_(C_2_H_4_)** while the free energy of the agostic product is 10.6 kcal mol^–1^ higher than **2′-H_2_(C_2_H_4_)** (although it has a lower free energy, *G*, the electronic energy, *E*, of the TS leading to the agostic intermediate is higher than that of the agostic intermediate, as required of a proper TS on the potential energy surface). Accordingly, the barrier to the back-reaction of this process (*i.e.***2′-H(η^2^-Et)** → **2′-H_2_(C_2_H_4_)**) is negligible.

The short Rh–H distance of 1.75 Å in **2′-H(η^2^-Et)** indicates a very strong agostic interaction. The H atom is located *trans* to the weak-*trans*-influence carbazole nitrogen, while the α-carbon is *trans* to a strong-*trans*-influence hydride ligand; this result is consistent with conclusions of an earlier study of the relationship between agostic bond strengths and the respective *trans* influences of ancillary ligands.^20^

Loss of the agostic interaction in **2′-H(η^2^-Et)** has a barrier Δ*G*^‡^ = 5.5 kcal mol^–1^ (Fig. 2). The product of this ring-opening, **2′-H(Et)**, is 18.9 kcal mol^–1^ lower in free energy than agostic **2′-H(η^2^-Et)**. Since this is formally only a bond breaking reaction, with no concomitant bond making, a negative value of Δ*G*, and particularly such a strongly negative value (–18.9 kcal mol^–1^), is quite striking. This result can be explained, however, in terms of the geometry of reactant and product. In **2′-H(η^2^-Et)**, the strong-*trans*-influence agostic ethyl group α-carbon is positioned *trans* to the strong-*trans*-influence hydride ligand. In contrast, **2′-H(Et)** adopts a so-called Y-geometry,^21^ in which the Cα–Rh–H angle, instead of being *ca.* 180° (mutually *trans*) is only 72°, while the N–Rh–Cα angle (106° in **2′-H(η^2^-Et)**) is 149.0° (Fig. 3). As a result, **2′-H(Et)** has a very short Rh–C bond (2.095 Å *vs.* 2.175 Å in **2′-H(η^2^-Et)**) and a much shorter Rh–H bond (1.546 Å *vs.* 1.640 Å) than is found in **2′-H(η^2^-Et)**.

The barrier to elimination of ethane from **2′-H(Et)** is only Δ*G*^‡^ = 6.0 kcal mol^–1^ as compared with 24.4 kcal mol^–1^ for the reverse reaction, *i.e.* re-formation of the agostic bond to give **2′-H(η^2^-Et)**. Thus the “ring-opening” of **2′-H(η^2^-Et)** is the rate-determining step for the overall loss of ethane from dihydride ethylene complex **2′-H_2_(C_2_H_4_)**. Attempts to locate a TS for rotation around the ethylene C–C bond of agostic complex **2′-H(η^2^-Et)** only led to loss of the agostic interaction to give **2′-H(Et)**. Thus, although insertion of ethylene into a Rh–H bond of **2′-H_2_(C_2_H_4_)** is fully reversible, the calculations predict that it should not lead to exchange between hydride (or deuteride) and ethylene H atoms, in accord with the observed lack of H/D exchange between **2-D_2_** and C_2_H_4_.

The reaction of dihydride **2-H_2_** with TBE, as noted above, does not proceed at room temperature in contrast with the reaction with ethylene, which occurs at –30 °C. The TBE reaction proceeds slowly at 80 °C; the timescale corresponds to a free energy barrier of *ca.* 26–27 kcal mol^–1^, about 8–9 kcal mol^–1^ greater than the reaction with ethylene. The reaction is calculated to proceed *via* a pathway analogous to that for ethylene. An agostic analogue to **2′-H(η^2^-Et)** is calculated to form with a free energy 24.2 kcal mol^–1^ higher than **2′-H_2_** plus TBE, followed by a rate-determining ring-opening with a TS that is 3.3 kcal mol^–1^ higher. The overall barrier for the reaction is thus Δ*G*^‡^ = 27.5 kcal mol^–1^, about 10 kcal mol^–1^ greater than the reaction barrier with ethylene, in very good agreement with experiment. While the Ir analogue was previously shown to react with TBE *via* an Ir(iii)/Ir(v) pathway requiring the presence of H_2_, no acceleration by H_2_ is observed in the present Rh system. This is consistent with the calculated barrier for elimination of neo-hexane from (carb-PNP′)Rh(*t*-butylvinyl)(H), Δ*G*^‡^ = 5.9 kcal mol^–1^, which is far lower than the barrier calculated for the back-reaction (Δ*G*^‡^ = 28.6 kcal mol^–1^).

**Fig. 5:**
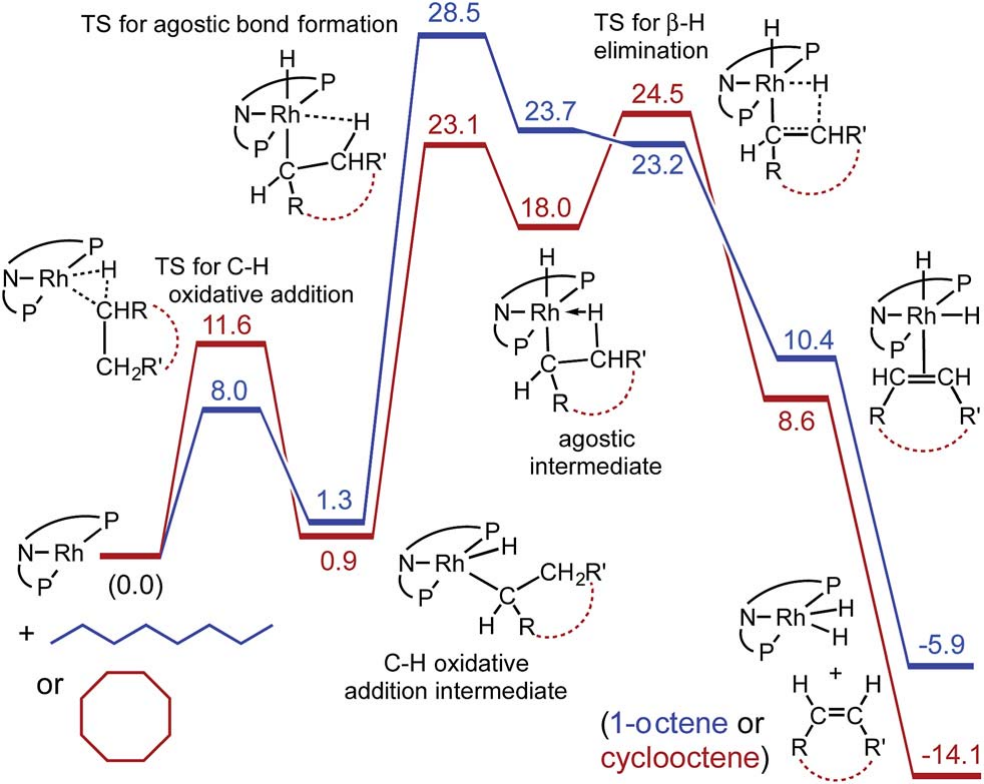
Calculated free energies (kcal mol^–1^) for reaction of **2** with *n*-octane (blue) and with cyclooctane (red) at 200 °C (gas phase, pressures corresponding to molarity of pure liquid).

The calculations also provide insight into the much greater rate of dehydrogenation of COA compared with *n*-octane (free energy values are shown in Fig. 5, expressed relative to **2′** plus the free alkane and calculated for *T* = 473 K in the gas phase with pressures that correspond to the molarity of the respective pure liquid alkanes). Oxidative addition of the C–H bond of COA has a calculated barrier *ca.* 4 kcal mol^–1^ higher than that of *n*-octane. However, the TS for formation of the β-agostic species (carb-PNP′)RhH(η^2^-1-octyl), which is rate-determining for *n*-octane dehydrogenation, is 5.4 kcal mol^–1^ higher than the TS for formation of the corresponding β-agostic cyclooctyl complex. This may be explained in terms of the eclipsed interactions required by the formation of agostic complex (carb-PNP′)RhH(η^2^-1-octyl) (see Fig. 4a for the ethyl analogue). Such unfavorable interactions are also present in the TS for formation of (carb-PNP′)RhH(η^2^-cyclooctyl). However, in the case of COA, unlike *n*-octane, these eclipsed interactions are already present in the alkane substrate (being responsible for the well known ring strain of COA) as well as in the non-agostic C–H addition product. Thus, relative to these free species and the non-agostic alkyl hydride, the TS for agostic bond formation for COA is significantly lower in energy that that for *n*-octane. It may be relevant in this context that unlike the case for *n*-octane or ethane, the agostic cyclooctyl complex (the analogue of **2′-H(η^2^-Et)**) appears to be a distinct minimum on the free energy surface (Fig. 5), and not only a minimum on the electronic energy surface.

On the dehydrogenation pathway the step subsequent to formation of the agostic intermediate is β-H-elimination. In the case of *n*-octane dehydrogenation the TS for this step, TS(**2′-H(η^2^-1-Oc)**/**2′-H(1-Oc)**), has a much lower free energy (23.2 kcal mol^–1^) than the TS for the (rate-determining) formation of the agostic complex (28.5 kcal mol^–1^). In contrast, β-H elimination of the agostic cyclooctyl complex (carb-PNP′)RhH(η^2^-cyclooctyl) is calculated to have a TS slightly higher in free energy (24.5 kcal mol^–1^) than the TS for formation of the agostic complex (23.1 kcal mol^–1^), although this difference is quite small (and probably too small to be meaningful for the comparison of such different species).

Interestingly, the free energy of the TS for β-H elimination of the cyclooctyl complex (24.5 kcal mol^–1^) is higher than that for the 1-octyl complex (23.2 kcal mol^–1^). Likewise (but not surprisingly), as noted above, the TS for C–H addition of COA is of higher free energy than that for *n*-octane. These steps, C–H addition and β-H elimination, are the steps most commonly considered in the context of alkane dehydrogenation (while their microscopic reverse reactions are regarded similarly for olefin hydrogenation). But although the higher reactivity of COA *vs. n*-alkanes is a staple of organometallic-catalyzed alkane dehydrogenation, in the present system the TSs of both of these steps are calculated to be higher in free energy for the dehydrogenation of COA than of *n*-octane. The higher reactivity of COA *vs. n*-octane in the present system, according to our calculations, is a result of only the lower energy of the unanticipated transition state for the formation of an agostic interaction in the case of COA.

## Conclusions

The iridium dihydride complex **1-H_2_** based on the carbazole bis-phosphine ligand was previously reported to be ineffective as a transfer-dehydrogenation catalyst. This was found to be ultimately attributable to the very high energy of the (carb-PNP)Ir(i) complex relative to (carb-PNP)Ir(iii). Thus potential hydrogen acceptors such as TBE inserted into an Ir–H bond (maintaining the Ir(iii) oxidation state), but the barrier to subsequent elimination to give the Ir(i) product was prohibitively high while deinsertion was much more favorable. Hydrogenation by H_2_ was effected, but this was found to proceed *via* an Ir(iii)/Ir(v) pathway involving addition of H_2_ to the Ir(iii) alkyl hydride; such a path is not viable for alkane dehydrogenation.

As the M(i)/M(iii) thermodynamics are biased more towards M(i) in the case of Rh than Ir,^22^ we suspected the relatively high stability of a Rh(iii) analogue would not preclude, and might even favor, transfer dehydrogenation. Indeed the complex **2-H_2_** is found to be an active catalyst for the dehydrogenation of COA with TBE achieving TOFs up to 10 min^–1^, similar to the catalyst (^*t*Bu_4_^PCP)IrH_2_.^7^ To our knowledge this is the first example of a highly active rhodium-based alkane transfer-dehydrogenation catalyst that does not require light or H_2_ atmosphere. However, decomposition of the catalyst at 200 °C limits the catalyst efficiency.

*n*-Octane dehydrogenation proceeded more slowly than COA dehydrogenation. DFT calculations indicate that the slower rate for *n*-octane is attributable to the barrier to a rate-determining step not heretofore given consideration in the context of alkane dehydrogenation (or its microscopic reverse, in the case of alkene hydrogenation), namely the formation of an agostic intermediate, (carb-PNP′)RhH(η^2^-1-octyl), subsequent to C–H addition. Even so the reaction is not prohibitively slow; however, the combination of relatively rapid decomposition at 200 °C and the relatively slow dehydrogenation rate leads to very limited TONs. The development of more stable rhodium pincer complexes based on a similar framework is currently underway.

## Supplementary Material

Supplementary information
