# Supplementary material for: Experimental and computational study of alkane dehydrogenation catalyzed by a carbazolide-based rhodium PNP pincer complex
Source: Chem Sci. 2016 Jan 20;7(4):2579–86. doi: 10.1039/c5sc04794c (PMC5477040; doi:10.1039/c5sc04794c)
Supplement: Supplementary file 1 [file SC-007-C5SC04794C-s001.pdf]

Electronic Supplementary Material (ESI) for Chemical Science.
This journal is © The Royal Society of Chemistry 2016

Supporting Information

Experimental and Computational Study of Alkane Dehydrogenation Catalyzed by a Carbazolide-Based Rhodium PNP Pincer Complex

David Bézier,[†] Changjian Guan,[‡] Karsten Krogh-Jespersen,[‡] Alan S. Goldman*[‡] and Maurice Brookhart*,[†]

[†] Department of Chemistry, The University of North Carolina at Chapel Hill, Chapel Hill, North Carolina 27599, United States.

[‡] Department of Chemistry and Chemical Biology, Rutgers, The State University of New Jersey, New Brunswick, New Jersey 08903, United States.

E-mail: alan.goldman@rutgers.edu; mbrookhart@unc.edu

Contents

1) General experimental details **S2**

2) Experimental procedures and data **S2**

3) Images of NMR spectra for new compounds **S6**

4) References **S13**

5) Computational Details **S14**

6) Assessment of "Phosphine Sterics" Model: ^{tBu2Me2}PNP vs. ^{iPr4}PNP **S15**

7) Structures and energies for computational model species **S16**

1) General experimental details

All manipulations were carried out under an argon atmosphere using standard Schlenk, high-vacuum, and glovebox techniques. Argon was purified by passing through columns of BASF R3-11 catalyst (Chemalog) and 4 Å molecular sieves. Benzene-d_6 and toluene-d_8 (Cambridge Isotope Laboratories) were used without purification. Benzene, toluene, cyclooctane (COA) (99%), *tert*-butylethylene (TBE) (98.5%) and *n*-octane (99%) were distilled from sodium, then degassed by three freeze-pump-thaw cycles and stored in an argon atmosphere glovebox prior to use. 1,8-Bis((diisopropylphosphino)methyl)-3,6-dimethyl-9H-carbazole[S1] and $[Rh(ethylene)_2Cl]_2$[S2] were prepared according to reported procedures. All other reagents and solvents mentioned in this text were purchased from commercial sources and used as received. NMR spectra were recorded on Bruker spectrometers (DRX-400, AVANCE-400, AVANCE-500 and AVANCE-600). ^1H and ^{13}C NMR spectra were referenced to residual solvent peaks. ^{31}P NMR chemical shifts were referenced to an external H_3PO_4 standard. Gas chromatographic analysis of reactions was conducted on an Agilent Technologies 6850 GC instrument fitted with a fused silica capillary column (100 m length × 0.25 mm ID × 0.50 µm film thickness) using the following parameters: FID detector: temperature = 300 ºC, initial temperature: 40 ºC, final temperature: 250 ºC; oven program: 40 ºC, hold for 20 min, ramp 1: 85 ºC/min to 150 ºC, hold for 5 min, ramp 2: 10 ºC/min to 250 ºC, hold for 20 min. Calibration curves were prepared using standard samples. Products were confirmed using authentic samples and calibrated with an internal standard (mesitylene).

2) Experimental procedures and data

a) Synthesis of 1,8-Bis((diisopropylphosphino)methyl)-3,6-dimethylcarbazolide Rhodium(I) Ethylene (2-C_2H_4). To a solution of 1,8-bis((diisopropylphosphino)methyl)-3,6-dimethyl-9H-carbazole (0.200 g, 0.44 mmol) in benzene (15 mL) was added slowly $LiN(TMS)_2$ (0.074 g, 0.44 mmol) at 23 °C, and the solution turned dark brown instantly. After stirring for 5 min, a solution of $[(C_2H_4)_2RhCl]_2$ (0.085 g, 0.22 mmol) in benzene (5 mL) was added to the reaction mixture. After stirring for 10 min, the reaction mixture was filtered, and the volatiles were evaporated to afford a brown solid. The solid was washed 6 times with *n*-octane at -40 °C, dried under vacuum, affording **2-C_2H_4** as a yellow solid (0.19 g, 0.25 mmol, 57%). $^{31}P\{^1H\}$ NMR (C_6D_6, 243 MHz): δ 44.12 (d, $J_{P\text{-}Rh}$ = 130 Hz). ^1H NMR (C_6D_6, 400 MHz): δ 8.07 (s, 2H, Ar-H), 6.96 (s, 2H, Ar-H), 2.89 (s, 4H, CH_2), 2.64 (s, 6H, Ar-CH_3), 2.51 (vt, $J_{P\text{-}H}$ = 5.2 Hz, 4H, C_2H_4), 1.74-1.69 (m, 4H, $C\mathit{H}(CH_3)_2$), 1.04 (q, J = 7.2 Hz, 12H, $CH(C\mathit{H}_3)_2$), 0.95 (q, J = 6.5 Hz, 12H, $CH(C\mathit{H}_3)_2$. ^{13}C NMR (toluene-d_8, 126 MHz): δ 149.58 (vt, J = 3.8 Hz, 2C), 126.33 (vt, J = 3.1 Hz, 2C),125.73 (s, 2C), 124.67 (s, 2C), 120.52 (s, 2C), 118.99 (s, 2C), 36.29 (d, J = 12.7 Hz, 2C), 24.11 (vt, J = 9.6 Hz, 4C), 22.14 (vt, J = 9.1 Hz, 4C), 21.59 (s, 4C), 19.84 (s, 2C), 17.78 (s, 2C). Anal. Calcd for $C_{30}H_{46}NP_2Rh$: C 61.54, H 7.92, N 2.39; found: C 61.76, H 8.04, N 2.19.

b) Synthesis of 1,8-Bis((diisopropylphosphino)methyl)-3,6-dimethylcarbazolide Rhodium(I) carbonyl (2-CO). Carbon monoxide was purged through a solution of **2-C_2H_4** (16 mg, 0.027 mmol) in toluene (1 mL) for 10 min. The solvent was evaporated affording **2-CO** as a yellow solid (15 mg, 0.025 mmol, 94%). $^{31}P\{^1H\}$ NMR (C_6D_6, 243 MHz): δ 54.68 (d, $J_{P\text{-}Rh}$ = 125 Hz). ^1H NMR (C_6D_6, 600 MHz): δ 8.04 (s, 2H, Ar-H), 6.98 (s, 2H, Ar-H), 3.04 (vt, $J_{P\text{-}H}$ = 3.4 Hz, 4H, CH_2), 2.62

(s, 6H, Ar-CH_3), 2.00-1.98 (m, 4H, $CH(CH_3)_2$), 1.15 (q, J = 7.4 Hz, 12H, $CH(CH_3)_2$), 0.94 (q, J = 7.0 Hz, 12H, $CH(CH_3)_2$. ^{13}C NMR (C_6D_6, 151 MHz): δ 194.86 (dt, J = 62 Hz, J = 19 Hz, 1C, CO), 148.63 (vt, J = 4.9 Hz, 2C), 127.08 (vt, J = 3.6 Hz, 2C), 126.49 (s, 2C), 125.15 (s, 2C), 120.46 (s, 2C), 119.46 (s, 2C), 26.08 (vt, J = 12.4 Hz, 4C), 22.04 (vt, J = 9.4 Hz, 4C), 21.63 (s, 4C), 19.28 (s, 2C), 18.39 (s, 2C). Anal. Calcd for $C_{29}H_{42}NOP_2Rh$: C 59.49, H 7.23, N 2.39; found: C 59.13, H 7.59, N 2.07. IR (hexanes, cm^{-1}): 1954 ν(CO).

c) Synthesis of 1,8-Bis((diisopropylphosphino)methyl)-3,6-dimethylcarbazolide Rhodium(I) dihydride (2-H_2). Hydrogen was purged through a solution of **2-C_2H_4** (100 mg, 0.17 mmol) in toluene (5 mL) for 20 min. The solvent was evaporated and the solid was washed 4 times with *n*-octane at -40 °C, dried under vacuum, affording **2-H_2** as a yellow solid (65 mg, 0.12 mmol, 68%). $^{31}P\{^1H\}$ NMR (C_6D_6, 162 MHz): δ 65.70 (d, $J_{P\text{-}Rh}$ = 121 Hz). ^{1}H NMR (C_6D_6, 400 MHz): δ 8.08 (s, 2H, Ar-H), 7.04 (s, 2H, Ar-H), 3.07 (s, 4H, CH_2), 2.63 (s, 6H, Ar-CH_3), 1.71-1.69 (m, 4H, $CH(CH_3)_2$), 1.00 (q, J = 7.0 Hz, 12H, $CH(CH_3)_2$), 0.92 (q, J = 7.4 Hz, 12H, $CH(CH_3)_2$, -19.69 (q, J = 16.0 Hz, 2H, RhH). ^{13}C NMR (toluene-d_8, 126 MHz): δ 148.74 (vt, J = 4.2 Hz, 2C), 127.49 (vt, J = 3.5 Hz, 2C),126.42 (s, 2C), 125.29 (s, 2C), 121.40 (s, 2C), 119.50 (s, 2C), 25.05 (vt, J = 12.7 Hz, 4C), 23.49 (vt, J = 9.3 Hz, 4C), 21.76 (s, 4C), 19.00 (s, 2C), 18.81 (s, 2C). Anal. Calcd for $C_{28}H_{44}NP_2Rh$: C 60.11, H 7.93 N 2.50; found: C 60.43, H 8.33, N 2.19.

d) Observation of 1,8-Bis((diisopropylphosphino)methyl)-3,6-dimethylcarbazolide Rhodium(I) tert-butylethylene (2-TBE). In a J-Young tube, TBE (14μL, 0.102 mmol) was added to a solution of **2-H_2** (4 mg, 6.8 μmol) in toluene-d8 (0.6 mL) and heated at 80 °C for 3 h affording (**2-TBE**) in a 50 % NMR yield determined via the use of an internal standard. $^{31}P\{^1H\}$ NMR (toluene-d_8, 162 MHz): δ = 26.21 (dd, $J_{P\text{-}P}$ = 346 Hz, $J_{P\text{-}Rh}$ = 140 Hz), δ = 13.26 (dd, J_{PP} = 347 Hz, $J_{P\text{-}Rh}$ = 130 Hz).

e) Observation of 1,8-Bis((diisopropylphosphino)methyl)-3,6-dimethylcarbazolide Rhodium(III) Ethylene cis-Dihydride (**2-H_2(C_2H_4)**). Ethylene was added by a gas tight syringe through a solution of **2-H_2** (6.7 mg, 0.011 mmol) in toluene-d^8 (0.5 mL) at -88 °C, and the product **2-H_2(C_2H_4)** formed quantitatively. ^{31}P{1H} NMR (toluene-d_8, 202 MHz): δ 84.23 (bs). ^{1}H NMR (toluene-d_8, 500 MHz): Due to some broad signals, only hydride peaks were assigned: *δ* -11.07 (bs, 1H), -20.22 (bs, 1H).

f) General Procedure for Transfer Dehydrogenation of COA or *n*-octane with TBE Catalyzed by 2-H_2. In an argon filled glovebox, a 4 mL Kontes vial equipped with a Teflon screw-cap and a stir bar was charged with the complex **2-H_2** (3.8 mg, 6.83 μmol, 0.3 mol%) and dissolved in a solution of COA (313 μL, 2.33 mmol) or *n*-octane (378 μL, 2.33 mmol). TBE (300 μL, 2.33 mmol) was added to the solution, the flask was sealed and heated in a preheated oil-bath at 200 °C. At regular intervals, the tube was cooled to room temperature and a sample was analyzed by gas chromatography.

g) Rate of ethylene dissociation from 3e

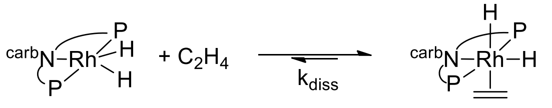

2-H$_2$ **2-H$_2$(C$_2$H$_4$)**

The rate of ethylene dissociation in a temperature range of 185-193 K was estimated via the ^{1}H NMR line broadening of the two hydride signals at δ -20 ppm.

At T = 193 K:
$\Delta\omega = 13\ s^{-1}$
$k_{diss} = 82\ s^{-1}$
$\Delta G^{\ddagger} = 9\pm1$ kcal·mol^{-1}

h) Reaction of (carb-PNP)Rh(H)$_2$ 2-H$_2$ with C_2H_4 to give 2-C_2H_4 and C_2H_6

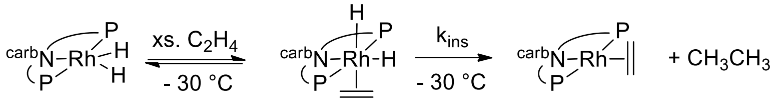

2-H$_2$ **2-H$_2$(C$_2$H$_4$)** **2-C$_2$H$_4$**

2-H$_2$(C$_2$H$_4$) was formed *in situ* via the addition of C_2H_4 by a gas tight syringe to a solution of **2-H$_2$** (4 mg, 7.15 μmol) in toluene d$_8$ at -80 °C. The reactions were monitored by ^{1}H NMR and ^{31}P NMR spectroscopy. The concentration of ethylene in solution was estimated by ^{1}H NMR integration. Due to the equilibrium between **2-H$_2$** and **2-H$_2$(C$_2$H$_4$)** at -30 °C, k_{obs} was corrected by taking in consideration the ratio A = **2-H$_2$(C$_2$H$_4$)** /(**2-H$_2$**+**2-H$_2$(C$_2$H$_4$)**) (determined by ^{31}P NMR). The results are summarized below.

[C_2H_4] (M)	A = **2-H$_2$(C$_2$H$_4$) /(2-H$_2$+2-H$_2$(C$_2$H$_4$))**	k_{obs} (s^{-1})	k_{corr} (corrected rate) (s^{-1}) = k_{obs} (s^{-1}) / A
0.045	0.16	4.20E-05	2.63E-04
0.18	0.42	9.10E-05	2.15E-04
0.25	0.47	1.08E-04	2.29E-04
0.37	0.617	1.30E-04	2.11E-04
0.46	0.625	1.65E-04	2.64E-04

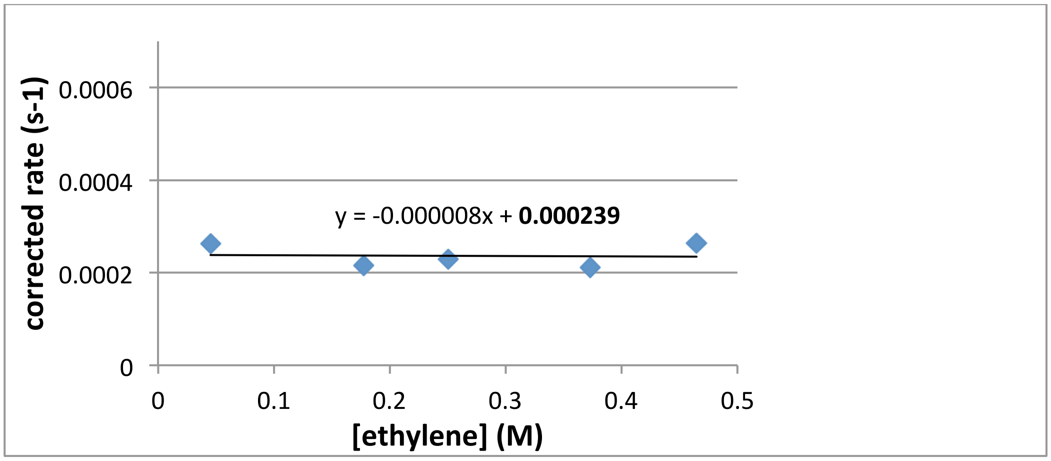

At -30 °C: $k_{corr} = 2.4 \times 10^{-4}\ s^{-1}$; $\Delta G^{\ddagger} = 18\ kcal{\cdot}mol^{-1}$

i) Deuterium labeling experiments

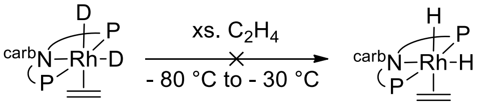

2-D$_2$(C$_2$H$_4$) **2-H$_2$(C$_2$H$_4$)**

2-D$_2$(C$_2$H$_4$) was prepared by purging D_2 through a solution of **2-C$_2$H$_4$** (4.0 mg, 6.8 µmol) in toluene-d^8 (0.6 mL) in a screw-cap NMR tube at room temperature for 10 min, followed by purging with argon for 10 min at rt, and finally C_2H_4 (30 equiv., 0.37 M) was added by gas tight syringe at -80 °C. Starting at -80 °C, the reaction was followed by ^{1}H NMR and warmed up gradually (10 °C every 30 minutes) to -30 °C. No formation of **2-H$_2$(C$_2$H$_4$)** was detected during the course of this reaction.

3) Images of NMR spectra for all new compounds

In C_6D_6

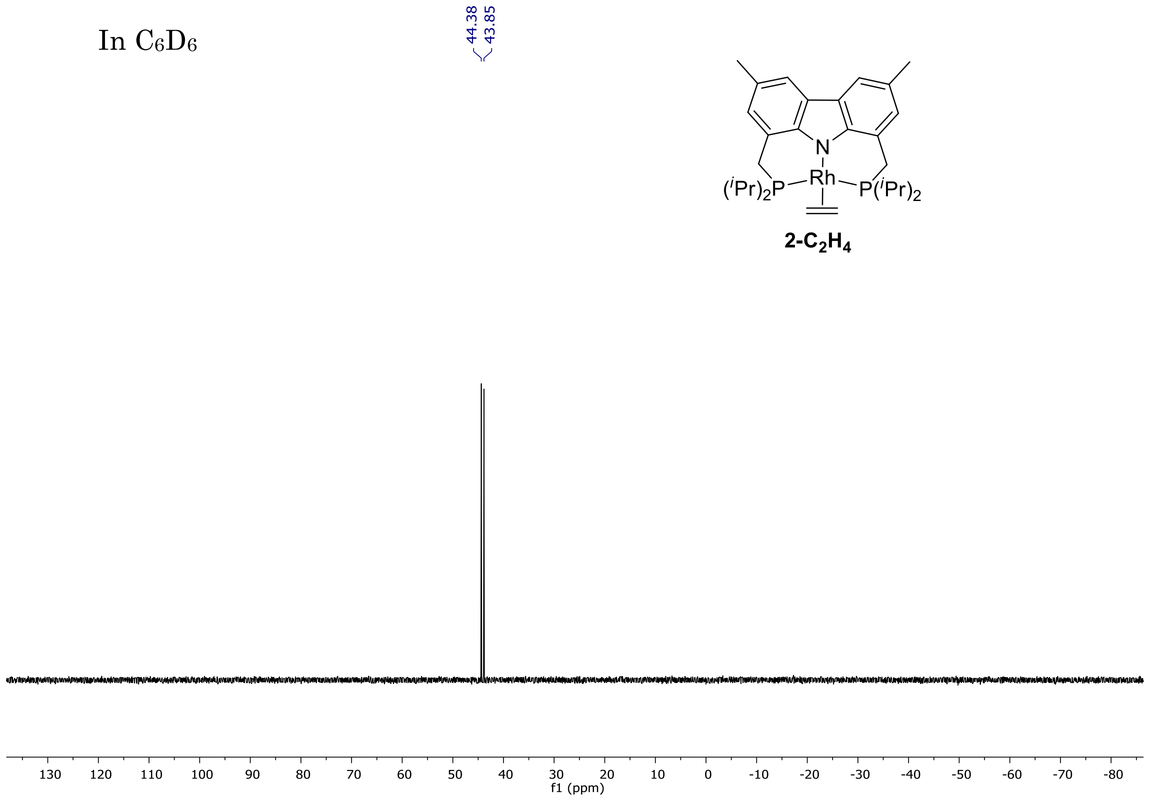

In C_6D_6

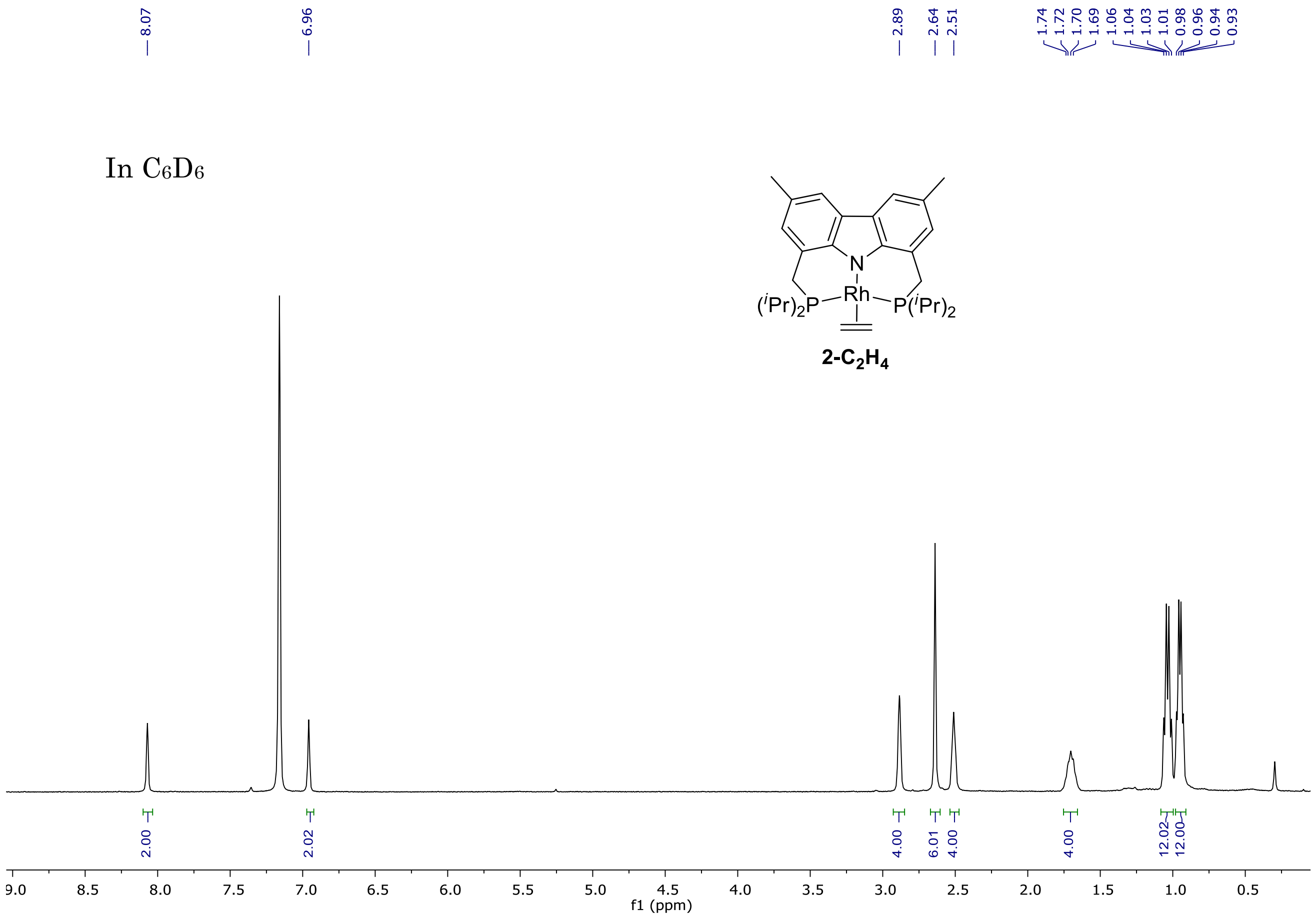

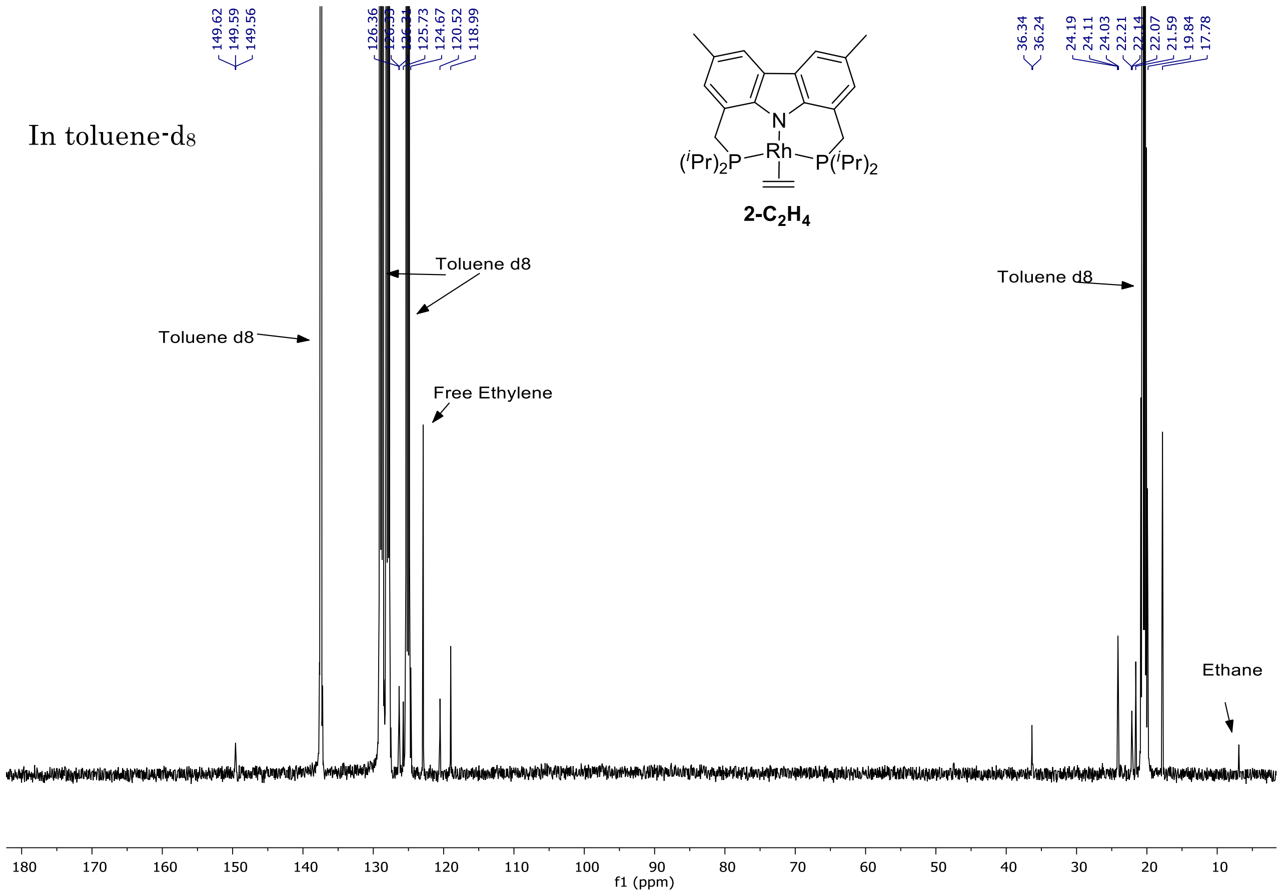
149.62
149.59
149.56
126.36
125.73
124.67
120.52
118.99
36.34
36.24
24.19
24.11
24.03
22.21
22.14
22.07
21.59
19.84
17.78
In toluene-d8
N
Rh
(^{i}Pr)$_2$P
P(^{i}Pr)$_2$
2-C$_2$H$_4$
Toluene d8
Toluene d8
Toluene d8
Toluene d8
Free Ethylene
Ethane
180 170 160 150 140 130 120 110 100 90 80 70 60 50 40 30 20 10
f1 (ppm)

In C_6D_6

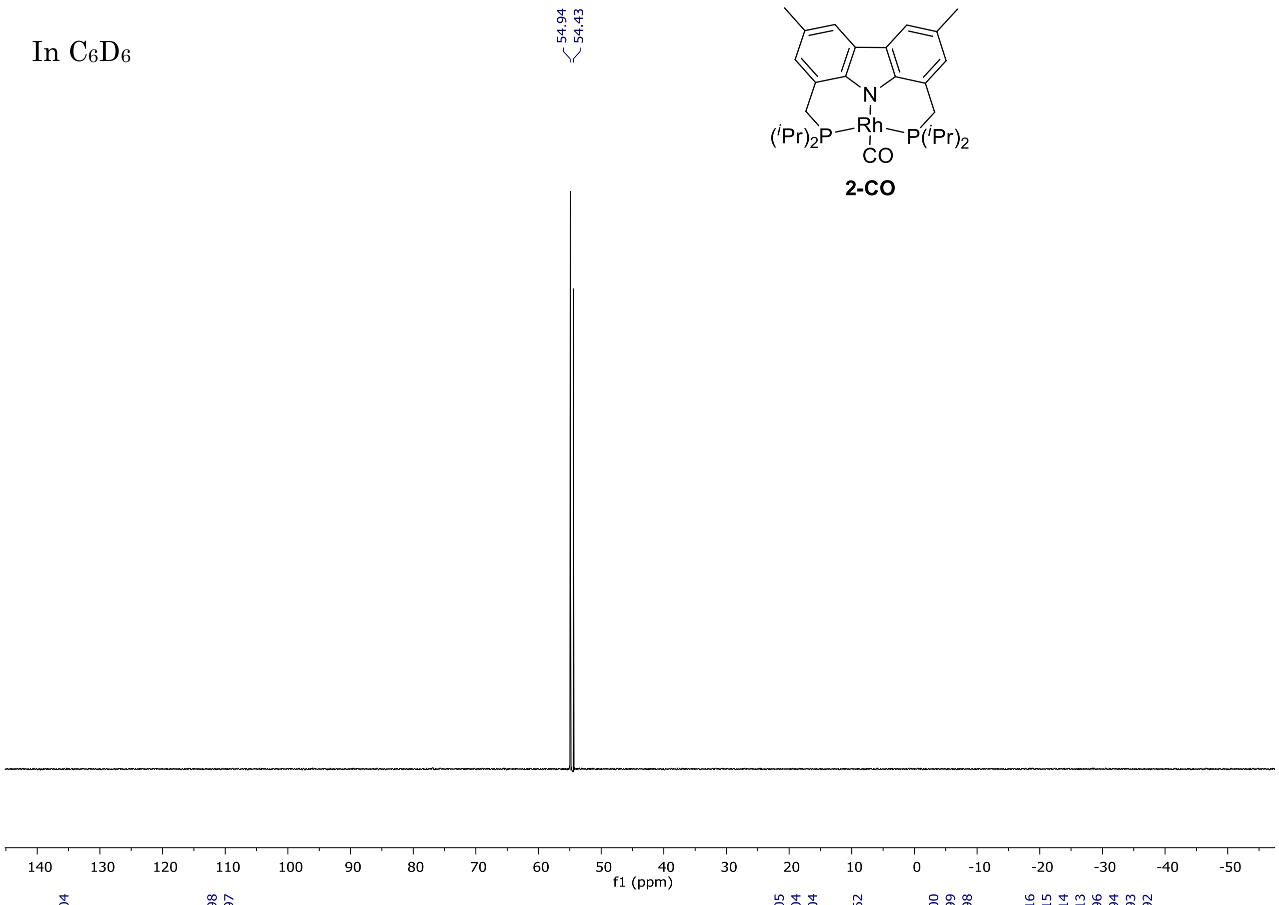

In C_6D_6

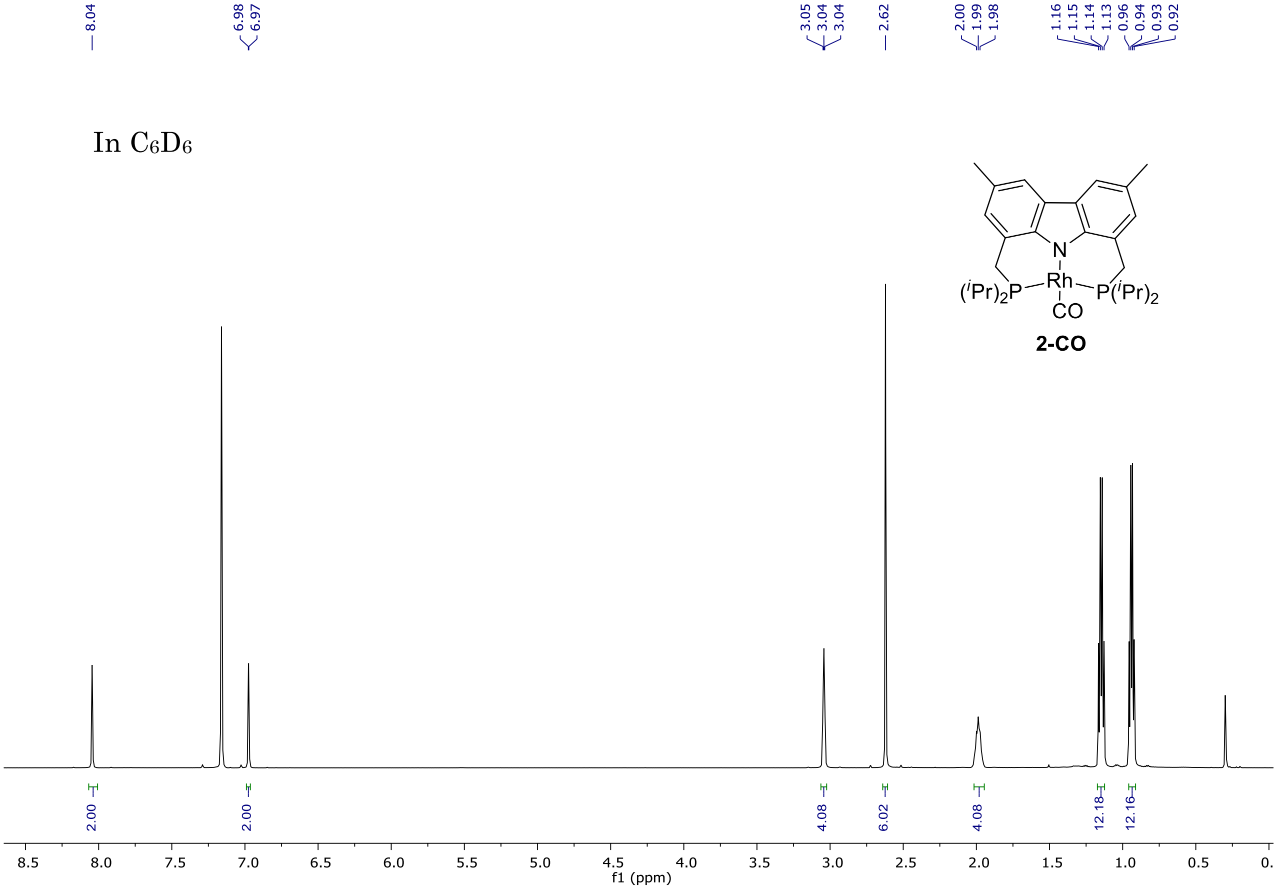

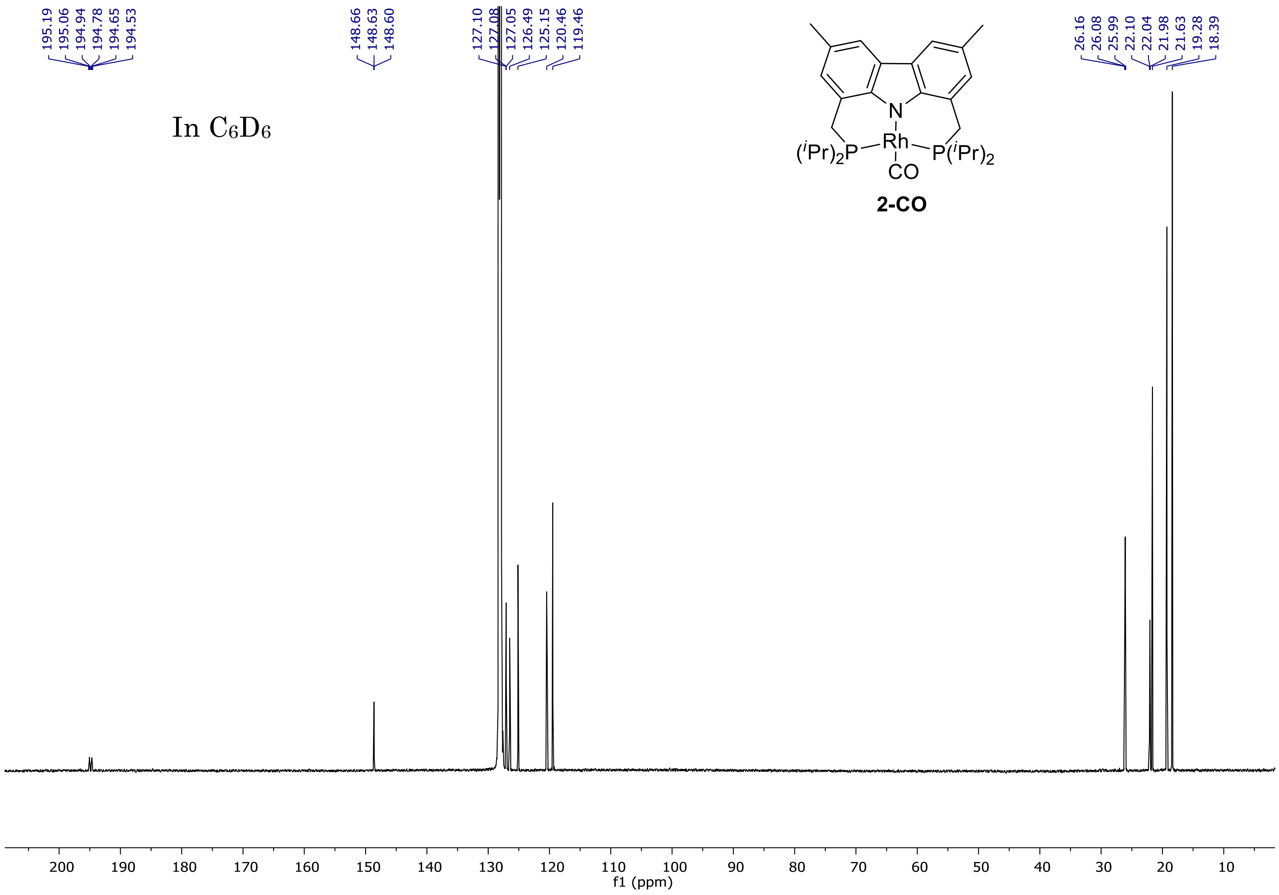
195.19
195.06
194.94
194.78
194.65
194.53
148.66
148.63
148.60
127.10
127.08
127.05
126.49
125.15
120.46
119.46
26.16
26.08
25.99
22.10
22.04
21.98
21.63
19.28
18.39
In C_6D_6
N
Rh
CO
$(^iPr)_2P$
$P(^iPr)_2$
2-CO
200
190
180
170
160
150
140
130
120
110
100
90
80
70
60
50
40
30
20
10
f1 (ppm)

In C_6D_6

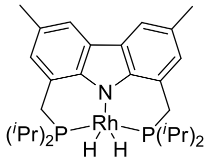

2-H$_2$

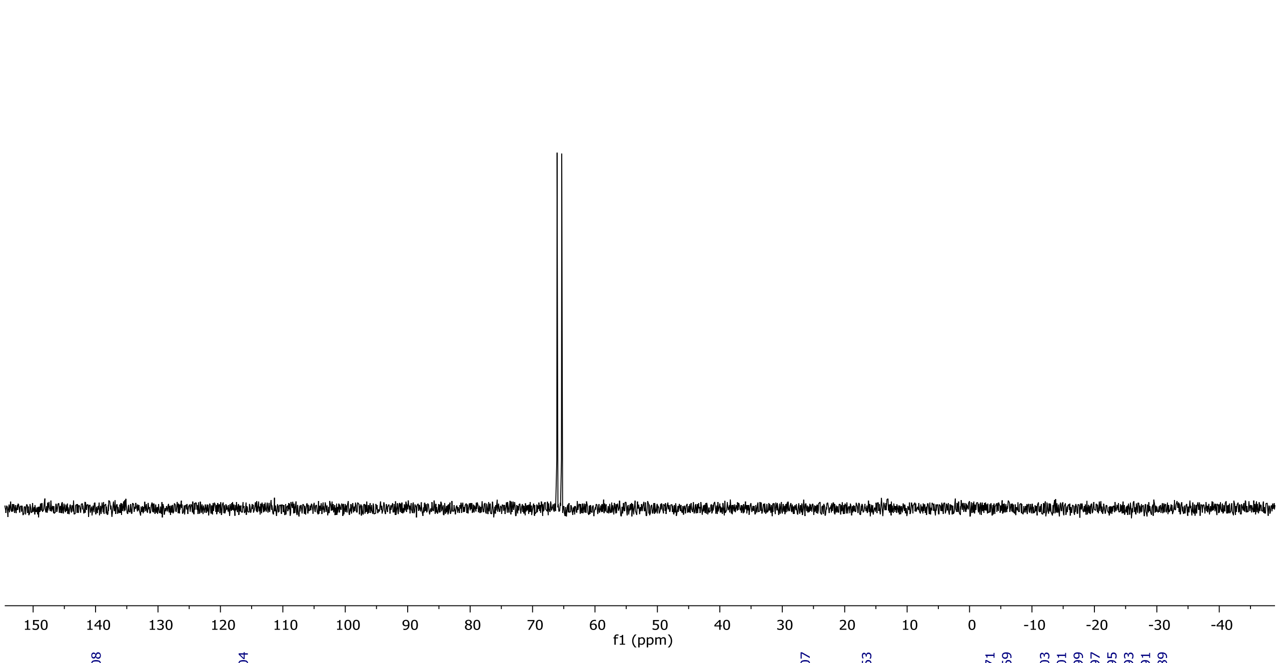

In C_6D_6

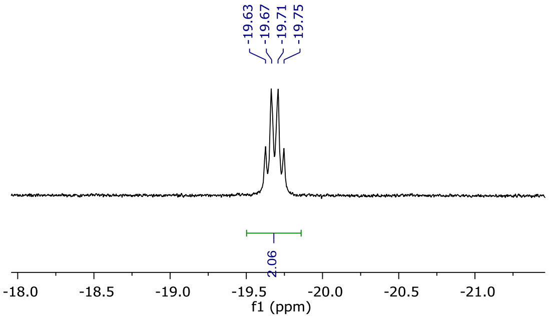

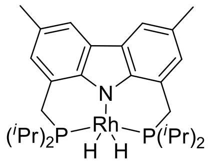

2-H$_2$

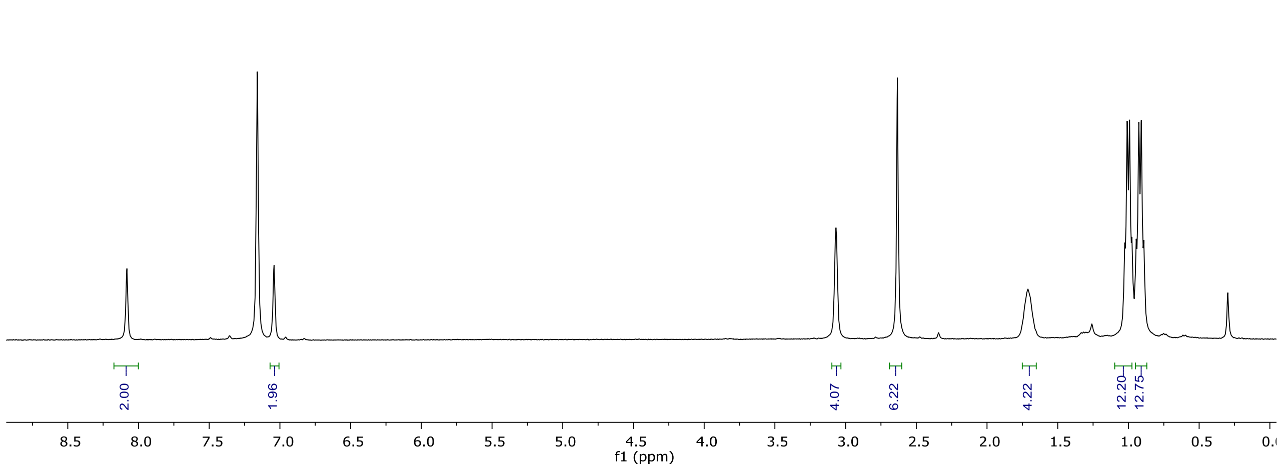

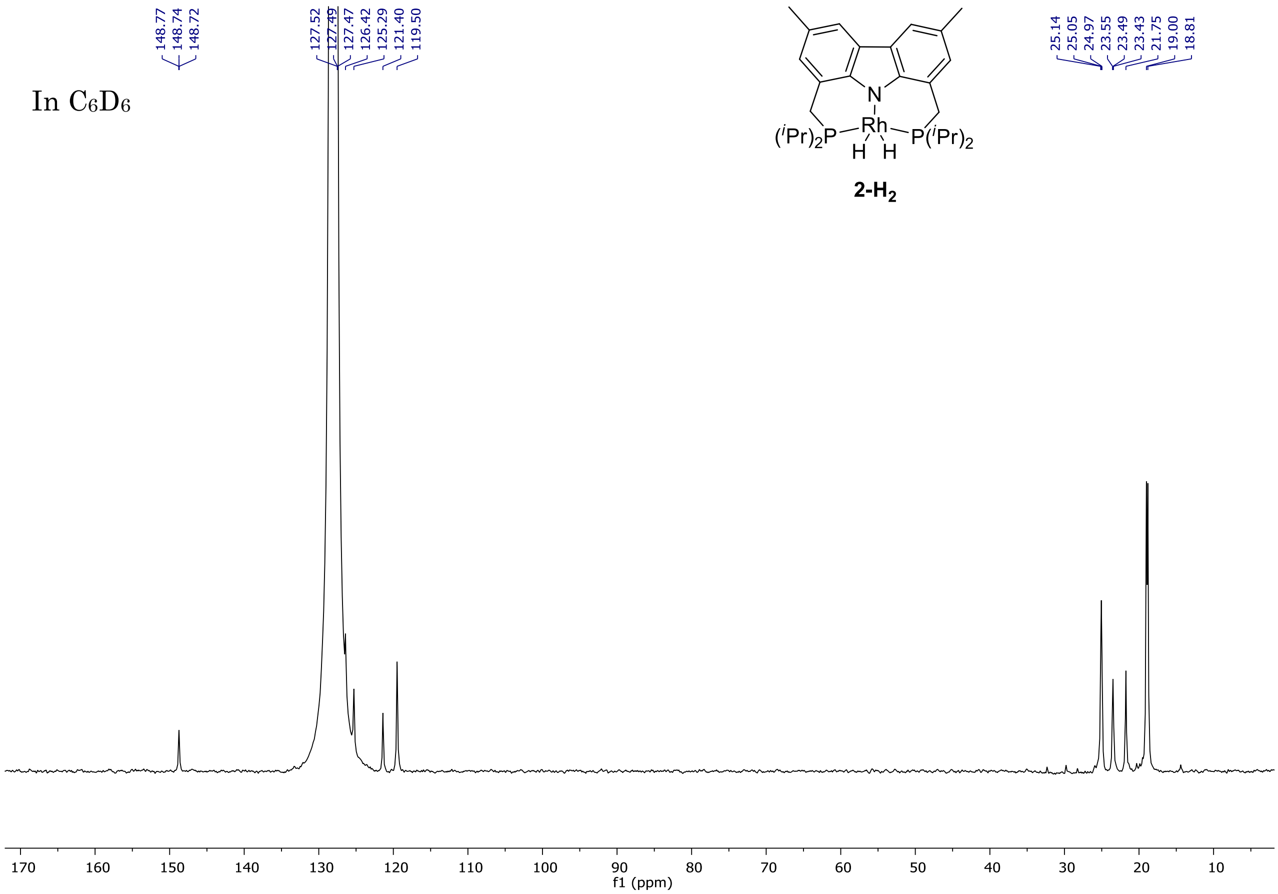
In C_6D_6
148.77
148.74
148.72
127.52
127.49
127.47
126.42
125.29
121.40
119.50
25.14
25.05
24.97
23.55
23.49
23.43
21.75
19.00
18.81
N
Rh
H H
(^{i}Pr)$_2$P
P(^{i}Pr)$_2$
2-H$_2$
170 160 150 140 130 120 110 100 90 80 70 60 50 40 30 20 10
f1 (ppm)

In toluene-d_8

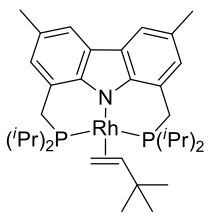

2-TBE

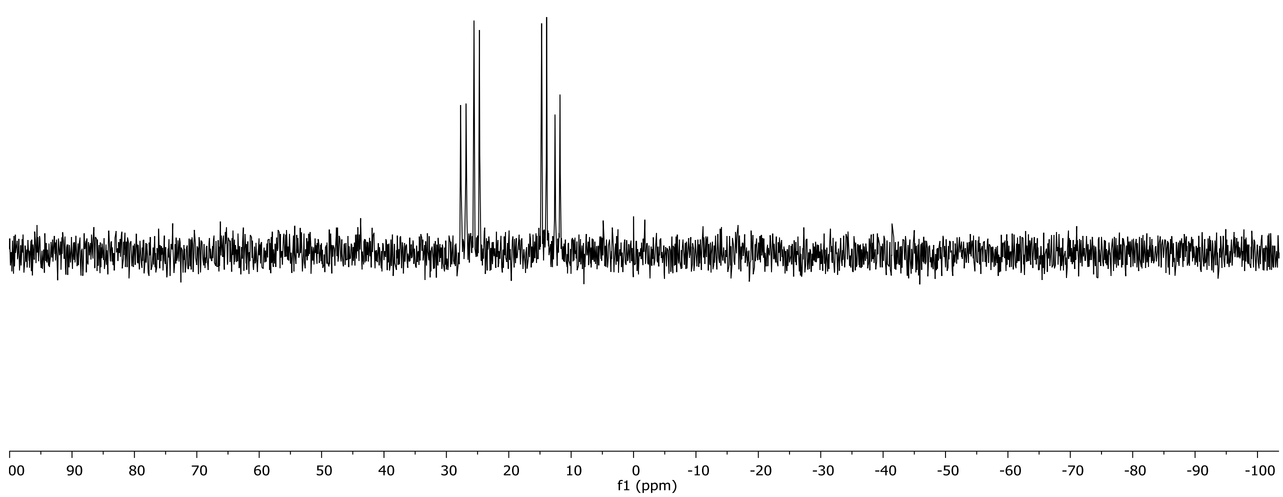

In toluene-d_8 at -88 °C

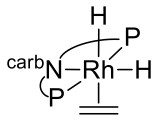

2-$H_2(C_2H_4)$

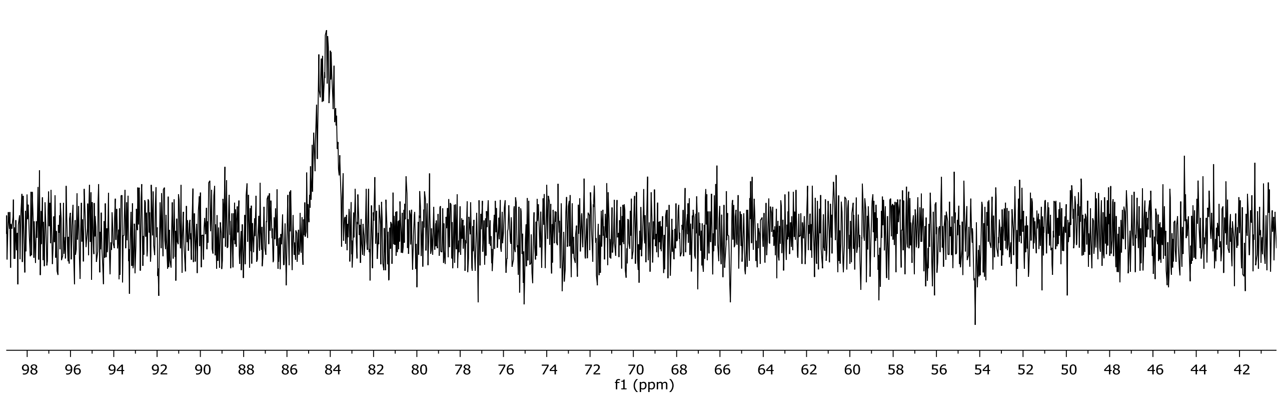

4) References

(S1) Cheng, C.; Kim, B. G.; Guironnet, D.; Brookhart, M.; Guan, C.; Wang, D. Y.; Krogh-Jespersen, K.; Goldman, A. S. *J. Am. Chem. Soc.* 2014, **136**, 6672-6683.

(S2) R. Cramer, J. A. McCleverty and J. Bray, *Inorg. Synth.*, 2007, 14.

5) Computational Details. All electronic structure calculations employed the DFT method[1] and the M06-L[2] functional. The electronic environment was modeled using the following scheme: for Rh, we applied the SDD relativistic effective (small) core potential and the associated (6s5p3d) valence basis set,[3] augmented with an f-type function and a complete set of diffuse spdf functions;[4] all-electron 6-311G(d,p) basis sets were applied to all other atoms.[5] Reactant, transition state and product geometries were fully optimized and characterized by normal mode analysis. Expanded integration grid sizes (pruned (99,590) atomic grids invoked using the integral=ultrafine keyword) were applied to increase numerical accuracy and stability in both geometry optimizations and normal mode analysis.[6] The (unscaled) vibrational frequencies formed the basis for the calculation of vibrational zero-point energy (ZPE) corrections; standard thermodynamic corrections (based on the harmonic oscillator/rigid rotor approximations and ideal gas behavior) were made to convert from purely electronic energies (E) to (standard) enthalpies (H) and Gibbs free energies (G; P = 1 atm, T = 298 K).[7] All calculations were executed using the GAUSSIAN 09 series of computer programs.[8]

1. W. Koch and M. C. Holthausen, *A Chemist's Guide to Density Functional Theory*, Wiley, New York, 2001.
2. Y. Zhao and D. G. Truhlar, *J. Chem. Phys.*, 2006, **125**, 194101/194101-194101/194118.
3. D. Andrae, U. Haeussermann, M. Dolg, H. Stoll and H. Preuss, *Theor. Chim. Acta*, 1990, **77**, 123-141.
4. M. A. Iron, A. C. B. Lucassen, H. Cohen, M. E. van der Boom and J. M. L. Martin, *J. Am. Chem. Soc.*, 2004, **126**, 11699-11710.
5. (a) R. Ditchfield, W. J. Hehre and J. A. Pople, *J. Chem. Phys.* 1971, **54**, 724-728. (b) P. C. Hariharan and J. A. Pople, *Molecular Physics* 1974, **27**, 209-214. (c) K. Raghavachari, J. S. Binkley, R. Seeger and J. A. Pople, *J. Chem. Phys.* 1980, **72**, 650-654. (d) A. D. McLean and G. S. Chandler, *J. Chem. Phys.* 1980, **72**, 5639-5648
6. Æ. Frisch, M. J. Frisch, F. R. Clemente and G. W. Trucks, *Gaussian 09 User's Reference*, 147.
7. D. A. McQuarrie, *Statistical Thermodynamics*, Harper and Row, New York, 1973.
8. M. J. Frisch, G. W. Trucks, H. B. Schlegel, G. E. Scuseria, M. A. Robb, J. R. Cheeseman, G. Scalmani, V. Barone, B. Mennucci, G. A. Petersson, H. Nakatsuji, M. Caricato, X. Li, H. P. Hratchian, A. F. Izmaylov, J. Bloino, G. Zheng, J. L. Sonnenberg, M. Hada, M. Ehara, K. Toyota, R. Fukuda, J. Hasegawa, M. Ishida, T. Nakajima, Y. Honda, O. Kitao, H. Nakai, T. Vreven, J. J. A. Montgomery and J. E. O. Peralta, F.; Bearpark, M.; Heyd, J. J.; Brothers, E.; Kudin, K. N.; Staroverov, V. N.; Kobayashi, R.; Normand, J.; Raghavachari, K.; Rendell, A.; Burant, J. C.; Iyengar, S. S.; Tomasi, J.; Cossi, M.; Rega, N.; Millam, N. J.; Klene, M.; Knox, J. E.; Cross, J. B.; Bakken, V.; Adamo, C.; Jaramillo, J.; Gomperts, R.; Stratmann, R. E.; Yazyev, O.; Austin, A. J.; Cammi, R.; Pomelli, C.; Ochterski, J. W.; Martin, R. L.; Morokuma, K.; Zakrzewski, V. G.; Voth, G. A.; Salvador, P.; Dannenberg, J. J.; Dapprich, S.; Daniels, A. D.; Farkas, Ö.; Foresman, J. B.; Ortiz, J. V.; Cioslowski, J.; Fox, D. J., Gaussian 09, Revision D.01, Gaussian, Inc., Wallingford, CT, 2009.

6) Assessment of "Phosphine Sterics" Model: ^{tBu2Me2}PNP vs. ^{iPr4}PNP

In an attempt to avoid the severe computational problem of multiple conformers of similar energies associated with the use of multiple *i*-Pr groups on the P atoms (viz. ^{iPr4}PNP), we propose here to use the conformationally much simpler C_2 symmetric ^{tBu2Me2}PNP ligand which bears two Me^{t}BuP groups (for reasons explained in the main body of the paper) as our model pincer ligand. We have carried out a few calculations using the methodology outlined above in the Computational Details section to partially assess how well ^{tBu2Me2}PNP mimics ^{iPr4}PNP in (PNP)Rh complexes.

A comparison of the two ligand models involving the lowest-energy conformers for the species (^{R4}PNP)Rh, (^{R4}PNP)RhH_2, and *cis*-(^{R4}PNP)$RhH_2(C_2H_4)$ follows (E = potential energy; H = enthalpy at T = 298 K; G = free energy at T = 298 K and P = 1 atm; all energies in kcal/mol):

	ΔE	ΔH	ΔG
(a) (^{tBu2Me2}PNP)Rh + H_2 → (^{tBu2Me2}PNP)RhH_2	-35.6	-33.7	-24.8
(b) (^{iPr4}PNP)Rh + H_2 → (^{iPr4}PNP)RhH_2	-35.6	-33.6	-24.7
(c) (^{tBu2Me2}PNP)Rh + H_2 +C_2H_4 → *cis*-(^{tBu2Me2}PNP)$RhH_2(C_2H_4)$	-47.6	-43.5	-20.9
(d) (^{iPr4}PNP)Rh + H_2 + C_2H_4 → *cis*-(^{iPr4}PNP)$RhH_2(C_2H_4)$	-49.6	-45.4	-22.3

Examination of the reaction energies obtained for the addition of H_2 to (PNP)Rh, reactions (a) and (b), shows that the purely electronic environments around Rh in (^{tBu2Me2}PNP)Rh and (^{iPr4}PNP)Rh are extremely similar. Furthermore, a comparison of the energies obtained with the two model phosphines reveals a difference in formation energy for the 6-coordinate *cis*-(PNP)$RhH_2(C_2H_4)$ species of 2 kcal/mol or less (cf. reactions (c) and (d)), which is certainly satisfactory.

To illustrate further the importance of phosphine bulk, we collect below the computed formation energies (kcal/mol) of the species *cis*-(PNP)$RhH_2(C_2H_4)$ with varying phosphino alkyl groups. The values calculated for (^{tBu2Me2}PNP)Rh are found to be in much better agreement with those obtained for (^{iPr4}PNP)Rh than values calculated for either (^{Me4}PNP)Rh or (^{tBu4}PNP)Rh.

	ΔE	ΔH	ΔG
(e) (^{Me4}PNP)Rh + H_2 + C_2H_4 → *cis*-(^{Me4}PNP)$RhH_2(C_2H_4)$	-51.3	-47.5	-25.4
(f) (^{iPr4}PNP)Rh + H_2 + C_2H_4 → *cis*-(^{iPr4}PNP)$RhH_2(C_2H_4)$	-49.6	-45.4	-22.3
(g) (^{tBu2Me2}PNP)Rh + H_2 +C_2H_4 → *cis*-(^{tBu2Me2}PNP)$RhH_2(C_2H_4)$	-47.6	-43.5	-20.9
(h) (^{tBu4}PNP)Rh + H_2 + C_2H_4 → *cis*-(^{tBu4}PNP)$RhH_2(C_2H_4)$	-40.8	-35.6	-11.9

7) STRUCTURES AND ENERGIES FOR COMPUTATIONAL MODEL SPECIES

2'

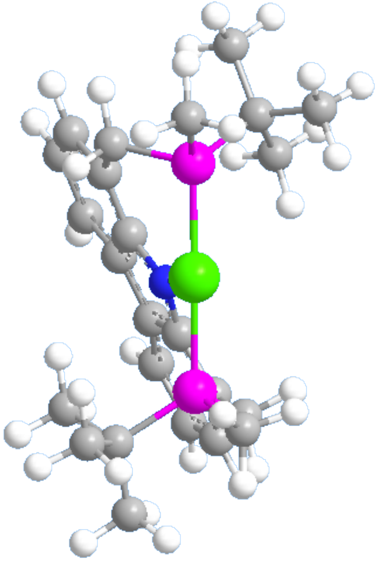

C	-2.54270600	3.56827800	-1.57655800
C	-2.87873400	2.22759100	-1.79773300
C	-2.10305000	1.16422500	-1.34393900
C	-0.93032900	1.47230800	-0.61672700
C	-0.59745800	2.83808100	-0.38916700
C	-1.39593300	3.87662400	-0.86929600
H	-3.18712800	4.35408700	-1.95538600
H	-3.78712100	1.99325000	-2.34810200
H	-1.12088400	4.90939600	-0.67553600
C	0.93038100	1.47222200	0.61676000
C	2.10305700	1.16402700	1.34399900
C	2.87882700	2.22731900	1.79781800
C	2.54292300	3.56803800	1.57664600
C	1.39619000	3.87649200	0.86936700
C	0.59763100	2.83802600	0.38921300
H	3.78718000	1.99289200	2.34820700
H	3.18740700	4.35378600	1.95549500
H	1.12123500	4.90929200	0.67561500
N	-0.00001000	0.64520700	0.00001400
C	2.56768200	-0.23310300	1.63566100
H	1.99106600	-0.67586400	2.46002200
H	3.61803800	-0.22610300	1.94886000
C	-2.56780700	-0.23285600	-1.63562600
H	-3.61819100	-0.22576200	-1.94873200
H	-1.99130400	-0.67560400	-2.46007400
P	2.28093600	-1.36950400	0.23050100
P	-2.28102000	-1.36938800	-0.23058100
C	3.43090500	-0.82481600	-1.15786100
C	3.04840700	-2.91796500	0.85081200

C	-3.04858500	-2.91776100	-0.85099200
C	-3.43089700	-0.82481400	1.15790600
Rh	-0.00004800	-1.37918000	-0.00005700
C	-2.77553800	0.32118100	1.93333900
H	-1.76617000	0.05699100	2.26330200
H	-2.70235800	1.23379300	1.33757700
H	-3.37789200	0.55089600	2.81952800
C	-4.78706700	-0.37523100	0.61903500
H	-5.27786800	-1.14646000	0.01630700
H	-5.45962600	-0.14308800	1.45236500
H	-4.69850600	0.53067500	0.01240000
C	2.77568200	0.32140100	-1.93308500
H	1.76629000	0.05738400	-2.26311400
H	2.70259200	1.23390500	-1.33714600
H	3.37807300	0.55122300	-2.81922000
C	4.78711300	-0.37548400	-0.61887800
H	5.27782300	-1.14688900	-0.01630100
H	5.45970900	-0.14324000	-1.45215000
H	4.69863400	0.53030600	-0.01205700
H	4.12927600	-2.81294300	0.98477300
H	-4.12945800	-2.81268800	-0.98487700
C	-3.60292400	-2.02192800	2.09620600
H	-4.16064800	-1.71464000	2.98708600
H	-4.15840700	-2.84115100	1.63119600
H	-2.63601500	-2.41089300	2.43297200
C	3.60281400	-2.02177200	-2.09638400
H	4.16058000	-1.71437500	-2.98719900
H	4.15820500	-2.84114100	-1.63152400
H	2.63586800	-2.41057100	-2.43323600
H	-2.60413600	-3.16300000	-1.81768000
H	-2.86102400	-3.75025700	-0.17138900
H	2.60388900	-3.16327600	1.81745000
H	2.86085800	-3.75039100	0.17112000

Sum of electronic and thermal Energies= -1782.781081
Sum of electronic and thermal Enthalpies= -1782.780137
Sum of electronic and thermal Free Energies= -1782.870445

2'-H$_2$

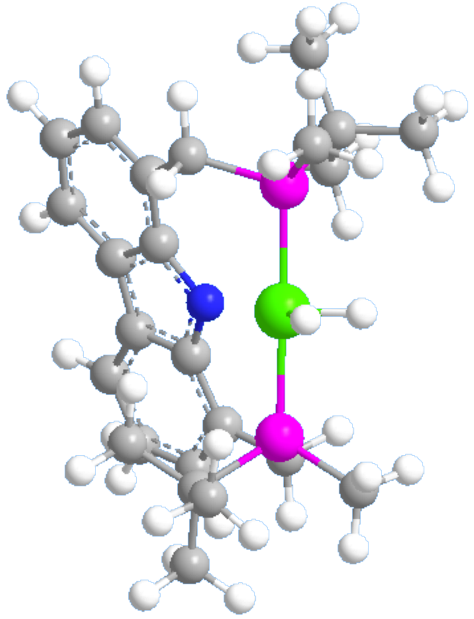

C	-2.65571500	3.69767700	-1.38460900
C	-2.98551900	2.35659100	-1.60918500
C	-2.16681600	1.29817500	-1.22079800
C	-0.96748200	1.61221500	-0.54206600
C	-0.62910000	2.97861600	-0.33864500
C	-1.46485000	4.01433800	-0.75692000
H	-3.33332700	4.47927900	-1.71093800
H	-3.91573500	2.11917400	-2.12062200
H	-1.18444500	5.04929800	-0.58342600
C	0.96748200	1.61230500	0.54200300
C	2.16687400	1.29838800	1.22068900
C	2.98547000	2.35689100	1.60907000
C	2.65551300	3.69794400	1.38453100
C	1.46459800	4.01448400	0.75687300
C	0.62895600	2.97867600	0.33859900
H	3.91573000	2.11956700	2.12046900
H	3.33304800	4.47961400	1.71085500
H	1.18407700	5.04941500	0.58339700
N	0.00003600	0.77150400	-0.00003100
C	2.59261800	-0.09513500	1.58558500
H	2.01585700	-0.45518500	2.45049500
H	3.64555900	-0.10084000	1.88992400
C	-2.59238000	-0.09540100	-1.58568300
H	-3.64526200	-0.10121200	-1.89022600
H	-2.01541200	-0.45548600	-2.45044000
P	2.27601300	-1.35572300	0.29870500
P	-2.27591300	-1.35583400	-0.29860700

C	3.38461600	-0.94890000	-1.16313200
C	3.03180500	-2.84636500	1.03917500
C	-3.03157400	-2.84658900	-1.03898400
C	-3.38470300	-0.94884900	1.16303900
Rh	0.00005000	-1.37096400	0.00010900
C	-2.70947800	0.12732100	2.01655200
H	-1.70812300	-0.18091000	2.33230100
H	-2.61063500	1.07574000	1.48352400
H	-3.31041300	0.30943400	2.91453600
C	-4.75368400	-0.45684700	0.69715100
H	-5.25634400	-1.17264900	0.03857200
H	-5.40689800	-0.30428300	1.56338700
H	-4.68019500	0.50022800	0.17279600
C	2.70925000	0.12711700	-2.01672300
H	1.70788500	-0.18121600	-2.33234100
H	2.61039400	1.07559900	-1.48381100
H	3.31009400	0.30915000	-2.91478400
C	4.75363200	-0.45676900	-0.69748100
H	5.25639900	-1.17244400	-0.03884700
H	5.40673800	-0.30431900	-1.56382000
H	4.68017200	0.50038900	-0.17327300
H	4.11446100	-2.74917000	1.15962200
H	-4.11422100	-2.74944400	-1.15955900
C	-3.53224200	-2.22577800	1.99402500
H	-4.06338000	-1.99958300	2.92446700
H	-4.10358600	-2.99881800	1.47267300
H	-2.55764800	-2.64492300	2.26521900
C	3.53211700	-2.22594200	-1.99395000
H	4.06313400	-1.99985700	-2.92448800
H	4.10355900	-2.99888000	-1.47255400
H	2.55750900	-2.64517100	-2.26496500
H	-2.58360900	-3.00685300	-2.02160600
H	-2.81652200	-3.72398900	-0.42777700
H	2.58395200	-3.00653300	2.02186400
H	2.81671600	-3.72384300	0.42809300
H	0.12033100	-2.78508400	-0.65722900
H	-0.12020200	-2.78498000	0.65767500

Sum of electronic and thermal Energies= -1783.993521
Sum of electronic and thermal Enthalpies= -1783.992577
Sum of electronic and thermal Free Energies= -1784.083966

TS-2'-H$_2$-2'-H$_2$(C$_2$H$_4$)

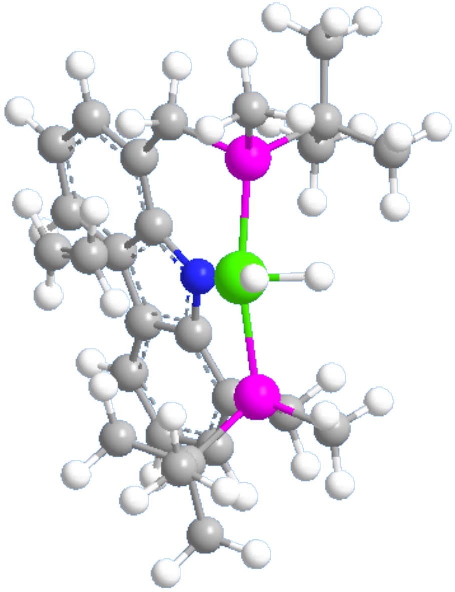

C	2.41770600	4.05316200	1.02293500
C	2.94306800	2.76558200	1.16016000
C	2.26507600	1.61383100	0.75890800
C	0.98946800	1.78010800	0.17196100
C	0.46491200	3.10138100	0.02688000
C	1.16788200	4.22573300	0.45358400
H	2.99602000	4.90787400	1.35726900
H	3.92818400	2.64497400	1.60593000
H	0.74188500	5.21780500	0.33351200
C	-0.97797500	1.55860700	-0.81294200
C	-2.13041200	1.09117600	-1.48224100
C	-3.08353300	2.03309100	-1.86050100
C	-2.92936700	3.40452400	-1.61990000
C	-1.78487100	3.87188100	-0.99714600
C	-0.81029700	2.95401000	-0.60241200
H	-3.97579200	1.68210900	-2.37480900
H	-3.70366100	4.09459200	-1.93766700
H	-1.64286100	4.93476500	-0.82286700
N	0.09523300	0.83775200	-0.31549900
C	-2.35904400	-0.35672300	-1.79577200
H	-1.61327100	-0.72601100	-2.51390700
H	-3.34425300	-0.49944200	-2.25357000
C	2.95967400	0.30414700	1.04707900
H	4.04014600	0.42096000	0.89777600
H	2.83944300	0.05797900	2.11185700
P	-2.14727600	-1.45229500	-0.33621000
P	2.38878200	-1.20774300	0.19068100
C	-3.53547100	-1.02164400	0.85960800

C	-2.64667600	-3.07934000	-1.00566500
C	3.27225900	-2.50586700	1.12831100
C	3.23607300	-1.20008200	-1.48889700
C	0.24286800	-0.54076000	3.21088500
H	1.08276800	-1.12006100	3.58523700
C	0.28107100	0.78665200	3.13651500
H	1.15630000	1.35767000	3.43173800
H	-0.63278400	-1.11802800	2.92458300
H	-0.55172400	1.37077800	2.75520100
H	0.06554200	-2.70559300	0.83813400
H	0.24038800	-2.40083000	-0.86865100
C	2.80339900	0.06031900	-2.23885500
H	3.21559400	0.04156800	-3.25365400
H	1.71456100	0.13105800	-2.31164300
H	3.16129600	0.97204500	-1.75256700
C	4.75802900	-1.22326700	-1.34279400
H	5.13673400	-0.38629300	-0.74947900
H	5.11868200	-2.15257500	-0.89374800
H	5.21868300	-1.14433200	-2.33332700
C	-3.15811600	0.23859300	1.63961900
H	-2.21104400	0.11085200	2.16790900
H	-3.06811700	1.11586300	0.99501900
H	-3.92953900	0.45040900	2.38841400
C	-4.85361000	-0.78672400	0.12408200
H	-5.14643300	-1.63437400	-0.50294500
H	-5.65847000	-0.63274900	0.85148200
H	-4.80853400	0.10773400	-0.50341800
H	-3.70382300	-3.11087700	-1.28241100
H	4.33121000	-2.27339800	1.26637400
Rh	0.09852200	-1.28255800	0.16719400
C	2.78816900	-2.43544900	-2.27101000
H	1.71489100	-2.41177000	-2.47333300
H	3.31094600	-2.47028600	-3.23289800
H	3.01193800	-3.36895200	-1.74493200
C	-3.67191000	-2.19501600	1.83303200
H	-4.37007700	-1.93224300	2.63463600
H	-4.05731600	-3.09487800	1.34588600
H	-2.71410100	-2.44937100	2.29987600
H	2.80124100	-2.60869400	2.10728800
H	3.17889100	-3.46587000	0.61717700
H	-2.43939400	-3.86595200	-0.27924600
H	-2.04709400	-3.28257500	-1.89508000

Sum of electronic and thermal Energies= -1862.536791
Sum of electronic and thermal Enthalpies= -1862.535847
Sum of electronic and thermal Free Energies= -1862.634363

****** 1 imaginary frequencies (negative Signs) ******

Diagonal vibrational polarizability:
30.5052865 30.7161164 55.7953012

Harmonic frequencies (cm**-1), IR intensities (KM/Mole), Raman scattering activities (A**4/AMU), depolarization ratios for plane and unpolarized incident light, reduced masses (AMU), force constants (mDyne/A), and normal coordinates:

	1	2	3
	A	A	A
Frequencies --	-82.0464	26.7919	30.4092
Red. masses --	2.7517	2.6107	4.1564
Frc consts --	0.0109	0.0011	0.0023
IR Inten --	5.5767	0.0550	0.2500

2'-$H_2(C_2H_4)$

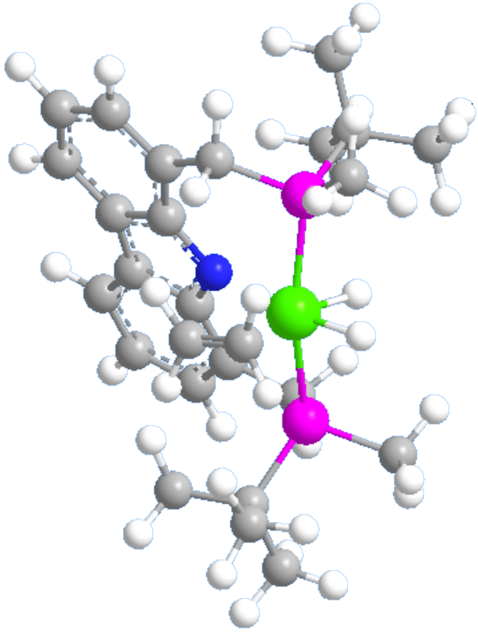

C	-3.75800000	2.70680000	-1.92300000
C	-3.95130000	1.32410000	-2.01440000
C	-3.00940000	0.39280000	-1.57970000
C	-1.81160000	0.89070000	-1.00880000
C	-1.63360000	2.30470000	-0.89680000
C	-2.59440000	3.20210000	-1.36010000
H	-4.52880000	3.38100000	-2.28070000
H	-4.87920000	0.94870000	-2.44070000
H	-2.43560000	4.27260000	-1.26450000
C	0.10400000	1.17730000	0.05030000
C	1.30850000	1.03040000	0.77610000
C	1.98780000	2.19240000	1.13410000
C	1.51810000	3.47650000	0.83210000
C	0.31890000	3.62900000	0.15890000
C	-0.38640000	2.48900000	-0.22670000
H	2.92010000	2.08840000	1.68590000
H	2.09160000	4.34390000	1.14110000
H	-0.07090000	4.61810000	-0.06560000
N	-0.74810000	0.21600000	-0.44830000
C	1.86990000	-0.28680000	1.23480000
H	1.26870000	-0.69430000	2.05910000
H	2.87890000	-0.13600000	1.63670000
C	-3.35060000	-1.06700000	-1.74800000
H	-4.43880000	-1.19330000	-1.78320000
H	-2.98350000	-1.45520000	-2.71050000
P	1.88910000	-1.65750000	0.00920000

P	-2.62740000	-2.20190000	-0.50790000
C	3.33850000	-1.28880000	-1.13850000
C	2.51110000	-3.03870000	1.03560000
C	-3.32550000	-3.81440000	-1.01750000
C	-3.48380000	-1.85100000	1.13060000
Rh	-0.34550000	-1.95800000	-0.59800000
C	-2.89290000	-0.61070000	1.80260000
H	-1.81590000	-0.70380000	1.95880000
H	-3.06710000	0.29680000	1.22070000
H	-3.37110000	-0.47880000	2.77960000
C	-4.98130000	-1.62820000	0.90960000
H	-5.46580000	-2.45970000	0.38890000
H	-5.47920000	-1.52470000	1.87950000
H	-5.17390000	-0.70950000	0.34860000
C	3.17820000	0.11750000	-1.72090000
H	2.17430000	0.30690000	-2.11010000
H	3.37560000	0.88990000	-0.97440000
H	3.88880000	0.25720000	-2.54320000
C	4.67420000	-1.36850000	-0.39720000
H	4.91320000	-2.38570000	-0.07720000
H	5.47920000	-1.04150000	-1.06460000
H	4.70350000	-0.71700000	0.48130000
H	3.40620000	-2.76490000	1.59900000
H	-4.41070000	-3.85510000	-0.89250000
C	-3.25900000	-3.06760000	2.03200000
H	-3.64730000	-2.85620000	3.03350000
H	-3.77370000	-3.95980000	1.66500000
H	-2.19470000	-3.30220000	2.13260000
C	3.32740000	-2.33020000	-2.25970000
H	4.21030000	-2.20190000	-2.89530000
H	3.35080000	-3.35480000	-1.87410000
H	2.44820000	-2.23380000	-2.89970000
H	-3.09780000	-3.98670000	-2.07110000
H	-2.86560000	-4.61810000	-0.44160000
H	1.72240000	-3.32580000	1.73180000
H	2.73200000	-3.90700000	0.41150000
H	-0.12400000	-3.51500000	-0.44160000
H	-0.43350000	-2.09420000	0.96950000
C	-0.16320000	-1.36970000	-2.85130000
H	0.80850000	-0.90360000	-2.95270000
C	-0.29670000	-2.72940000	-2.78330000
H	0.55940000	-3.39110000	-2.84390000
H	-1.00490000	-0.71200000	-3.03350000
H	-1.25510000	-3.20840000	-2.95350000

Sum of electronic and thermal Energies= -1862.547490

```
Sum of electronic and thermal Enthalpies=          -1862.546545
Sum of electronic and thermal Free Energies=       -1862.641320
```

TS-2’-$H_2(C_2H_4)$- 2'-H(η^2-Et)

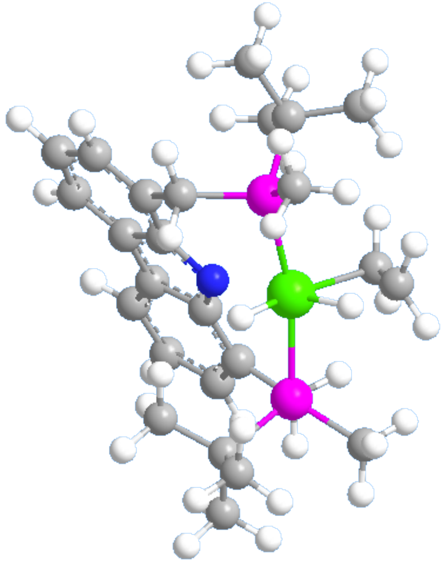

C	-2.98710000	2.91120000	-2.05560000
C	-3.34110000	1.55910000	-2.11010000
C	-2.53760000	0.53890000	-1.60500000
C	-1.30910000	0.90420000	-1.00460000
C	-0.97930000	2.29010000	-0.91290000
C	-1.80150000	3.28110000	-1.44740000
H	-3.65420000	3.65940000	-2.47000000
H	-4.29130000	1.28010000	-2.56040000
H	-1.52000000	4.32720000	-1.36560000
C	0.56310000	1.01450000	0.17550000
C	1.71080000	0.77730000	0.96880000
C	2.48780000	1.87500000	1.33100000
C	2.17000000	3.18930000	0.96860000
C	1.03260000	3.43450000	0.22160000
C	0.23400000	2.35860000	-0.16730000
H	3.37560000	1.69220000	1.93280000
H	2.81240000	4.00470000	1.28300000
H	0.76040000	4.44720000	-0.06210000
N	-0.35350000	0.13230000	-0.36820000
C	2.14570000	-0.58150000	1.43420000
H	1.49100000	-0.96470000	2.22540000
H	3.15910000	-0.52990000	1.84720000
C	-3.05650000	-0.86790000	-1.71820000
H	-4.15040000	-0.85900000	-1.78420000
H	-2.70560000	-1.34190000	-2.64390000
P	2.05210000	-1.84550000	0.11580000
P	-2.51410000	-2.00560000	-0.39430000
Rh	-0.21110000	-2.02580000	-0.39820000
C	-0.09010000	-2.50130000	-2.54850000

H	0.79860000	-2.11800000	-3.03770000
C	0.01160000	-3.72830000	-1.80910000
H	0.97770000	-4.23420000	-1.79080000
H	-0.80320000	-4.44720000	-1.90100000
H	-1.00120000	-2.32230000	-3.11230000
H	-0.30720000	-2.03180000	1.22310000
H	-0.12930000	-3.66520000	-0.39220000
C	2.75700000	-3.32140000	0.93390000
H	2.67170000	-4.20130000	0.29410000
H	3.80610000	-3.18460000	1.20910000
H	2.17610000	-3.51010000	1.83870000
C	-3.33150000	-3.56210000	-0.90400000
H	-3.00620000	-4.39190000	-0.27450000
H	-4.42110000	-3.48910000	-0.85990000
H	-3.05060000	-3.78510000	-1.93610000
C	3.35290000	-1.35000000	-1.15480000
C	2.82160000	-0.19300000	-2.00890000
H	1.80540000	-0.37300000	-2.36860000
H	2.80780000	0.74700000	-1.45400000
H	3.47380000	-0.05540000	-2.87860000
C	3.61580000	-2.56800000	-2.04470000
H	4.32750000	-2.30030000	-2.83290000
H	4.04900000	-3.40450000	-1.48840000
H	2.70660000	-2.92140000	-2.53910000
C	4.65490000	-0.91720000	-0.48040000
H	5.40970000	-0.70560000	-1.24610000
H	4.52190000	-0.00120000	0.10150000
H	5.07110000	-1.68810000	0.17540000
C	-3.41590000	-1.50340000	1.17580000
C	-2.74000000	-0.28540000	1.80850000
H	-1.68140000	-0.46910000	2.00610000
H	-2.81550000	0.60290000	1.17660000
H	-3.23510000	-0.06100000	2.75970000
C	-3.33500000	-2.68500000	2.14470000
H	-2.29750000	-2.99060000	2.31200000
H	-3.75610000	-2.39360000	3.11230000
H	-3.90010000	-3.55300000	1.79230000
C	-4.87670000	-1.16530000	0.87310000
H	-5.40970000	-1.97940000	0.37220000
H	-5.40500000	-0.96740000	1.81180000
H	-4.96430000	-0.26510000	0.25850000

Sum of electronic and thermal Energies= -1862.532967
Sum of electronic and thermal Enthalpies= -1862.532023
Sum of electronic and thermal Free Energies= -1862.625879

****** 1 imaginary frequencies (negative Signs) ******

Diagonal vibrational polarizability:
32.6180746 28.0219513 30.5492250

Harmonic frequencies (cm**-1), IR intensities (KM/Mole), Raman scattering activities (A**4/AMU), depolarization ratios for plane and unpolarized incident light, reduced masses (AMU), force constants (mDyne/A), and normal coordinates:

	1	2	3
	A	A	A
Frequencies --	-659.8252	33.3193	34.6080
Red. masses --	1.2142	3.7997	4.3054
Frc consts --	0.3114	0.0025	0.0030
IR Inten --	4.4208	0.0526	0.4263

2'-H(η^2-Et)

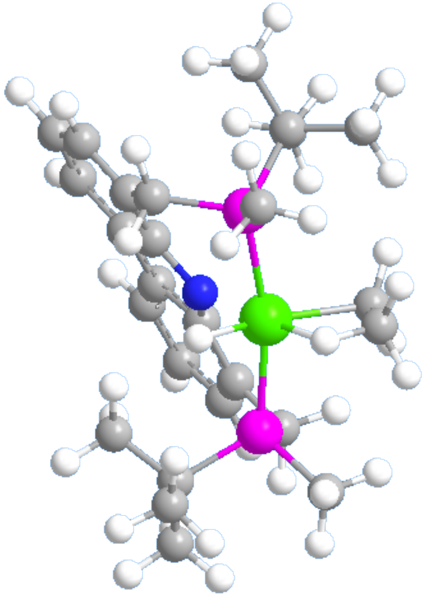

C	-2.95630000	2.94830000	-2.11430000
C	-3.30940000	1.59600000	-2.16660000
C	-2.51200000	0.57790000	-1.64780000
C	-1.28960000	0.94540000	-1.03720000
C	-0.96170000	2.33110000	-0.94680000
C	-1.77820000	3.32020000	-1.49360000
H	-3.61840000	3.69480000	-2.53950000
H	-4.25460000	1.31450000	-2.62550000
H	-1.49710000	4.36630000	-1.41130000
C	0.57590000	1.06590000	0.16360000
C	1.72320000	0.83870000	0.96010000
C	2.49130000	1.94280000	1.32150000
C	2.16690000	3.25390000	0.95470000
C	1.03260000	3.48850000	0.20010000
C	0.24310000	2.40620000	-0.18880000
H	3.37910000	1.76650000	1.92510000
H	2.80280000	4.07440000	1.26920000
H	0.75590000	4.49780000	-0.09130000
N	-0.33690000	0.18060000	-0.38470000
C	2.17240000	-0.51620000	1.42220000
H	1.52700000	-0.90630000	2.21720000
H	3.18980000	-0.45760000	1.82420000
C	-3.03230000	-0.82840000	-1.75210000
H	-4.12520000	-0.81890000	-1.83250000
H	-2.66550000	-1.31660000	-2.66400000
P	2.06840000	-1.77010000	0.09720000
P	-2.49700000	-1.93470000	-0.40130000
Rh	-0.19680000	-1.94190000	-0.40200000

C	-0.08290000	-2.50530000	-2.49930000
H	0.79860000	-2.18290000	-3.04680000
C	0.03350000	-3.76680000	-1.75550000
H	1.00570000	-4.26380000	-1.80340000
H	-0.76310000	-4.49780000	-1.91810000
H	-0.98540000	-2.38870000	-3.09690000
H	-0.28500000	-1.98730000	1.23450000
H	-0.08610000	-3.68330000	-0.53260000
C	2.77570000	-3.25220000	0.90330000
H	2.68500000	-4.13040000	0.26190000
H	3.82810000	-3.11660000	1.16740000
H	2.20460000	-3.44170000	1.81400000
C	-3.29730000	-3.50840000	-0.88480000
H	-2.96930000	-4.32410000	-0.23860000
H	-4.38770000	-3.44260000	-0.84610000
H	-3.00980000	-3.74710000	-1.91150000
C	3.36020000	-1.27300000	-1.18120000
C	2.80380000	-0.14250000	-2.05400000
H	1.79780000	-0.36060000	-2.42160000
H	2.75590000	0.80320000	-1.51040000
H	3.46080000	0.00520000	-2.91850000
C	3.64290000	-2.50120000	-2.05080000
H	4.33470000	-2.23050000	-2.85560000
H	4.10690000	-3.31420000	-1.48470000
H	2.73560000	-2.88840000	-2.52300000
C	4.65470000	-0.80580000	-0.51590000
H	5.40790000	-0.60000000	-1.28490000
H	4.50680000	0.12040000	0.04610000
H	5.08080000	-1.55610000	0.15750000
C	-3.41350000	-1.40820000	1.15230000
C	-2.73540000	-0.18760000	1.77740000
H	-1.67950000	-0.37780000	1.98410000
H	-2.80120000	0.69490000	1.13640000
H	-3.23570000	0.04950000	2.72290000
C	-3.34840000	-2.57750000	2.13720000
H	-2.31380000	-2.88540000	2.31820000
H	-3.77660000	-2.27060000	3.09690000
H	-3.91530000	-3.44700000	1.79140000
C	-4.86880000	-1.06720000	0.82860000
H	-5.39890000	-1.88520000	0.33060000
H	-5.40790000	-0.85600000	1.75820000
H	-4.94500000	-0.17430000	0.20190000

Sum of electronic and thermal Energies= -1862.531757
Sum of electronic and thermal Enthalpies= -1862.530813
Sum of electronic and thermal Free Energies= -1862.624616

TS-2'-H(η^2-Et)- 2'-H(Et)

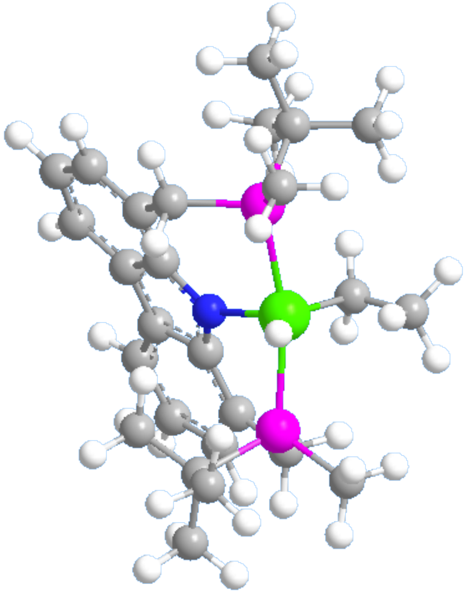

C	-3.36910000	2.80940000	-1.89510000
C	-3.67750000	1.44600000	-1.94060000
C	-2.82720000	0.45500000	-1.45270000
C	-1.59580000	0.87300000	-0.89420000
C	-1.31250000	2.26850000	-0.79840000
C	-2.18670000	3.22640000	-1.30920000
H	-4.07280000	3.53260000	-2.29270000
H	-4.63070000	1.13450000	-2.36190000
H	-1.94850000	4.28300000	-1.22720000
C	0.29880000	1.04710000	0.26010000
C	1.42940000	0.83590000	1.07960000
C	2.14840000	1.95840000	1.48140000
C	1.78550000	3.26350000	1.12710000
C	0.65490000	3.47780000	0.35940000
C	-0.09140000	2.37800000	-0.06490000
H	3.02660000	1.80410000	2.10460000
H	2.38470000	4.09950000	1.47120000
H	0.34330000	4.48460000	0.09650000
N	-0.59660000	0.14040000	-0.28030000
C	1.89140000	-0.51600000	1.54110000
H	1.19750000	-0.94040000	2.27880000
H	2.86500000	-0.43130000	2.03780000
C	-3.30600000	-0.97210000	-1.52070000
H	-4.40110000	-0.99860000	-1.49970000
H	-3.02000000	-1.44300000	-2.47280000
P	1.94210000	-1.77500000	0.20720000
P	-2.60320000	-2.08500000	-0.24810000
C	3.34950000	-1.26100000	-0.94220000

C	2.63610000	-3.21820000	1.09640000
C	-3.36550000	-3.68440000	-0.70550000
C	-3.42120000	-1.65170000	1.39140000
C	-0.24030000	-3.06960000	-2.72310000
C	-0.17650000	-1.60340000	-2.48280000
H	-1.01190000	-1.01050000	-2.85270000
H	0.59770000	-3.47360000	-3.30010000
H	0.76290000	-1.12720000	-2.75510000
Rh	-0.32280000	-1.92760000	-0.39430000
H	-0.17290000	-3.61340000	-1.72780000
H	-0.36350000	-3.10310000	0.81450000
C	4.68520000	-1.15400000	-0.20610000
H	5.03040000	-2.11440000	0.18430000
H	5.45420000	-0.79400000	-0.89890000
H	4.64290000	-0.43920000	0.62120000
C	3.03790000	0.09230000	-1.59090000
H	2.01560000	0.15800000	-1.96950000
H	3.17250000	0.92010000	-0.89260000
H	3.71920000	0.25360000	-2.43410000
C	-3.33370000	-2.88590000	2.29200000
H	-2.30510000	-3.25250000	2.36840000
H	-3.67130000	-2.62450000	3.30010000
H	-3.96630000	-3.70490000	1.93890000
C	-2.67040000	-0.49720000	2.05660000
H	-1.61400000	-0.74090000	2.19990000
H	-2.72420000	0.42530000	1.47370000
H	-3.11540000	-0.29930000	3.03810000
H	-3.13040000	-3.90520000	-1.74860000
H	3.60400000	-3.00360000	1.55600000
C	-4.88250000	-1.25800000	1.17760000
H	-5.45420000	-2.02740000	0.64840000
H	-5.36740000	-1.10940000	2.14850000
H	-4.97270000	-0.31950000	0.62330000
C	3.45070000	-2.33840000	-2.02570000
H	3.66710000	-3.32740000	-1.61020000
H	2.53080000	-2.41360000	-2.61120000
H	4.26250000	-2.09330000	-2.71930000
H	-2.95210000	-4.48460000	-0.09090000
H	-4.45370000	-3.66990000	-0.59380000
H	2.73980000	-4.07260000	0.42500000
H	1.92340000	-3.49870000	1.87290000
H	-1.17930000	-3.40700000	-3.17000000

Sum of electronic and thermal Energies= -1862.521497
Sum of electronic and thermal Enthalpies= -1862.520553
Sum of electronic and thermal Free Energies= -1862.615833

```
******    1 imaginary frequencies (negative Signs) ******
 Diagonal vibrational polarizability:
       30.3740741      42.2710141      23.8326875
 Harmonic frequencies (cm**-1), IR intensities (KM/Mole), Raman scattering
 activities (A**4/AMU), depolarization ratios for plane and unpolarized
 incident light, reduced masses (AMU), force constants (mDyne/A),
 and normal coordinates:
                      1                      2                      3
                      A                      A                      A
 Frequencies --   -344.0871                23.9471                45.5424
 Red. masses --      1.4492                 3.7724                 4.2359
 Frc consts  --      0.1011                 0.0013                 0.0052
 IR Inten    --     31.8141                 0.2951                 0.0845
```

2'-H(Et)

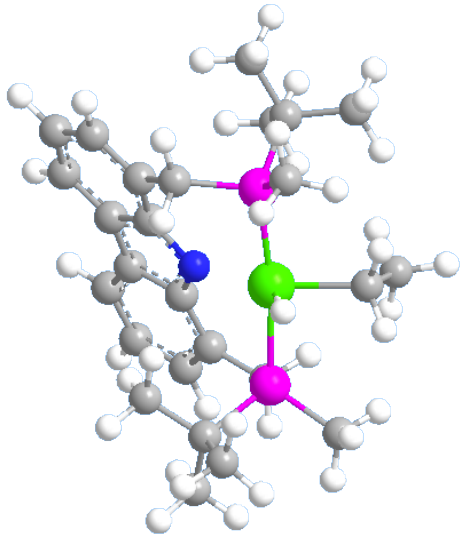

C	-2.97330000	2.93740000	-1.84880000
C	-3.30610000	1.57940000	-1.89410000
C	-2.47650000	0.57680000	-1.39520000
C	-1.25810000	0.97140000	-0.79840000
C	-0.92940000	2.35620000	-0.75190000
C	-1.77510000	3.33110000	-1.28080000
H	-3.65960000	3.67220000	-2.25580000
H	-4.25120000	1.28150000	-2.34290000
H	-1.50010000	4.38090000	-1.23220000
C	0.66290000	1.10880000	0.29090000
C	1.82680000	0.87790000	1.05700000
C	2.62770000	1.97590000	1.36210000
C	2.30790000	3.28130000	0.96980000
C	1.14200000	3.52130000	0.26490000
C	0.32150000	2.44200000	-0.06590000
H	3.53050000	1.80400000	1.94420000
H	2.96950000	4.09860000	1.23600000
H	0.86620000	4.53080000	-0.02650000
N	-0.27710000	0.20190000	-0.18050000
C	2.22110000	-0.47080000	1.58340000
H	1.54090000	-0.78520000	2.38840000
H	3.22650000	-0.42890000	2.01760000
C	-2.92240000	-0.84910000	-1.56370000
H	-4.00300000	-0.88550000	-1.74440000
H	-2.45030000	-1.28950000	-2.45510000
P	2.10040000	-1.82080000	0.35080000
P	-2.45700000	-1.99620000	-0.21450000
C	3.35530000	-1.42570000	-0.99560000
C	2.84460000	-3.22510000	1.25490000
C	-3.29810000	-3.53240000	-0.74140000

C	-3.40680000	-1.47190000	1.32220000
Rh	-0.16530000	-1.97180000	-0.09420000
C	-2.65050000	-0.34940000	2.03580000
H	-1.62670000	-0.64710000	2.28100000
H	-2.59410000	0.56000000	1.43340000
H	-3.17030000	-0.10200000	2.96830000
C	-4.81150000	-0.99370000	0.95730000
H	-5.38210000	-1.74580000	0.40280000
H	-5.37460000	-0.77290000	1.87070000
H	-4.78300000	-0.07610000	0.36270000
C	2.74360000	-0.41500000	-1.96940000
H	1.77620000	-0.75280000	-2.35370000
H	2.58910000	0.56150000	-1.50530000
H	3.41650000	-0.27640000	-2.82300000
C	4.64270000	-0.85310000	-0.40500000
H	5.09630000	-1.51660000	0.33830000
H	5.38210000	-0.71030000	-1.20100000
H	4.47370000	0.12210000	0.06000000
H	3.90940000	-3.07550000	1.45300000
H	-4.38500000	-3.41530000	-0.76220000
C	-3.48390000	-2.68690000	2.25020000
H	-3.91310000	-2.38640000	3.21160000
H	-4.11640000	-3.48180000	1.84570000
H	-2.49210000	-3.10590000	2.44920000
C	3.65580000	-2.72840000	-1.74180000
H	4.30550000	-2.51920000	-2.59820000
H	4.17270000	-3.45820000	-1.11250000
H	2.74670000	-3.19790000	-2.12930000
H	-2.96070000	-3.79730000	-1.74570000
H	-3.04190000	-4.35650000	-0.07430000
H	2.32200000	-3.33160000	2.20760000
H	2.71360000	-4.15450000	0.69860000
H	-0.25300000	-3.11040000	0.94750000
C	0.04440000	-3.79940000	-1.09660000
H	1.00710000	-4.27250000	-0.87310000
H	-0.72270000	-4.53080000	-0.82370000
C	-0.05350000	-3.44680000	-2.56710000
H	0.63830000	-2.64580000	-2.85220000
H	-1.05670000	-3.10740000	-2.84650000
H	0.17900000	-4.30770000	-3.21160000

Sum of electronic and thermal Energies= -1862.559610
Sum of electronic and thermal Enthalpies= -1862.558666
Sum of electronic and thermal Free Energies= -1862.654796

TS-2'-H(Et)-2’

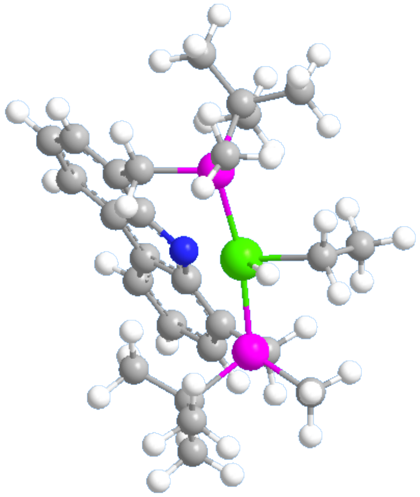

C	-2.95120000	3.02010000	-2.12460000
C	-3.27780000	1.66030000	-2.15880000
C	-2.46260000	0.66740000	-1.61930000
C	-1.25770000	1.07010000	-0.99810000
C	-0.94410000	2.45990000	-0.94980000
C	-1.77690000	3.42450000	-1.51680000
H	-3.62600000	3.74680000	-2.56390000
H	-4.21060000	1.35210000	-2.62610000
H	-1.50990000	4.47620000	-1.46440000
C	0.62320000	1.23850000	0.15930000
C	1.78050000	1.02860000	0.94110000
C	2.55310000	2.14010000	1.26710000
C	2.21800000	3.44120000	0.87280000
C	1.06900000	3.65890000	0.13450000
C	0.27520000	2.56580000	-0.21610000
H	3.44980000	1.98100000	1.86240000
H	2.85770000	4.27010000	1.15610000
H	0.78610000	4.66210000	-0.17150000
N	-0.28960000	0.32100000	-0.33780000
C	2.20690000	-0.31970000	1.43670000
H	1.53890000	-0.67480000	2.23440000
H	3.21470000	-0.26630000	1.86430000
C	-2.92500000	-0.75760000	-1.74130000
H	-4.00770000	-0.78820000	-1.91070000
H	-2.46250000	-1.24110000	-2.61510000
P	2.10760000	-1.62880000	0.15890000
P	-2.44410000	-1.83040000	-0.33920000
C	3.36920000	-1.18040000	-1.16650000
C	2.88910000	-3.03180000	1.04240000
C	-3.29950000	-3.39190000	-0.77600000
C	-3.39190000	-1.23480000	1.17690000

Rh	-0.15400000	-1.80390000	-0.29240000
C	-2.63910000	-0.08270000	1.84510000
H	-1.60980000	-0.36300000	2.08720000
H	-2.59950000	0.80770000	1.21400000
H	-3.15220000	0.18870000	2.77470000
C	-4.79810000	-0.77580000	0.79460000
H	-5.37000000	-1.55270000	0.27680000
H	-5.35910000	-0.51310000	1.69820000
H	-4.77150000	0.11340000	0.15810000
C	2.72170000	-0.19850000	-2.14660000
H	1.78200000	-0.59100000	-2.54890000
H	2.50110000	0.76410000	-1.67940000
H	3.40170000	-0.01460000	-2.98610000
C	4.62740000	-0.55870000	-0.56440000
H	5.09760000	-1.20540000	0.18370000
H	5.37000000	-0.38620000	-1.35160000
H	4.41550000	0.40830000	-0.09960000
H	3.93560000	-2.82850000	1.28730000
H	-4.38680000	-3.27490000	-0.77230000
C	-3.46370000	-2.40890000	2.15710000
H	-3.89080000	-2.06820000	3.10590000
H	-4.09440000	-3.22330000	1.79040000
H	-2.46900000	-2.81430000	2.37150000
C	3.72670000	-2.47010000	-1.91020000
H	4.34770000	-2.23490000	-2.78110000
H	4.29280000	-3.16660000	-1.28550000
H	2.83630000	-2.99130000	-2.27780000
H	-2.99490000	-3.70210000	-1.77780000
H	-3.03110000	-4.18880000	-0.08080000
H	2.34060000	-3.19790000	1.97190000
H	2.84120000	-3.95050000	0.45630000
H	-0.17710000	-3.31200000	0.23730000
C	0.05370000	-3.87840000	-1.02390000
H	1.03570000	-4.31550000	-0.81970000
H	-0.67480000	-4.66210000	-0.77820000
C	-0.07150000	-3.56280000	-2.51180000
H	0.63970000	-2.80090000	-2.83950000
H	-1.06870000	-3.20580000	-2.77950000
H	0.11690000	-4.46940000	-3.10590000

Sum of electronic and thermal Energies= -1862.550248
Sum of electronic and thermal Enthalpies= -1862.549304
Sum of electronic and thermal Free Energies= -1862.645201

****** 1 imaginary frequencies (negative Signs) ******
Diagonal vibrational polarizability:

32.9853176 46.0106998 23.7924185

Harmonic frequencies (cm**-1), IR intensities (KM/Mole), Raman scattering activities (A**4/AMU), depolarization ratios for plane and unpolarized incident light, reduced masses (AMU), force constants (mDyne/A), and normal coordinates:

	1	2	3
	A	A	A
Frequencies --	-586.8528	30.2108	40.2955
Red. masses --	1.2988	4.0119	4.1750
Frc consts --	0.2635	0.0022	0.0040
IR Inten --	214.2577	0.2043	0.1861

2'-H_2(OCTENE) (n-octane analog)

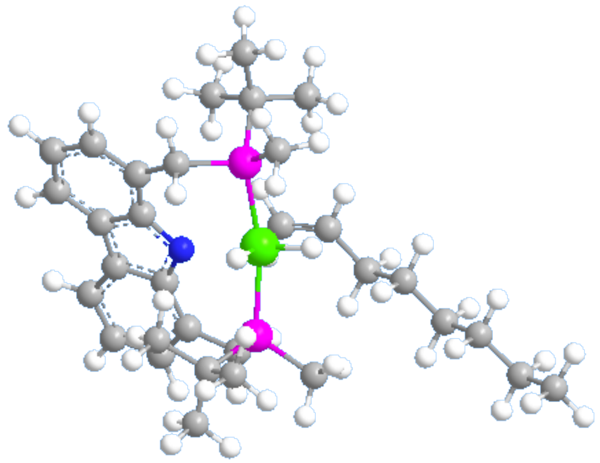

C	-3.97560000	-4.40790000	-2.84060000
C	-2.63890000	-4.14940000	-2.51800000
C	-2.22770000	-3.00360000	-1.83850000
C	-3.22490000	-2.06940000	-1.46730000
C	-4.59350000	-2.36710000	-1.75390000
C	-4.96120000	-3.51820000	-2.44910000
H	-4.23270000	-5.31760000	-3.37260000
H	-1.87940000	-4.87750000	-2.79530000
H	-6.00770000	-3.72030000	-2.66030000
C	-4.40150000	-0.46220000	-0.52120000
C	-4.86070000	0.63720000	0.23930000
C	-6.23550000	0.85190000	0.29460000
C	-7.16400000	0.02450000	-0.34910000
C	-6.72430000	-1.07300000	-1.06840000
C	-5.35390000	-1.32150000	-1.14790000
H	-6.59660000	1.70090000	0.87190000
H	-8.22360000	0.24240000	-0.26810000
H	-7.43200000	-1.73660000	-1.55780000
N	-3.11400000	-0.89650000	-0.75160000
C	-3.96410000	1.56730000	1.00710000
H	-3.54860000	1.06990000	1.89340000
H	-4.54840000	2.41820000	1.37640000
C	-0.75860000	-2.87300000	-1.52580000
H	-0.30810000	-3.86850000	-1.43700000
H	-0.21730000	-2.38320000	-2.34830000
P	-2.47420000	2.17540000	0.11440000
P	-0.33560000	-1.87100000	-0.05090000
C	-3.13620000	3.45540000	-1.11250000
C	-1.68240000	3.20890000	1.40050000
C	1.48080000	-2.10000000	0.03560000
C	-0.91710000	-2.85490000	1.45620000
Rh	-1.24350000	0.22900000	-0.31930000

C	-2.41120000	-2.67100000	1.72440000
H	-2.67800000	-1.62170000	1.86380000
H	-3.03050000	-3.06910000	0.91830000
H	-2.66800000	-3.21320000	2.64150000
C	-0.64430000	-4.34690000	1.25020000
H	0.40110000	-4.56760000	1.01560000
H	-0.88470000	-4.88760000	2.17170000
H	-1.27160000	-4.76480000	0.45790000
C	-4.20870000	2.82110000	-2.00200000
H	-3.90260000	1.85540000	-2.41400000
H	-5.14290000	2.65310000	-1.46370000
H	-4.42230000	3.48970000	-2.84360000
C	-3.74310000	4.65440000	-0.38100000
H	-2.99060000	5.24480000	0.14760000
H	-4.22060000	5.31760000	-1.11080000
H	-4.51520000	4.36070000	0.33620000
H	-2.38560000	3.90100000	1.86930000
H	1.74630000	-3.12770000	0.29650000
C	-0.13130000	-2.33820000	2.66480000
H	-0.51460000	-2.80860000	3.57600000
H	0.93520000	-2.57040000	2.60310000
H	-0.24020000	-1.25520000	2.78180000
C	-1.97680000	3.93970000	-1.98510000
H	-2.30130000	4.79570000	-2.58700000
H	-1.11270000	4.26660000	-1.39670000
H	-1.64460000	3.16580000	-2.67990000
H	1.92520000	-1.87050000	-0.93330000
H	1.91210000	-1.42320000	0.77450000
H	-1.27090000	2.54440000	2.16090000
H	-0.85170000	3.77590000	0.97480000
H	0.04510000	0.96540000	0.21880000
H	-1.47980000	0.09210000	1.22540000
C	-1.24540000	0.34640000	-2.68340000
H	-2.15320000	0.92070000	-2.81640000
C	-0.06680000	0.93430000	-2.32530000
H	-0.05320000	2.00300000	-2.12630000
H	-1.27820000	-0.66580000	-3.07400000
C	1.28500000	0.33280000	-2.55330000
C	2.37220000	0.79170000	-1.59390000
H	1.58700000	0.60910000	-3.57600000
H	1.21230000	-0.76230000	-2.57050000
C	3.74220000	0.21780000	-1.91830000
H	2.42070000	1.88930000	-1.60590000
H	2.08440000	0.52960000	-0.56950000
H	3.67590000	-0.87850000	-1.98610000
H	4.05460000	0.54800000	-2.91910000
C	4.80960000	0.59040000	-0.90180000

C	6.18780000	0.03510000	-1.22960000
H	4.50120000	0.23810000	0.09330000
H	4.86770000	1.68470000	-0.81690000
C	7.24170000	0.41010000	-0.20300000
H	6.12540000	-1.05750000	-1.31650000
H	6.49290000	0.39050000	-2.22240000
H	8.22360000	0.00360000	-0.45620000
H	7.34770000	1.49620000	-0.12130000
H	6.97850000	0.03640000	0.79130000

Sum of electronic and thermal Energies= -2098.276145
Sum of electronic and thermal Enthalpies= -2098.275201
Sum of electronic and thermal Free Energies= -2098.390049

TS-2’-H_2(OCTENE)- 2'-H(η^2-octyl)

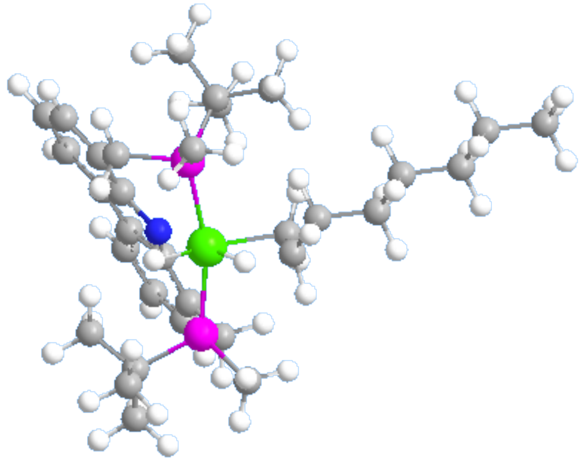

C	7.01570000	-0.89210000	3.03830000
C	6.11610000	-1.84670000	2.55220000
C	5.17160000	-1.57040000	1.56560000
C	5.12980000	-0.25640000	1.04290000
C	6.09440000	0.69210000	1.49980000
C	7.01420000	0.38240000	2.50090000
H	7.72580000	-1.16630000	3.81100000
H	6.15660000	-2.85970000	2.94690000
H	7.73100000	1.12780000	2.83390000
C	4.85530000	1.54870000	-0.20810000
C	4.47150000	2.51620000	-1.16480000
C	5.13130000	3.74230000	-1.14710000
C	6.15620000	4.04480000	-0.24220000
C	6.55140000	3.09710000	0.68420000
C	5.91080000	1.85750000	0.69870000
H	4.83300000	4.49070000	-1.87850000
H	6.63580000	5.01700000	-0.27790000
H	7.34820000	3.30890000	1.39170000
N	4.35490000	0.28290000	0.03180000
C	3.38100000	2.30490000	-2.17140000
H	3.68560000	1.60680000	-2.95880000
H	3.13100000	3.25460000	-2.65680000
C	4.30470000	-2.70580000	1.09720000
H	4.81460000	-3.66090000	1.26830000
H	3.37050000	-2.75880000	1.67190000
P	1.87430000	1.54360000	-1.44900000
P	3.75510000	-2.58460000	-0.64270000
Rh	2.59240000	-0.61740000	-0.84940000
C	1.28520000	-0.94150000	0.89030000
H	0.85880000	-0.03530000	1.31300000
C	0.61680000	-1.52210000	-0.24480000
H	0.63510000	-2.61450000	-0.27750000

H	1.70660000	-1.60580000	1.63900000
H	3.28150000	-0.53240000	-2.31880000
H	1.27730000	-1.37260000	-1.48670000
C	0.72570000	1.65020000	-2.87190000
H	-0.23110000	1.17210000	-2.65970000
H	0.54600000	2.68490000	-3.17640000
H	1.18760000	1.11510000	-3.70430000
C	2.80100000	-4.13870000	-0.81900000
H	2.23150000	-4.13930000	-1.74970000
H	3.45110000	-5.01700000	-0.80150000
H	2.10040000	-4.22470000	0.01440000
C	1.23610000	2.84070000	-0.22270000
C	2.04900000	2.76700000	1.07560000
H	2.16010000	1.74090000	1.43670000
H	3.05070000	3.18260000	0.95510000
H	1.54040000	3.34900000	1.85250000
C	-0.23380000	2.55580000	0.09740000
H	-0.62030000	3.35050000	0.74480000
H	-0.86800000	2.53000000	-0.79480000
H	-0.36290000	1.61470000	0.63760000
C	1.35420000	4.24830000	-0.81040000
H	0.92270000	4.97010000	-0.10800000
H	2.39670000	4.53610000	-0.96610000
H	0.81550000	4.36110000	-1.75640000
C	5.26130000	-2.87180000	-1.73400000
C	6.09290000	-1.59530000	-1.86630000
H	5.49590000	-0.76270000	-2.24470000
H	6.53510000	-1.28990000	-0.91530000
H	6.91160000	-1.78130000	-2.57030000
C	4.74630000	-3.27810000	-3.11680000
H	4.05360000	-2.53120000	-3.51710000
H	5.58920000	-3.35500000	-3.81100000
H	4.24260000	-4.24900000	-3.10780000
C	6.13890000	-3.97910000	-1.14710000
H	5.59720000	-4.91580000	-0.98290000
H	6.95520000	-4.20040000	-1.84280000
H	6.59470000	-3.67340000	-0.20130000
C	-0.72550000	-0.97560000	-0.69700000
C	-1.84430000	-1.29820000	0.28440000
H	-0.98090000	-1.36210000	-1.69260000
H	-0.64780000	0.10930000	-0.80760000
C	-3.18330000	-0.71610000	-0.14240000
H	-1.93310000	-2.38710000	0.40200000
H	-1.57210000	-0.91590000	1.27710000
H	-3.08310000	0.37330000	-0.25620000
H	-3.44490000	-1.09120000	-1.14190000
C	-4.31700000	-1.02090000	0.82390000

C	-5.65460000	-0.43430000	0.39660000
H	-4.05630000	-0.64530000	1.82330000
H	-4.41650000	-2.10940000	0.93860000
C	-6.78000000	-0.74610000	1.36700000
H	-5.55200000	0.65270000	0.28320000
H	-5.91050000	-0.81000000	-0.60270000
H	-7.73100000	-0.31740000	1.04290000
H	-6.92580000	-1.82550000	1.47240000
H	-6.56510000	-0.35100000	2.36450000

Sum of electronic and thermal Energies= -2098.259279
Sum of electronic and thermal Enthalpies= -2098.258334
Sum of electronic and thermal Free Energies= -2098.371120

****** 1 imaginary frequencies (negative Signs) ******
Diagonal vibrational polarizability:
50.6735704 30.0818854 34.8044040
Harmonic frequencies (cm**-1), IR intensities (KM/Mole), Raman scattering activities (A**4/AMU), depolarization ratios for plane and unpolarized incident light, reduced masses (AMU), force constants (mDyne/A), and normal coordinates:

	1	2	3
	A	A	A
Frequencies --	-557.5557	14.6034	21.3833
Red. masses --	1.3656	4.2615	4.4288
Frc consts --	0.2501	0.0005	0.0012
IR Inten --	1.4773	0.1660	0.0754

2'-H(η^2-octyl) (n-octane analog)

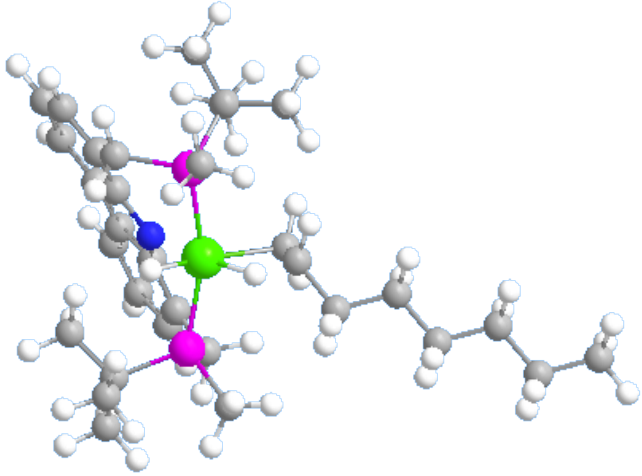

C	4.71570000	-3.34600000	3.27570000
C	3.54410000	-3.53530000	2.53550000
C	3.12350000	-2.66040000	1.53590000
C	3.92940000	-1.52750000	1.27480000
C	5.15540000	-1.37710000	1.98960000
C	5.53470000	-2.26790000	2.99310000
H	4.98830000	-4.06150000	4.04400000
H	2.93180000	-4.41270000	2.73180000
H	6.47060000	-2.12430000	3.52560000
C	4.96140000	0.21850000	0.37260000
C	5.38000000	1.31880000	-0.41110000
C	6.59990000	1.91150000	-0.09640000
C	7.42490000	1.45580000	0.93910000
C	7.03280000	0.36720000	1.69650000
C	5.81420000	-0.25050000	1.41390000
H	6.91980000	2.76350000	-0.69270000
H	8.36700000	1.95590000	1.13580000
H	7.66080000	-0.00640000	2.50040000
N	3.80280000	-0.53450000	0.31870000
C	4.56670000	1.89980000	-1.52960000
H	4.54700000	1.24070000	-2.40480000
H	4.99320000	2.85740000	-1.84760000
C	1.87420000	-3.02030000	0.77990000
H	1.73000000	-4.10690000	0.79260000
H	0.98320000	-2.59010000	1.25740000
P	2.80680000	2.09220000	-1.06240000
P	1.80910000	-2.37560000	-0.92970000
C	2.79310000	3.40600000	0.29330000
C	2.09640000	2.94120000	-2.51950000
C	0.23350000	-3.10390000	-1.52440000
C	3.09020000	-3.32310000	-1.94330000

C	-0.15720000	0.57990000	-0.39570000
C	0.59730000	0.25770000	0.82100000
H	0.24570000	-0.61690000	1.37010000
H	-0.28310000	1.65670000	-0.56010000
H	0.83930000	1.07990000	1.48890000
Rh	2.03300000	-0.08600000	-0.77220000
H	0.45350000	0.30240000	-1.43620000
H	2.76720000	-0.23970000	-2.23200000
C	3.70720000	4.57980000	-0.05900000
H	3.44420000	5.05380000	-1.00970000
H	3.62690000	5.34970000	0.71670000
H	4.75660000	4.27560000	-0.10140000
C	3.25610000	2.78940000	1.61900000
H	2.77890000	1.82670000	1.81950000
H	4.33530000	2.62870000	1.63810000
H	3.00820000	3.47090000	2.44070000
C	2.67920000	-3.20260000	-3.41340000
H	2.58290000	-2.15390000	-3.71140000
H	3.44990000	-3.65740000	-4.04400000
H	1.73730000	-3.71300000	-3.63220000
C	4.49120000	-2.73430000	-1.77900000
H	4.51360000	-1.67390000	-2.03890000
H	4.87220000	-2.84410000	-0.76150000
H	5.17450000	-3.26790000	-2.44920000
H	-0.52270000	-3.01700000	-0.74350000
H	2.50580000	3.94520000	-2.65960000
C	3.11790000	-4.79210000	-1.51640000
H	2.13800000	-5.27630000	-1.57080000
H	3.78620000	-5.34970000	-2.18110000
H	3.50270000	-4.90810000	-0.49930000
C	1.35340000	3.90520000	0.44330000
H	0.97040000	4.36470000	-0.47280000
H	0.67070000	3.10330000	0.73700000
H	1.31110000	4.66690000	1.22930000
H	-0.12640000	-2.58290000	-2.41300000
H	0.34970000	-4.16440000	-1.76110000
H	1.01010000	3.01140000	-2.44000000
H	2.32710000	2.33810000	-3.39950000
C	-1.48140000	-0.14390000	-0.61640000
C	-2.57990000	0.43860000	0.26280000
H	-1.35210000	-1.20430000	-0.37570000
H	-1.78640000	-0.10220000	-1.67040000
C	-3.91010000	-0.28540000	0.12340000
H	-2.25020000	0.41160000	1.30980000
H	-2.71400000	1.50270000	0.02320000
H	-4.23420000	-0.26150000	-0.92650000
H	-3.77220000	-1.34930000	0.36260000

C	-5.00720000	0.29090000	1.00500000
C	-6.33610000	-0.44010000	0.88170000
H	-5.15140000	1.35300000	0.76180000
H	-4.67850000	0.27450000	2.05360000
C	-7.42180000	0.14410000	1.76810000
H	-6.66230000	-0.42270000	-0.16640000
H	-6.18850000	-1.50030000	1.12490000
H	-8.36700000	-0.39420000	1.66710000
H	-7.13480000	0.10770000	2.82340000
H	-7.61370000	1.19290000	1.52220000

Sum of electronic and thermal Energies= -2098.255382
Sum of electronic and thermal Enthalpies= -2098.254438
Sum of electronic and thermal Free Energies= -2098.369139

TS-2'-H(η^2-octyl)- 2'-H(octyl) (n-octane analog)

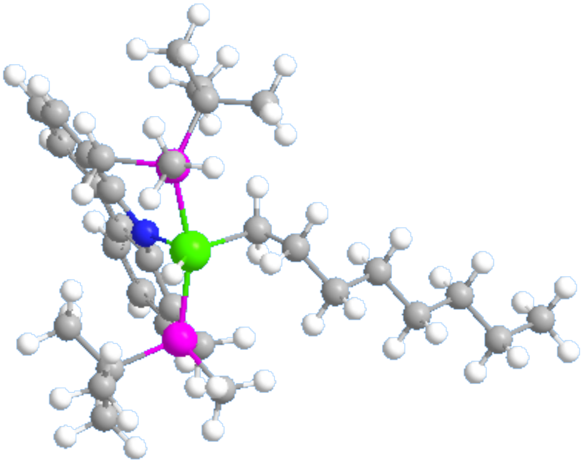

C	-2.13530000	3.38710000	3.22080000
C	-1.43450000	3.29630000	2.01370000
C	-1.77110000	2.39690000	1.00340000
C	-2.87100000	1.53880000	1.23860000
C	-3.62330000	1.68220000	2.44290000
C	-3.24570000	2.58830000	3.43250000
H	-1.82370000	4.10770000	3.96930000
H	-0.59720000	3.96800000	1.83790000
H	-3.83020000	2.67840000	4.34370000
C	-4.63470000	0.20020000	1.03240000
C	-5.67300000	-0.63430000	0.56410000
C	-6.74740000	-0.86180000	1.41970000
C	-6.84110000	-0.28860000	2.69400000
C	-5.84590000	0.56250000	3.14040000
C	-4.75280000	0.81670000	2.31140000
H	-7.54800000	-1.51070000	1.07100000
H	-7.70340000	-0.50220000	3.31640000
H	-5.91590000	1.03740000	4.11480000
N	-3.46840000	0.60760000	0.41010000
C	-5.68110000	-1.28020000	-0.79120000
H	-5.82360000	-0.53500000	-1.58500000
H	-6.51790000	-1.98450000	-0.86710000
C	-0.97450000	2.44810000	-0.27460000
H	-0.57940000	3.45880000	-0.42650000
H	-0.09230000	1.79360000	-0.21690000
P	-4.09460000	-2.09630000	-1.22820000
P	-1.86470000	1.86460000	-1.76620000
C	-3.95530000	-3.58710000	-0.07280000
C	-4.51220000	-2.85950000	-2.84050000
C	-0.58530000	2.04770000	-3.06190000
C	-3.12550000	3.19090000	-2.22150000
C	-0.32230000	-1.44770000	-1.15590000

C	-1.12710000	-0.93480000	-0.01080000
H	-0.67930000	-0.10940000	0.54710000
H	-0.28710000	-2.54440000	-1.17870000
H	-1.51320000	-1.68170000	0.67980000
Rh	-2.64350000	-0.23860000	-1.31520000
H	-0.89970000	-1.19860000	-2.10730000
H	-3.12490000	-0.17440000	-2.92960000
C	-5.14810000	-4.53310000	-0.21530000
H	-5.20110000	-5.00020000	-1.20180000
H	-5.05910000	-5.34210000	0.51860000
H	-6.09940000	-4.02940000	-0.01950000
C	-3.86400000	-3.12960000	1.38710000
H	-3.16200000	-2.30540000	1.53160000
H	-4.82870000	-2.79910000	1.77490000
H	-3.52630000	-3.96900000	2.00580000
C	-3.43130000	3.04440000	-3.71410000
H	-3.72380000	2.01930000	-3.96340000
H	-4.26390000	3.70370000	-3.97970000
H	-2.58210000	3.32320000	-4.34370000
C	-4.41870000	2.98670000	-1.43160000
H	-4.82970000	1.98650000	-1.59260000
H	-4.27620000	3.12130000	-0.35700000
H	-5.15990000	3.72180000	-1.76480000
H	0.30540000	1.48790000	-2.77320000
H	-5.37610000	-3.52590000	-2.78020000
C	-2.56650000	4.58410000	-1.93330000
H	-1.60820000	4.76660000	-2.43050000
H	-3.26710000	5.34210000	-2.30010000
H	-2.43650000	4.75480000	-0.86100000
C	-2.67060000	-4.32950000	-0.44880000
H	-2.65460000	-4.63790000	-1.49880000
H	-1.78390000	-3.71900000	-0.26190000
H	-2.57610000	-5.23720000	0.15740000
H	-0.95130000	1.63430000	-4.00220000
H	-0.30220000	3.09340000	-3.21480000
H	-3.65960000	-3.41290000	-3.23860000
H	-4.73000000	-2.05020000	-3.53850000
C	1.08330000	-0.87120000	-1.28580000
C	2.02740000	-1.36790000	-0.20010000
H	1.02860000	0.22470000	-1.23230000
H	1.50030000	-1.10500000	-2.27390000
C	3.42230000	-0.77000000	-0.29600000
H	1.59560000	-1.14270000	0.78470000
H	2.09220000	-2.46400000	-0.24830000
H	3.84770000	-0.99000000	-1.28550000
H	3.35190000	0.32590000	-0.24580000
C	4.37330000	-1.26320000	0.78330000

C	5.76750000	-0.66090000	0.68760000
H	4.44560000	-2.35890000	0.73390000
H	3.94830000	-1.04360000	1.77260000
C	6.70860000	-1.15950000	1.76990000
H	6.18890000	-0.88140000	-0.30190000
H	5.69180000	0.43310000	0.73630000
H	7.70340000	-0.71620000	1.68420000
H	6.32800000	-0.92070000	2.76770000
H	6.82850000	-2.24610000	1.72230000

Sum of electronic and thermal Energies= -2098.246818
Sum of electronic and thermal Enthalpies= -2098.245873
Sum of electronic and thermal Free Energies= -2098.361145

****** 1 imaginary frequencies (negative Signs) ******
Diagonal vibrational polarizability:
36.0654781 34.7420488 40.2670368
Harmonic frequencies (cm**-1), IR intensities (KM/Mole), Raman scattering activities (A**4/AMU), depolarization ratios for plane and unpolarized incident light, reduced masses (AMU), force constants (mDyne/A), and normal coordinates:

	1	2	3
	A	A	A
Frequencies --	-294.5537	14.7694	20.3334
Red. masses --	1.5033	4.2825	4.4228
Frc consts --	0.0768	0.0006	0.0011
IR Inten --	23.2795	0.0674	0.1082

2'-H(octyl) (n-octane analog)

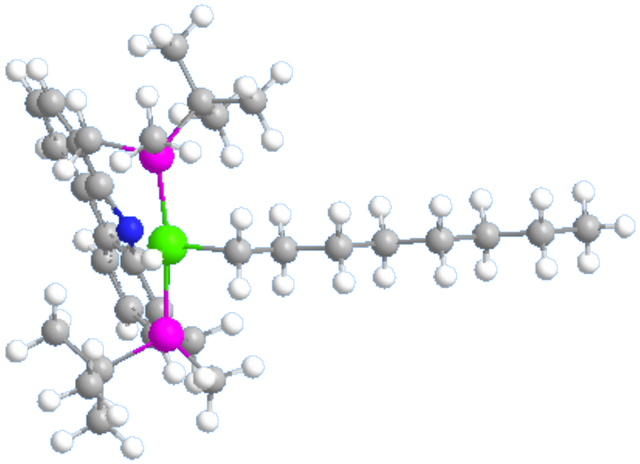

C	5.62340000	-2.45570000	3.37780000
C	4.71370000	-2.96810000	2.44660000
C	4.27670000	-2.24800000	1.33630000
C	4.80720000	-0.95240000	1.14540000
C	5.73380000	-0.43930000	2.09710000
C	6.13280000	-1.18080000	3.20910000
H	5.92480000	-3.06350000	4.22420000
H	4.31810000	-3.97110000	2.59070000
H	6.84180000	-0.76590000	3.91970000
C	5.41390000	1.03980000	0.39740000
C	5.64190000	2.21450000	-0.35320000
C	6.49040000	3.17510000	0.19200000
C	7.13910000	3.00560000	1.42090000
C	6.96190000	1.83480000	2.13630000
C	6.10930000	0.85600000	1.62390000
H	6.66310000	4.08640000	-0.37650000
H	7.78960000	3.78810000	1.79660000
H	7.47570000	1.67650000	3.08020000
N	4.59370000	-0.04420000	0.11310000
C	5.05330000	2.46180000	-1.71130000
H	5.52640000	1.81010000	-2.46050000
H	5.24490000	3.49430000	-2.02470000
C	3.24640000	-2.88670000	0.44710000
H	3.22910000	-3.97050000	0.61050000
H	2.24150000	-2.52260000	0.71040000
P	3.27090000	2.06170000	-1.84360000
P	3.39010000	-2.50620000	-1.33830000
C	2.37080000	3.25020000	-0.69380000
C	2.87220000	2.67520000	-3.51940000
C	2.08070000	-3.57270000	-2.04140000
C	4.98590000	-3.28900000	-1.95300000
Rh	3.18260000	-0.22860000	-1.53500000

C	6.15980000	-2.34980000	-1.66710000
H	6.00020000	-1.36090000	-2.10720000
H	6.32720000	-2.21070000	-0.59670000
H	7.07380000	-2.77500000	-2.09670000
C	5.22840000	-4.63680000	-1.27510000
H	4.39400000	-5.33260000	-1.41060000
H	6.11610000	-5.11230000	-1.70640000
H	5.40970000	-4.52160000	-0.20260000
C	2.43180000	2.70540000	0.73570000
H	2.06280000	1.67680000	0.79540000
H	3.44800000	2.71340000	1.13600000
H	1.80990000	3.32630000	1.39040000
C	2.99490000	4.64400000	-0.73770000
H	3.02330000	5.06080000	-1.74960000
H	2.40370000	5.33260000	-0.12360000
H	4.01220000	4.64220000	-0.33630000
H	2.96060000	3.76230000	-3.59590000
H	2.28550000	-4.63360000	-1.87380000
C	4.85340000	-3.47230000	-3.46700000
H	5.81160000	-3.80300000	-3.88090000
H	4.10690000	-4.22580000	-3.73240000
H	4.58580000	-2.53460000	-3.96490000
C	0.91000000	3.31560000	-1.14800000
H	0.33180000	3.91800000	-0.43920000
H	0.80090000	3.77840000	-2.13290000
H	0.44800000	2.32430000	-1.18640000
H	1.12750000	-3.32620000	-1.56800000
H	1.97730000	-3.39560000	-3.11280000
H	3.56840000	2.21630000	-4.22420000
H	1.86280000	2.37910000	-3.80880000
H	3.19150000	-0.39770000	-3.07130000
C	1.20170000	-0.34090000	-2.20630000
H	0.91310000	0.55570000	-2.76960000
H	1.02090000	-1.18670000	-2.87930000
C	0.34560000	-0.47010000	-0.95900000
H	0.60750000	0.31030000	-0.22900000
H	0.55780000	-1.41860000	-0.44450000
C	-1.15440000	-0.39030000	-1.24610000
C	-2.01820000	-0.45640000	0.00400000
H	-1.43450000	-1.19910000	-1.93560000
H	-1.36740000	0.54160000	-1.78910000
C	-3.50990000	-0.37640000	-0.28230000
H	-1.80020000	-1.38390000	0.55210000
H	-1.73240000	0.35800000	0.68490000
H	-3.72550000	0.55040000	-0.83250000
H	-3.79510000	-1.19150000	-0.96240000
C	-4.37620000	-0.43470000	0.96620000

C	-5.86810000	-0.35150000	0.67790000
H	-4.09100000	0.37960000	1.64720000
H	-4.16280000	-1.36190000	1.51660000
C	-6.72270000	-0.40970000	1.93170000
H	-6.07810000	0.57490000	0.12770000
H	-6.15020000	-1.16580000	-0.00230000
H	-7.78960000	-0.34890000	1.70420000
H	-6.55740000	-1.34090000	2.48210000
H	-6.48520000	0.41340000	2.61260000

Sum of electronic and thermal Energies= -2098.285783
Sum of electronic and thermal Enthalpies= -2098.284839
Sum of electronic and thermal Free Energies= -2098.402795

TS-2'-H(η²-octyl)-2' (n-octane analog)

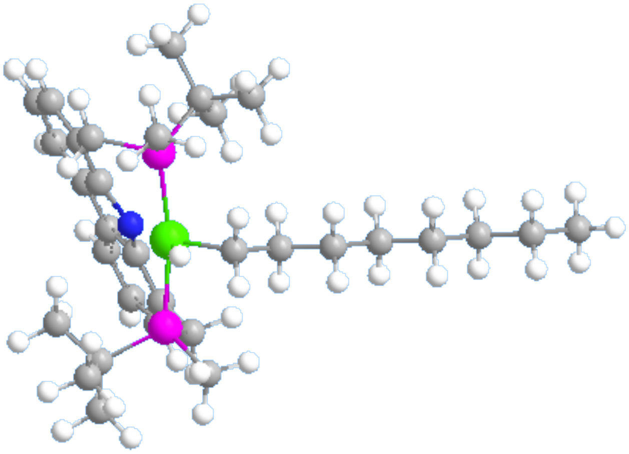

C	5.59710000	-2.44360000	3.32120000
C	4.63360000	-2.95150000	2.44310000
C	4.16750000	-2.24800000	1.33450000
C	4.71510000	-0.96730000	1.08930000
C	5.70900000	-0.46600000	1.97990000
C	6.13870000	-1.19240000	3.09040000
H	5.91900000	-3.03850000	4.16920000
H	4.22170000	-3.94170000	2.62540000
H	6.89790000	-0.78330000	3.75100000
C	5.35660000	0.98500000	0.26270000
C	5.56810000	2.15090000	-0.50630000
C	6.48140000	3.08660000	-0.02770000
C	7.20370000	2.90670000	1.15810000
C	7.02590000	1.75340000	1.90040000
C	6.11100000	0.79830000	1.45450000
H	6.64040000	3.98960000	-0.61330000
H	7.90330000	3.66970000	1.48230000
H	7.58440000	1.59040000	2.81780000
N	4.48710000	-0.07370000	0.04860000
C	4.87150000	2.42190000	-1.80510000
H	5.25880000	1.77020000	-2.60140000
H	5.05020000	3.45540000	-2.12350000
C	3.11540000	-2.90180000	0.48340000
H	3.11470000	-3.98510000	0.65170000
H	2.11290000	-2.54460000	0.76390000
P	3.07800000	2.05150000	-1.76490000
P	3.23680000	-2.50740000	-1.29930000
C	2.30470000	3.26530000	-0.54880000
C	2.55940000	2.69180000	-3.40240000
C	1.92640000	-3.58530000	-1.99380000
C	4.82400000	-3.29130000	-1.94430000
Rh	2.98050000	-0.23750000	-1.44770000

C	6.00880000	−2.35910000	−1.68320000
H	5.83670000	−1.36370000	−2.10330000
H	6.21290000	−2.23680000	−0.61730000
H	6.90640000	−2.78120000	−2.14930000
C	5.07600000	−4.64250000	−1.27730000
H	4.23820000	−5.33660000	−1.40040000
H	5.95510000	−5.11790000	−1.72610000
H	5.27650000	−4.53180000	−0.20770000
C	2.43030000	2.68810000	0.86350000
H	2.00690000	1.68020000	0.92600000
H	3.47050000	2.62700000	1.19170000
H	1.89300000	3.32860000	1.57210000
C	2.97830000	4.63430000	−0.61160000
H	2.95920000	5.06490000	−1.61820000
H	2.45580000	5.33660000	0.04780000
H	4.01840000	4.58590000	−0.27730000
H	2.71850000	3.77070000	−3.48730000
H	2.14930000	−4.64520000	−1.84290000
C	4.66080000	−3.46700000	−3.45660000
H	5.61080000	−3.79400000	−3.89180000
H	3.91000000	−4.21950000	−3.71250000
H	4.38350000	−2.52540000	−3.94260000
C	0.82180000	3.39450000	−0.90840000
H	0.30580000	3.98160000	−0.14120000
H	0.66850000	3.90230000	−1.86470000
H	0.32530000	2.41920000	−0.95920000
H	0.97790000	−3.36290000	−1.49950000
H	1.80000000	−3.40310000	−3.06210000
H	3.15440000	2.19130000	−4.16920000
H	1.50840000	2.47820000	−3.60150000
H	2.27420000	−0.43730000	−2.86810000
C	0.97340000	−0.36340000	−2.35980000
H	0.66570000	0.54590000	−2.88810000
H	0.68220000	−1.19490000	−3.01680000
C	0.16550000	−0.48500000	−1.06490000
H	0.45670000	0.29760000	−0.35400000
H	0.39000000	−1.43170000	−0.55870000
C	−1.33780000	−0.39800000	−1.32550000
C	−2.17250000	−0.45640000	−0.05540000
H	−1.63940000	−1.20740000	−2.00560000
H	−1.55840000	0.53490000	−1.86390000
C	−3.66990000	−0.37120000	−0.30820000
H	−1.94550000	−1.38300000	0.49010000
H	−1.86750000	0.35900000	0.61580000
H	−3.89460000	0.55590000	−0.85410000
H	−3.97310000	−1.18590000	−0.98080000
C	−4.50770000	−0.42550000	0.95980000

C	-6.00530000	-0.33830000	0.70480000
H	-4.20480000	0.38850000	1.63330000
H	-4.28460000	-1.35290000	1.50600000
C	-6.83190000	-0.39440000	1.97730000
H	-6.22500000	0.58880000	0.15940000
H	-6.30460000	-1.15180000	0.03110000
H	-7.90330000	-0.33040000	1.77350000
H	-6.65700000	-1.32620000	2.52370000
H	-6.57690000	0.42780000	2.65290000

Sum of electronic and thermal Energies= -2098.276592
Sum of electronic and thermal Enthalpies= -2098.275648
Sum of electronic and thermal Free Energies= -2098.392760

```
******    1 imaginary frequencies (negative Signs) ******
 Diagonal vibrational polarizability:
       59.8125191      36.1994885      38.8294816
 Harmonic frequencies (cm**-1), IR intensities (KM/Mole), Raman scattering
 activities (A**4/AMU), depolarization ratios for plane and unpolarized
 incident light, reduced masses (AMU), force constants (mDyne/A),
 and normal coordinates:
                      1                      2                      3
                      A                      A                      A
 Frequencies --   -568.8818                11.6646                16.2041
 Red. masses --      1.3131                 4.3116                 4.6819
 Frc consts  --      0.2504                 0.0003                 0.0007
 IR Inten    --    249.5308                 0.0316                 0.1947
```

2'-H_2(cyclooctane)

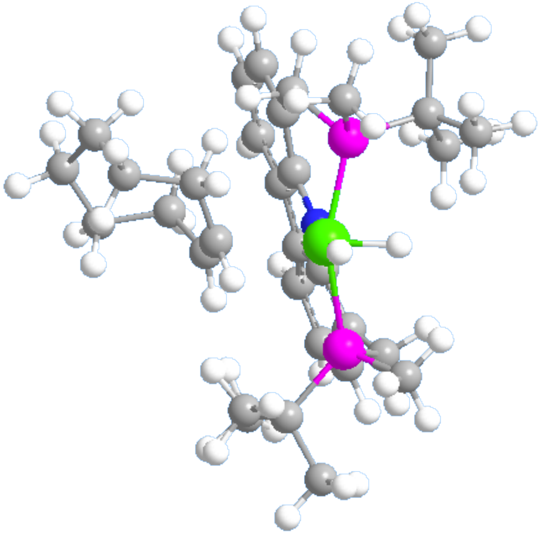

C	2.13380000	-3.73830000	3.00470000
C	2.55540000	-3.16090000	1.80050000
C	1.69390000	-2.48800000	0.93770000
C	0.34150000	-2.36380000	1.33260000
C	-0.09430000	-3.00770000	2.52980000
C	0.79880000	-3.68040000	3.36430000
H	2.85300000	-4.25170000	3.63410000
H	3.59900000	-3.25760000	1.50670000
H	0.44710000	-4.15830000	4.27460000
C	-1.83800000	-2.04380000	1.42100000
C	-3.19760000	-1.74050000	1.16760000
C	-4.14540000	-2.21620000	2.07220000
C	-3.81160000	-2.95540000	3.21260000
C	-2.48560000	-3.25810000	3.46920000
C	-1.50860000	-2.81860000	2.57710000
H	-5.19250000	-1.99480000	1.87580000
H	-4.59580000	-3.29710000	3.87970000
H	-2.20760000	-3.84230000	4.34230000
N	-0.70760000	-1.74230000	0.68880000
C	-3.70410000	-0.89970000	0.02400000
H	-3.78980000	0.15160000	0.33560000
H	-4.72330000	-1.20270000	-0.24340000
C	2.22860000	-2.02990000	-0.39040000
H	3.31420000	-2.18260000	-0.42390000
H	1.80650000	-2.64210000	-1.19950000
P	-2.66000000	-0.84080000	-1.47820000
P	1.82320000	-0.30970000	-0.90040000
C	-2.85680000	-2.52790000	-2.30470000
C	-3.62610000	0.25630000	-2.57980000

C	2.48570000	-0.27900000	-2.60870000
C	3.06530000	0.77220000	0.03500000
Rh	-0.51700000	-0.24290000	-0.94520000
C	3.04520000	0.41930000	1.52410000
H	2.03840000	0.37810000	1.94800000
H	3.50480000	-0.55250000	1.71610000
H	3.61060000	1.17240000	2.08450000
C	4.48690000	0.56120000	-0.49090000
H	4.61860000	0.94680000	-1.50470000
H	5.19250000	1.09740000	0.15350000
H	4.78470000	-0.49170000	-0.48390000
C	-1.98990000	-3.58200000	-1.61480000
H	-0.93220000	-3.31190000	-1.62080000
H	-2.28330000	-3.74110000	-0.57490000
H	-2.10410000	-4.53440000	-2.14480000
C	-4.31560000	-2.98750000	-2.24750000
H	-5.01360000	-2.25900000	-2.67050000
H	-4.42640000	-3.90950000	-2.82810000
H	-4.62890000	-3.21240000	-1.22450000
H	-4.59470000	-0.17260000	-2.85000000
H	3.49690000	-0.68740000	-2.67070000
C	2.67620000	2.23790000	-0.16760000
H	3.45400000	2.88920000	0.24660000
H	2.56670000	2.49490000	-1.22660000
H	1.74070000	2.48820000	0.33640000
C	-2.41530000	-2.38510000	-3.76330000
H	-2.40660000	-3.37030000	-4.24080000
H	-3.09050000	-1.74890000	-4.34230000
H	-1.40540000	-1.97040000	-3.84140000
H	1.82100000	-0.86710000	-3.24190000
H	2.48750000	0.74250000	-2.99520000
H	-3.79800000	1.21020000	-2.08080000
H	-3.05500000	0.45590000	-3.48710000
H	-0.42000000	0.72020000	-2.19660000
H	-0.21160000	-1.32660000	-2.00700000
C	-0.99970000	2.10760000	0.03590000
C	-0.67090000	1.34800000	1.10840000
H	0.38290000	1.15020000	1.27610000
H	-0.19520000	2.47160000	-0.59590000
C	-2.35000000	2.71400000	-0.21460000
H	-2.35670000	3.12610000	-1.22850000
H	-3.12430000	1.94000000	-0.20530000
C	-1.55710000	1.05350000	2.27670000
H	-2.57880000	0.82520000	1.95760000
H	-1.20310000	0.15830000	2.79290000
C	-2.78160000	3.80590000	0.79160000
H	-1.90350000	4.36290000	1.14150000

H	-3.38770000	4.53440000	0.24390000
C	-1.55970000	2.27060000	3.23330000
H	-1.29160000	1.95120000	4.24500000
H	-0.76060000	2.95690000	2.92790000
C	-3.60780000	3.28940000	1.98090000
H	-4.12400000	2.37160000	1.66860000
H	-4.41480000	4.00050000	2.18850000
C	-2.88060000	3.03800000	3.30250000
H	-3.57380000	2.50240000	3.96250000
H	-2.69010000	3.99810000	3.79770000

Sum of electronic and thermal Energies= -2097.076590
Sum of electronic and thermal Enthalpies= -2097.075645
Sum of electronic and thermal Free Energies= -2097.184145

TS-2'-H_2(cyclooctane)- 2'-H(η^2-Cy)

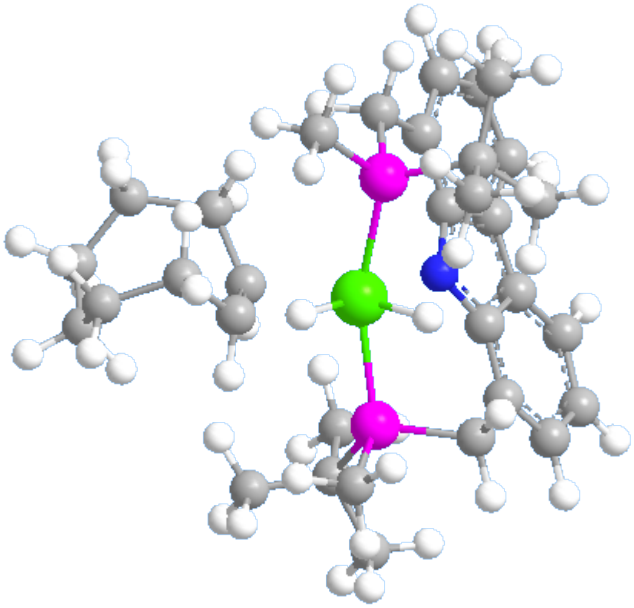

C	3.94600000	-2.52080000	3.27750000
C	2.86010000	-3.11760000	2.63040000
C	2.09400000	-2.46670000	1.66430000
C	2.43460000	-1.13250000	1.34440000
C	3.59240000	-0.56010000	1.95490000
C	4.32660000	-1.23790000	2.92650000
H	4.50180000	-3.07910000	4.02320000
H	2.60270000	-4.14660000	2.87280000
H	5.19600000	-0.77040000	3.38020000
C	2.74980000	0.81930000	0.35070000
C	2.74440000	1.94710000	-0.50300000
C	3.73000000	2.91300000	-0.31430000
C	4.72670000	2.80410000	0.66260000
C	4.76220000	1.68790000	1.47890000
C	3.79090000	0.69970000	1.31730000
H	3.72250000	3.78190000	-0.96920000
H	5.47100000	3.58680000	0.76240000
H	5.53790000	1.57350000	2.23100000
N	1.89230000	-0.26550000	0.41120000
C	1.72680000	2.16680000	-1.58190000
H	1.84840000	1.45840000	-2.40890000
H	1.82790000	3.17560000	-1.99650000
C	0.99360000	-3.26730000	1.02100000
H	1.30890000	-4.31140000	0.91550000
H	0.11630000	-3.29980000	1.67510000
P	0.02320000	1.88550000	-0.97530000
P	0.36780000	-2.66760000	-0.59150000
Rh	-0.08160000	-0.40440000	-0.51470000

H	0.62600000	-0.42230000	-1.96530000
H	-1.53740000	-0.54710000	-1.23730000
C	-0.99910000	2.39170000	-2.40600000
H	-2.05240000	2.16000000	-2.23760000
H	-0.90390000	3.45690000	-2.63220000
H	-0.66690000	1.81500000	-3.27130000
C	-1.02730000	-3.82210000	-0.87730000
H	-1.60930000	-3.50790000	-1.74510000
H	-0.68910000	-4.85030000	-1.02940000
H	-1.68440000	-3.80630000	-0.00520000
C	-0.26720000	3.24780000	0.30390000
C	0.43290000	2.88690000	1.62050000
H	0.26570000	1.84720000	1.91360000
H	1.51220000	3.03590000	1.56030000
H	0.05270000	3.53170000	2.42090000
C	-1.77530000	3.36400000	0.54290000
H	-1.96740000	4.15410000	1.27690000
H	-2.32610000	3.62690000	-0.36530000
H	-2.20200000	2.44230000	0.94780000
C	0.26900000	4.59190000	-0.19140000
H	0.02540000	5.37080000	0.53990000
H	1.35690000	4.57830000	-0.29570000
H	-0.16910000	4.90000000	-1.14570000
C	1.64260000	-3.22180000	-1.87150000
C	2.86100000	-2.29790000	-1.85370000
H	2.58790000	-1.26180000	-2.06150000
H	3.38100000	-2.32050000	-0.89250000
H	3.56710000	-2.63000000	-2.62310000
C	0.97450000	-3.15940000	-3.24640000
H	0.56130000	-2.16610000	-3.44420000
H	1.71600000	-3.37300000	-4.02320000
H	0.17210000	-3.89580000	-3.35200000
C	2.11490000	-4.65080000	-1.59190000
H	1.29530000	-5.37080000	-1.51140000
H	2.75130000	-4.98490000	-2.41810000
H	2.71710000	-4.70970000	-0.68170000
C	-1.55810000	-0.29480000	1.20520000
H	-1.38000000	0.66650000	1.68050000
C	-2.28690000	-0.24220000	-0.03120000
H	-2.56860000	0.76390000	-0.35330000
C	-1.74400000	-1.42810000	2.18650000
H	-1.44050000	-2.37350000	1.72830000
H	-1.03330000	-1.27140000	3.00570000
C	-3.43320000	-1.20970000	-0.25530000
H	-3.62160000	-1.36530000	-1.32340000
H	-3.16340000	-2.18990000	0.14350000
C	-3.15430000	-1.67860000	2.77240000

H	-3.67300000	-2.42850000	2.16220000
H	-3.03070000	-2.15880000	3.74980000
C	-4.72700000	-0.67360000	0.40350000
H	-5.25900000	-0.07250000	-0.34320000
H	-5.38970000	-1.52050000	0.62020000
C	-4.09170000	-0.48870000	2.93980000
H	-3.63420000	0.26140000	3.59690000
H	-4.97210000	-0.85200000	3.48370000
C	-4.56690000	0.20080000	1.65150000
H	-3.90370000	1.03800000	1.41230000
H	-5.53790000	0.66740000	1.85260000

Sum of electronic and thermal Energies= -2097.056381
Sum of electronic and thermal Enthalpies= -2097.055437
Sum of electronic and thermal Free Energies= -2097.160742

****** 1 imaginary frequencies (negative Signs) ******
Diagonal vibrational polarizability:
39.5593897 28.3446869 30.5213456
Harmonic frequencies (cm**-1), IR intensities (KM/Mole), Raman scattering activities (A**4/AMU), depolarization ratios for plane and unpolarized incident light, reduced masses (AMU), force constants (mDyne/A), and normal coordinates:

	1	2	3
	A	A	A
Frequencies --	-585.0617	22.6331	37.2970
Red. masses --	1.3190	4.5402	4.5678
Frc consts --	0.2660	0.0014	0.0037
IR Inten --	0.4104	0.1500	0.3091

2'-H(η^2-Cy)

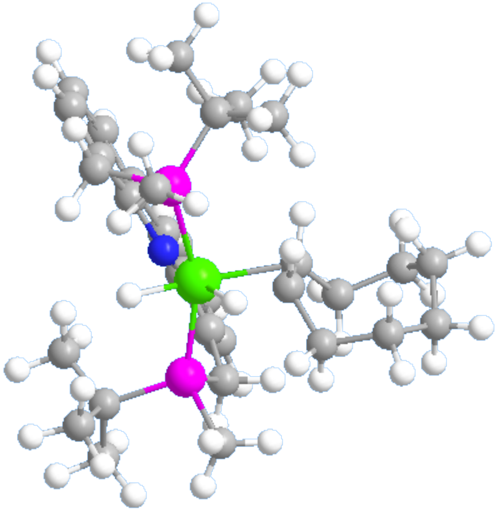

C	2.70690000	-3.25180000	3.33740000
C	1.55850000	-3.55010000	2.59750000
C	1.08260000	-2.74150000	1.56640000
C	1.79900000	-1.55530000	1.28130000
C	3.01250000	-1.29940000	1.99000000
C	3.45070000	-2.12910000	3.02070000
H	3.02550000	-3.92110000	4.12940000
H	1.01370000	-4.46610000	2.81590000
H	4.37370000	-1.90520000	3.54800000
C	2.70950000	0.22360000	0.32280000
C	3.06490000	1.30330000	-0.51820000
C	4.25480000	1.97320000	-0.24630000
C	5.10890000	1.61350000	0.80330000
C	4.78160000	0.54220000	1.61500000
C	3.59640000	-0.15330000	1.37280000
H	4.52810000	2.80700000	-0.88980000
H	6.02580000	2.16970000	0.96560000
H	5.43750000	0.23820000	2.42600000
N	1.59730000	-0.59790000	0.30210000
C	2.21240000	1.77950000	-1.65580000
H	2.18390000	1.05580000	-2.47790000
H	2.60680000	2.71940000	-2.05670000
C	-0.12760000	-3.22920000	0.81420000
H	-0.10570000	-4.32280000	0.74540000
H	-1.04700000	-2.98760000	1.35950000
P	0.46220000	1.97140000	-1.14820000
P	-0.38440000	-2.50400000	-0.84440000
C	0.47490000	3.38220000	0.11160000
C	-0.30860000	2.71240000	-2.63340000
C	-1.96520000	-3.29960000	-1.32670000
C	0.85160000	-3.35660000	-1.99580000

C	-2.42820000	0.43600000	-0.22440000
C	-1.59710000	0.26990000	0.97320000
H	-2.60470000	1.48780000	-0.48410000
H	-1.24890000	1.21460000	1.38790000
Rh	-0.26020000	-0.20770000	-0.70430000
H	-1.83530000	0.12540000	-1.29710000
H	0.39080000	-0.39240000	-2.19690000
C	1.35460000	4.54100000	-0.35980000
H	1.04970000	4.94020000	-1.33200000
H	1.28750000	5.36450000	0.36020000
H	2.40690000	4.25080000	-0.41750000
C	1.00470000	2.87860000	1.46150000
H	0.58750000	1.90960000	1.74640000
H	2.09060000	2.77120000	1.45450000
H	0.74820000	3.60200000	2.24380000
C	0.33180000	-3.19660000	-3.42630000
H	0.16400000	-2.14360000	-3.67150000
H	1.07430000	-3.58790000	-4.12940000
H	-0.59870000	-3.74540000	-3.59750000
C	2.23710000	-2.71940000	-1.89460000
H	2.20680000	-1.65410000	-2.13010000
H	2.67370000	-2.83280000	-0.89950000
H	2.90400000	-3.21350000	-2.61020000
H	-2.67180000	-3.22690000	-0.49860000
H	0.07980000	3.71000000	-2.85460000
C	0.97250000	-4.84210000	-1.64790000
H	0.01110000	-5.36450000	-1.63470000
H	1.59340000	-5.33790000	-2.40150000
H	1.45940000	-4.99330000	-0.68080000
C	-0.96560000	3.87780000	0.27380000
H	-1.37720000	4.27450000	-0.65910000
H	-1.63700000	3.09490000	0.63710000
H	-0.99170000	4.68920000	1.00930000
H	-2.39910000	-2.80010000	-2.19410000
H	-1.82910000	-4.35840000	-1.56140000
H	-1.39270000	2.77240000	-2.52200000
H	-0.09770000	2.05410000	-3.47820000
C	-1.98220000	-0.68040000	2.07510000
H	-2.54320000	-1.53500000	1.67410000
H	-1.08280000	-1.10510000	2.53790000
C	-3.71680000	-0.36210000	-0.41770000
H	-3.94540000	-0.40300000	-1.48930000
H	-3.56480000	-1.40090000	-0.11230000
C	-4.93140000	0.21220000	0.31080000
H	-5.00330000	1.28830000	0.09810000
H	-5.82400000	-0.23390000	-0.14310000
C	-2.80650000	0.00240000	3.17240000

H	-3.14530000	-0.75370000	3.89280000
H	-2.14950000	0.67780000	3.73470000
C	-4.00700000	0.79640000	2.66850000
H	-4.52080000	1.22210000	3.53670000
H	-3.65610000	1.66210000	2.08960000
C	-5.00850000	-0.00240000	1.82200000
H	-4.91000000	-1.07500000	2.04090000
H	-6.02580000	0.25890000	2.13190000

Sum of electronic and thermal Energies= -2097.066137
Sum of electronic and thermal Enthalpies= -2097.065192
Sum of electronic and thermal Free Energies= -2097.170922

TS-2'-H(η^2-Cy)- 2'-H(Cy)

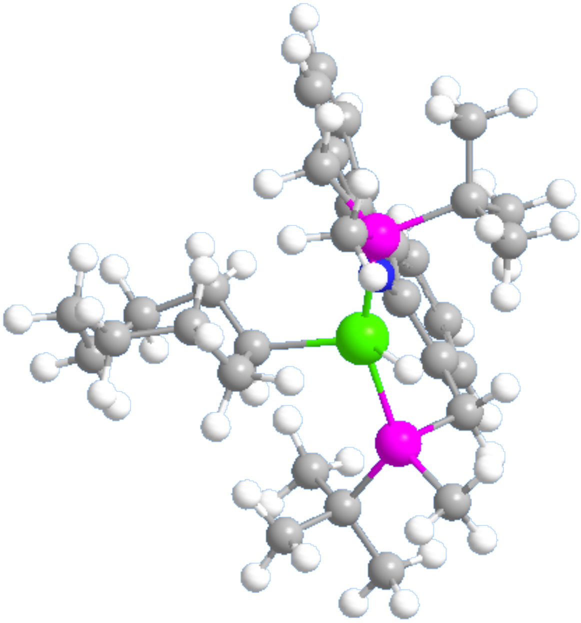

C	-3.83500000	-2.34230000	3.36170000
C	-4.09230000	-1.36840000	2.39130000
C	-3.16270000	-0.99050000	1.42400000
C	-1.90690000	-1.64260000	1.43370000
C	-1.66510000	-2.66070000	2.40550000
C	-2.61600000	-2.99700000	3.36760000
H	-4.60010000	-2.59180000	4.08900000
H	-5.06420000	-0.87990000	2.37860000
H	-2.40510000	-3.77460000	4.09630000
C	0.07970000	-2.50060000	0.94910000
C	1.25780000	-2.93760000	0.30460000
C	1.98060000	-3.96200000	0.91070000
C	1.56790000	-4.59230000	2.09050000
C	0.37000000	-4.22780000	2.67880000
C	-0.37750000	-3.19930000	2.10410000
H	2.89410000	-4.29750000	0.42370000
H	2.17250000	-5.38630000	2.51570000
H	0.00580000	-4.74090000	3.56440000
N	-0.82170000	-1.51490000	0.58430000
C	1.71910000	-2.43280000	-1.03110000
H	1.01350000	-2.73830000	-1.81600000
H	2.68200000	-2.88850000	-1.29110000
C	-3.57070000	0.08160000	0.45100000
H	-4.65720000	0.06210000	0.30580000
H	-3.34500000	1.07880000	0.85500000
P	1.80020000	-0.60760000	-1.20970000
P	-2.70560000	0.06160000	-1.16110000
C	3.35560000	-0.09370000	-0.25580000

C	2.34690000	-0.46910000	-2.95480000
C	-3.46790000	1.50630000	-1.98750000
C	-3.40690000	-1.39800000	-2.13230000
C	-0.12390000	2.52320000	-0.03030000
C	0.01980000	1.33680000	0.86860000
H	0.82330000	3.07660000	-0.11410000
H	1.06470000	1.08590000	1.04150000
Rh	-0.43950000	0.04540000	-0.82030000
H	-0.21470000	2.09820000	-1.08370000
H	-0.45250000	0.31340000	-2.46710000
C	4.61850000	-0.71120000	-0.85980000
H	4.84510000	-0.31330000	-1.85190000
H	5.47600000	-0.48200000	-0.21700000
H	4.55740000	-1.80110000	-0.93240000
C	3.24650000	-0.54460000	1.20460000
H	2.27860000	-0.31610000	1.65870000
H	3.39310000	-1.62160000	1.30590000
H	4.02020000	-0.04540000	1.79930000
C	-3.19080000	-1.11300000	-3.62000000
H	-2.14310000	-0.87800000	-3.83390000
H	-3.46130000	-1.99910000	-4.20370000
H	-3.80780000	-0.28510000	-3.97970000
C	-2.66110000	-2.67900000	-1.75830000
H	-1.58660000	-2.57740000	-1.93470000
H	-2.80000000	-2.94930000	-0.70890000
H	-3.03650000	-3.50610000	-2.37140000
H	-3.35060000	2.38550000	-1.35250000
H	3.22910000	-1.07820000	-3.16780000
C	-4.89740000	-1.57400000	-1.84290000
H	-5.47600000	-0.66400000	-2.03280000
H	-5.30670000	-2.35470000	-2.49320000
H	-5.07580000	-1.88610000	-0.81020000
C	3.47670000	1.43160000	-0.32980000
H	3.46150000	1.80000000	-1.36070000
H	2.68020000	1.93960000	0.21830000
H	4.42760000	1.74830000	0.11320000
H	-2.96470000	1.70330000	-2.93460000
H	-4.53570000	1.35450000	-2.17160000
H	2.55870000	0.57110000	-3.21030000
H	1.52150000	-0.79780000	-3.58740000
C	-0.71890000	1.26390000	2.17620000
H	-1.74030000	1.65010000	2.09090000
H	-0.81690000	0.22440000	2.49450000
C	-1.28410000	3.49670000	0.17960000
H	-1.54410000	3.92670000	-0.79470000
H	-2.17840000	2.94900000	0.50440000
C	-1.01580000	4.65730000	1.13610000

H	-0.10410000	5.17590000	0.80660000
H	-1.82600000	5.38630000	1.01490000
C	0.02840000	2.01500000	3.28730000
H	-0.57140000	1.94610000	4.20370000
H	0.96490000	1.48450000	3.50140000
C	0.33900000	3.47960000	3.00140000
H	0.81910000	3.90190000	3.89040000
H	1.09650000	3.55280000	2.20910000
C	-0.88200000	4.32930000	2.62080000
H	-1.79950000	3.83600000	2.97050000
H	-0.84490000	5.28000000	3.16290000

Sum of electronic and thermal Energies= -2097.055077
Sum of electronic and thermal Enthalpies= -2097.054133
Sum of electronic and thermal Free Energies= -2097.161752

****** 1 imaginary frequencies (negative Signs) ******
Diagonal vibrational polarizability:
32.6668398 40.1980840 51.2838769
Harmonic frequencies (cm**-1), IR intensities (KM/Mole), Raman scattering activities (A**4/AMU), depolarization ratios for plane and unpolarized incident light, reduced masses (AMU), force constants (mDyne/A), and normal coordinates:

	1	2	3
	A	A	A
Frequencies --	-291.7695	10.7952	35.5444
Red. masses --	1.5662	4.0997	4.0111
Frc consts --	0.0786	0.0003	0.0030
IR Inten --	21.4495	0.1157	0.2334

2'-H(Cy)

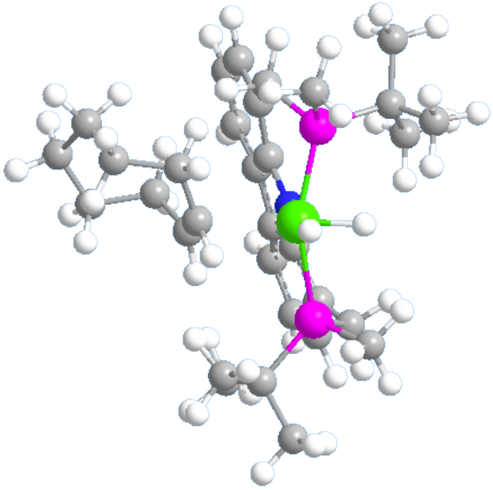

C	4.87110000	-2.13810000	2.55710000
C	3.62020000	-2.72230000	2.33090000
C	2.61640000	-2.10180000	1.58960000
C	2.89760000	-0.83450000	1.03620000
C	4.17960000	-0.25420000	1.25590000
C	5.15480000	-0.89500000	2.02000000
H	5.61320000	-2.66830000	3.14420000
H	3.41150000	-3.70450000	2.74920000
H	6.12450000	-0.43010000	2.17430000
C	2.91240000	1.05400000	-0.10850000
C	2.64350000	2.14320000	-0.96700000
C	3.62160000	3.12660000	-1.09360000
C	4.85400000	3.06090000	-0.43260000
C	5.14390000	1.97450000	0.37360000
C	4.18250000	0.97520000	0.52860000
H	3.41510000	3.97160000	-1.74690000
H	5.57910000	3.85610000	-0.56870000
H	6.10310000	1.89660000	0.87750000
N	2.10860000	-0.02710000	0.22520000
C	1.37610000	2.28140000	-1.75690000
H	1.34330000	1.54890000	-2.57630000
H	1.32140000	3.27470000	-2.21660000
C	1.30210000	-2.81340000	1.45260000
H	1.43500000	-3.88160000	1.66110000
H	0.58690000	-2.44520000	2.20480000
P	-0.13800000	1.93150000	-0.78950000
P	0.40480000	-2.59650000	-0.13380000
C	-0.22040000	3.23510000	0.56830000
C	-1.45630000	2.41960000	-1.96070000
C	-0.89610000	-3.87270000	0.10000000
C	1.46760000	-3.34630000	-1.49770000

Rh	-0.01250000	-0.31090000	-0.29790000
C	2.49210000	-2.32380000	-1.99320000
H	2.01220000	-1.39580000	-2.31710000
H	3.22530000	-2.06580000	-1.22640000
H	3.03420000	-2.74790000	-2.84600000
C	2.19530000	-4.59460000	-0.99990000
H	1.51160000	-5.35600000	-0.61180000
H	2.74780000	-5.05280000	-1.82750000
H	2.92110000	-4.35230000	-0.21830000
C	0.68460000	2.81630000	1.73020000
H	0.45340000	1.80470000	2.07960000
H	1.74270000	2.83980000	1.46230000
H	0.53740000	3.50360000	2.57090000
C	0.21110000	4.60410000	0.04550000
H	-0.38300000	4.93400000	-0.81260000
H	0.08310000	5.35600000	0.83220000
H	1.26630000	4.61040000	-0.24150000
H	-1.45910000	3.49490000	-2.15840000
H	-0.44540000	-4.85830000	0.24820000
C	0.53000000	-3.70260000	-2.65560000
H	1.12180000	-4.00050000	-3.52720000
H	-0.13470000	-4.53610000	-2.41470000
H	-0.08630000	-2.84760000	-2.95460000
C	-1.66500000	3.29020000	1.06980000
H	-1.74010000	4.00770000	1.89410000
H	-2.37260000	3.60590000	0.29740000
H	-1.99120000	2.31930000	1.45430000
H	-1.48620000	-3.63190000	0.98630000
H	-1.57650000	-3.92400000	-0.74910000
H	-1.28650000	1.89050000	-2.90070000
H	-2.43860000	2.12530000	-1.58700000
H	-0.69000000	-0.53950000	-1.66310000
C	-2.09010000	-0.37130000	0.15590000
H	-2.40690000	0.66930000	0.06520000
C	-2.98460000	-1.22050000	-0.75750000
H	-2.41860000	-2.07950000	-1.12770000
H	-3.19930000	-0.62880000	-1.65990000
C	-2.05980000	-0.69330000	1.64630000
H	-1.65420000	-1.69970000	1.81580000
H	-1.32120000	-0.01250000	2.10120000
C	-3.36340000	-0.58240000	2.47390000
H	-3.91480000	-1.52580000	2.42230000
H	-3.07300000	-0.48930000	3.52720000
C	-4.29510000	-1.76930000	-0.18320000
H	-4.71260000	-2.46850000	-0.91840000
H	-4.05980000	-2.39580000	0.68300000
C	-5.39320000	-0.75250000	0.16140000

H	-5.94710000	-0.52740000	-0.75840000
H	-6.12450000	-1.23120000	0.82690000
C	-4.32920000	0.55650000	2.15430000
H	-5.13740000	0.49700000	2.89460000
H	-3.84390000	1.52260000	2.34340000
C	-4.96320000	0.58710000	0.75850000
H	-5.84940000	1.23100000	0.81060000
H	-4.29960000	1.09020000	0.04730000

Sum of electronic and thermal Energies= -2097.090847
Sum of electronic and thermal Enthalpies= -2097.089903
Sum of electronic and thermal Free Energies= -2097.197183

TS-2'-H(Cy)-2’

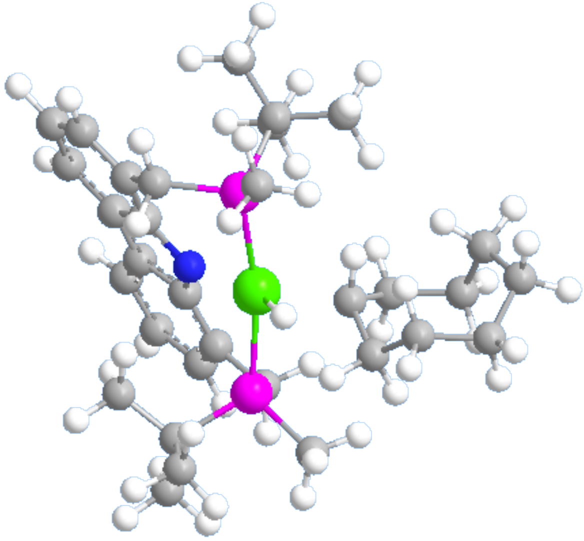

C	5.10370000	-2.18390000	2.67470000
C	3.84720000	-2.75740000	2.45340000
C	2.85060000	-2.13930000	1.70100000
C	3.14100000	-0.87930000	1.13120000
C	4.43360000	-0.31620000	1.33870000
C	5.40140000	-0.95580000	2.11340000
H	5.83900000	-2.71240000	3.27190000
H	3.62790000	-3.73290000	2.88200000
H	6.37720000	-0.49990000	2.25530000
C	3.19170000	0.99370000	-0.04710000
C	2.94020000	2.08630000	-0.90620000
C	3.93940000	3.04560000	-1.04800000
C	5.17570000	2.95740000	-0.39690000
C	5.44330000	1.87380000	0.41980000
C	4.46090000	0.89710000	0.58870000
H	3.74410000	3.89210000	-1.70290000
H	5.91780000	3.73450000	-0.54480000
H	6.40080000	1.78050000	0.92430000
N	2.36990000	-0.06660000	0.30650000
C	1.66020000	2.25890000	-1.66500000
H	1.58650000	1.53300000	-2.48710000
H	1.61660000	3.25720000	-2.11550000
C	1.54220000	-2.85890000	1.54870000
H	1.68040000	-3.93060000	1.73480000
H	0.81680000	-2.50850000	2.29880000
P	0.17140000	1.93580000	-0.64910000
P	0.68450000	-2.58420000	-0.04670000
C	0.13940000	3.25770000	0.69340000
C	-1.15090000	2.45900000	-1.80810000
C	-0.68260000	-3.79640000	0.12460000

C	1.74480000	-3.35840000	-1.40160000
Rh	0.29190000	-0.31750000	-0.15450000
C	2.80960000	-2.36710000	-1.87430000
H	2.36440000	-1.41940000	-2.19110000
H	3.54340000	-2.14380000	-1.09710000
H	3.34620000	-2.79960000	-2.72630000
C	2.42140000	-4.63500000	-0.90460000
H	1.70700000	-5.37250000	-0.52500000
H	2.96410000	-5.11010000	-1.72930000
H	3.14880000	-4.42310000	-0.11560000
C	1.04420000	2.81510000	1.84650000
H	0.78850000	1.80920000	2.19590000
H	2.09910000	2.80720000	1.56400000
H	0.92890000	3.50730000	2.68820000
C	0.59930000	4.61410000	0.16430000
H	0.00530000	4.95580000	-0.68950000
H	0.49880000	5.37250000	0.94900000
H	1.65130000	4.59260000	-0.13370000
H	-1.08120000	3.52260000	-2.05310000
H	-0.29780000	-4.81730000	0.20380000
C	0.81250000	-3.67180000	-2.57600000
H	1.40560000	-3.98410000	-3.44170000
H	0.11370000	-4.48200000	-2.35180000
H	0.23300000	-2.79130000	-2.87530000
C	-1.30080000	3.34780000	1.20350000
H	-1.34900000	4.03080000	2.05840000
H	-1.99350000	3.72540000	0.44560000
H	-1.66830000	2.37450000	1.54610000
H	-1.24710000	-3.57500000	1.03310000
H	-1.36780000	-3.74270000	-0.72100000
H	-1.04140000	1.88220000	-2.72910000
H	-2.14540000	2.25960000	-1.40560000
H	-1.00610000	-0.56220000	-1.00830000
C	-1.93050000	-0.40320000	0.13560000
H	-2.25620000	0.61940000	-0.07000000
C	-1.86060000	-0.59110000	1.65610000
H	-1.24670000	0.21140000	2.08440000
H	-1.33470000	-1.52170000	1.89910000
C	-3.22560000	-0.61950000	2.36180000
H	-3.71580000	-1.57540000	2.14420000
H	-3.04710000	-0.63450000	3.44170000
C	-2.95560000	-1.33980000	-0.55320000
H	-2.56390000	-1.64900000	-1.52880000
H	-3.05340000	-2.27140000	0.01890000
C	-4.36230000	-0.75490000	-0.79670000
H	-4.74670000	-1.22220000	-1.70940000
H	-4.27730000	0.31450000	-1.03940000

C	-4.16050000	0.53840000	1.98330000
H	-4.34600000	1.18190000	2.84920000
H	-3.66290000	1.18880000	1.25450000
C	-5.49780000	0.09590000	1.39630000
H	-6.01350000	0.97920000	0.99980000
H	-6.14470000	-0.28730000	2.19550000
C	-5.41240000	-0.96790000	0.30020000
H	-5.24590000	-1.95660000	0.74650000
H	-6.40080000	-1.03800000	-0.16690000

Sum of electronic and thermal Energies= -2097.073819
Sum of electronic and thermal Enthalpies= -2097.072874
Sum of electronic and thermal Free Energies= -2097.180185

****** 1 imaginary frequencies (negative Signs) ******
Diagonal vibrational polarizability:
65.8389287 30.3665444 28.9113256
Harmonic frequencies (cm**-1), IR intensities (KM/Mole), Raman scattering activities (A**4/AMU), depolarization ratios for plane and unpolarized incident light, reduced masses (AMU), force constants (mDyne/A), and normal coordinates:

	1	2	3
	A	A	A
Frequencies --	-592.1426	22.1503	33.3440
Red. masses --	1.1882	4.3201	4.3865
Frc consts --	0.2455	0.0012	0.0029
IR Inten --	329.6642	0.1954	0.2146
